# Cancer immunotherapy targeting murine myeloid cells requires endosomal pattern recognition

**DOI:** 10.1038/s41467-026-75543-2

**Published:** 2026-07-27

**Authors:** Yueyun Pan, Shengduo Pei, Heng Liang, Kajsa Westberg, Lars Nitschke, Xinsong Chen, Yinda Yu, Ning He, Qirong Lin, Li Lei, Anton Gisterå, Johan Hartman, Jeffrey V. Ravetch, Tak W. Mak, Tracy L. McGaha, Mikael C. I. Karlsson

**Affiliations:** 1 Department of Microbiology, Tumor and Cell Biology, Karolinska Institutet, Solna Campus, Stockholm, Sweden; 2 Division of Genetics, Department of Biology, University of Erlangen, Erlangen, Germany; 3 Department of Oncology-Pathology, Karolinska Institutet, Stockholm, Sweden; 4 Department of Medicine Solna, Center for Molecular Medicine, Karolinska University Hospital, Karolinska Institutet, Stockholm, Sweden; 5 Department of Cell and Molecular Biology, Karolinska Institutet, Solna Campus, Stockholm, Sweden; 6 Laboratory of Molecular Genetics and Immunology, Rockefeller University, New York, NY USA; 7 Princess Margaret Cancer Centre, University Health Network, Toronto, ON Canada

**Keywords:** Immunotherapy, Tumour immunology, Innate immunity

## Abstract

Immunotherapy is now an established and efficient treatment option for many cancer patients. However, a proportion of patients still experience poor outcomes due to treatment resistance. Thus, a clear understanding of key mechanisms of resistance is needed for the development of new treatments. Here, we employ mouse models to explore an immunotherapeutic approach based on anti-MARCO (αMARCO) and anti-PD-L1 (αPD-L1) antibody-mediated targeting of tumor-associated macrophages (TAM). We demonstrate that effective immunotherapy relies on a functional endosomal pattern recognition machinery. We determine that endosomal Toll-like receptors (TLR), specifically TLR9, precondition macrophages to respond to αMARCO treatment by regulating the transcription of inflammasome components. Absence of TLRs renders TAMs unresponsive to treatment while retaining an immunosuppressive phenotype. Thus, we uncover the intracellular TLR signalling as a feature of immunotherapy efficacy, required to sensitise TAMs to treatment, and indicate that TLR targeting could be exploited to improve immunotherapeutic outcomes.

## Abstract (web summary)

Tumor-associated macrophages (TAM) have key roles in cancer progression due to their immunosuppressive and anti-inflammatory phenotype, and thus represent attractive therapeutic targets. Here, the authors employ preclinical mouse models and identify endosomal TLR9 as a determinant of the therapeutic response to anti-MARCO-mediated anti-tumor responses by priming macrophages to a pre-polarized, pro-inflammatory phenotype.

## Introduction

Myeloid cells in all tissues play a key role in regulating other immune cells, both during an immune response and during tissue homeostasis. These are very functionally plastic cells and can display various polarization states that range from pro-inflammatory to anti-inflammatory in a continuum of gene expression and receptor expression patterns^1^. Within the tumor microenvironment (TME), they exist in a range of phenotypes, including TAMs and myeloid-derived suppressor cells (MDSCs), which reside in different parts of the tumor^2^. Within the tumor, the prevailing polarization state is anti-inflammatory, and an increased presence of macrophages is generally associated with poor prognosis^1^. This fact and the plasticity of this cell type have made TAMs an attractive target, either for enhancing anti-tumor responses by blocking their anti-inflammatory function or by enhancing their capacity to support tumor cell killing^3^. We previously demonstrated that targeting the scavenger receptor MARCO (macrophage receptor with collagenous structure) using antibodies can reprogram TAMs towards a pro-inflammatory state^4^. This improves the efficacy of checkpoint therapy and facilitates tumor cell destruction by natural killer (NK) cells and T cells^5,6^. Taken together, this indicates that specific targeting of TAM subpopulations is a viable strategy for enhancing immunotherapy, and several new strategies are currently being developed^7^. Macrophages express an array of pattern recognition receptors besides scavenger receptors that can regulate their polarization states. Key pattern recognition receptors include TLR3, 7, and 9, which are all endosomal receptors recognizing RNA or DNA ligands. These ligands can be taken up via phagocytosis and/or generated through nucleic acid metabolism in the endosomal compartments^8^. These TLRs can also become constitutively activated through lysosomal stress responses, providing a constant supply of ligands^9^. The functions of TLR3, 7, and 9 are connected as they all depend on a protein called UNC93B1, which is expressed in the endoplasmic reticulum (ER) and mediates stabilization and transport of these proteins to endosomes^10^. Their impact on macrophage polarization and function has mostly been studied in the context of autoimmunity or infection, where their connection to inflammasome activation is important for disease etiology^11,12^. Therefore, one unresolved question is how endosomal TLRs are involved in regulating macrophage polarization and function in the tumor context upon immunotherapy. TLR-mediated activation of TAMs also connects to another important regulatory system affecting their state of polarization, including the NLRP3 inflammasome^13^. This intracellular sensor of inflammation assembles in the cytosol to generate active Caspase-1 that in turn cleaves pro-forms of cytokines, including interleukin (IL)−1β. Since TAMs experience and reside in a dynamic microenvironment within the tumor, giving them access to both external and internal ligands, we hypothesize that endosomal TLRs would influence TAM polarization. In addition, this in turn would affect the efficacy of immunotherapy targeting myeloid cells. Here, we use two different tumor models and observe that αMARCO and αPD-L1 antibody treatment reduces tumor growth and increases T cell and NK cell infiltration in wild-type (WT) mice. However, these treatment effects are absent in mice lacking endosomal TLR signaling, where we find no reduction in tumor growth or infiltration of cytotoxic cells. Direct targeting of T cells using anti-PD-1 (αPD-1) is, however, unaffected. Investigating human melanoma and breast cancer samples, we find a correlation between MARCO or PD-L1 expression, with endosomal TLRs. Levareging this information, we observe that the loss of the treatment effect is due to endosomal TLRs’ impacts on TAM polarization, and in the case of αMARCO antibody treatment, it is specific for TLR9. Moreover, we uncover that TLR9 is needed to prepare TAMs to respond to αMARCO by regulating transcription of the inflammasome machinery and readying the macrophages to respond to treatment. Our findings extend the current understanding of the molecular requirements for efficient myeloid cell targeting for cancer immunotherapy and pave the way for developing more effective combinatory and targeted treatments for patients.

## Results

### Anti-MARCO antibody-mediated anti-tumor immune responses require endosomal TLR signaling

**Fig. 1:**
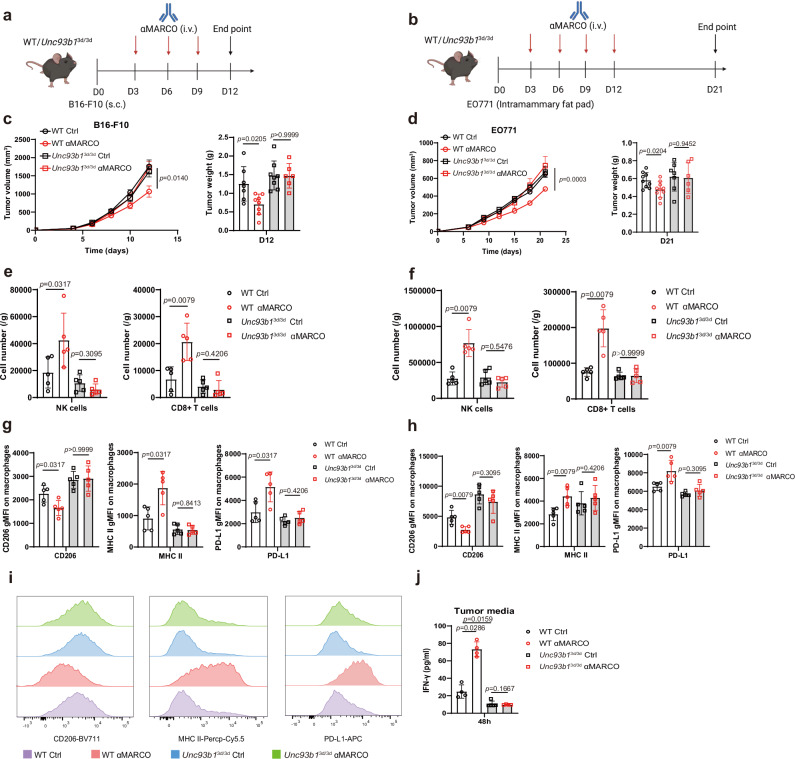
Anti-MARCO antibody-mediated anti-tumor immune responses require endosomal TLR. **a** Scheme of the murine tumor models used. B16-F10 tumor cells were injected into WT and *Unc93b1*^3d/3d^ mice. Red: with αMARCO treatment; Black: with isotype control (Ctrl) treatment. Figure created in BioRender. Liang, H. (2026) https://BioRender.com/bc60ccy. **b** EO771 tumor cells were injected into WT and *Unc93b1*^3d/3d^ mice. Figure created in BioRender. Liang, H. (2026) https://BioRender.com/c3shf71. **c** B16-F10 tumor growth curves (left) and tumor weight at day 12 (right) as described in (**a**). WT Control *n* = 7, WT αMARCO *n* = 8, *Unc93b1*^3d/3d^ Ctrl *n* = 8, *Unc93b1*^3d/3d^ αMARCO *n* = 6. **d** EO771 tumor growth curves (left) and tumor weight at day 21 (right) as described in (**b**). WT Ctrl *n* = 9, WT αMARCO *n* = 10, *Unc93b1*^3d/3d^ Ctrl *n* = 7, *Unc93b1*^3d/3d^ αMARCO *n* = 6. **e** Representative graphs showing absolute number per gram of NK and CD8^+^T cells infiltration in B16-F10 tumors as described in (**a**). *n* = 5 mice per group. **f** Representative graphs showing absolute number per gram of NK and CD8^+^T cells infiltration in EO771 tumors as described in (**b**). *n* = 5 mice per group. **g** Expression of CD206, MHCII and PD-L1 on TAMs in B16-F10 tumors as described in (**a**). *n* = 5 mice per group. **h** Expression of CD206, MHCII and PD-L1 on TAMs in EO771 tumors as described in (**b**). *n* = 5 mice per group. **i** Representative histograms of CD206, MHC II and PD-L1 on TAMs in B16-F10 tumors from (**g**). *n* in (**c**–**h**) is the number of mice pooled from two (**c**, **d**) independent experiments and from one experiment (**e**–**h**). **j** IFN-γ levels detected in the supernatant of control or αMARCO-treated B16-F10 tumor-conditioned media cultured for 48 h. WT Ctrl *n* = 4, WT αMARCO *n* = 4, *Unc93b1*^3d/3d^ Ctrl *n* = 5, *Unc93b1*^3d/3d^ αMARCO *n* = 4. *n* is the number of mice pooled from two independent experiments. Data are summarized from two independent experiments (**c**, **d**). Dots represent biological replicates (**c**–**h**, **j**). Bar plots show mean ± s.e.m. *P* values were calculated by two-side Mann–Whitney *U* test.

The scavenger receptor MARCO is known to modulate TLR function in myeloid cells^14^. In addition, TLR engagement enhances the effect of immunotherapy for cancer^15^. Upon engagement with specific antibodies functioning as agonists, MARCO is quickly internalized. We hypothesized that endosomal pattern recognition was required for MARCO-mediated cancer immunotherapy. To test this, we used mice with a mutated UNC93B1 (*Unc93b1*^3d/3d^) protein, which lack functional TLR 3, 7, and 9. Analysis of MARCO expression in the peritoneum, spleen, and tumor tissues from melanoma (B16-F10) revealed that homeostatic MARCO expression was independent of endosomal TLR signaling (Supplementary Fig. 1a–c). MARCO expression was detected at the correct locations—the marginal zone of the spleen, while in tumors expression was absent in hypoxic areas but predominantly localized near blood vessels and tumor margins as we have previously reported (Supplementary Fig. 1d)^5^. In addition, we investigated the expression of FcγRIIb as we have previously shown that the anti-MARCO effect is partly dependent on this receptor. We found that the expression was not changed between WT and *Unc93b1*^3d/3d^ in either peritoneal macrophages or TAMs (Supplementary Fig. 1e). It is known that targeting MARCO using antibodies reprogram TAMs towards a pro-inflammatory cellular state and block tumor growth in syngeneic and orthotopic cancer models^4^. To investigate the requirement for endosomal TLRs for immunotherapy, we next inoculated WT and *Unc93b1*^3d/3d^ mice with B16-F10 melanoma s.c or EO771 breast cancer cells orthotopically in the mammary fat pad and treated with control or αMARCO antibodies, respectively (Fig. 1a, b). In WT mice, αMARCO antibody treatment significantly inhibited the tumor growth in both models, whereas *Unc93b1*^3d/3d^ mice did not exhibit a response (Fig. 1c, d and Supplementary Fig. 1f). Analysis of the TME from both models revealed that, in αMARCO-treated tumors, there was an influx of CD8^+^ T cells and NK cells both in frequency and absolute numbers, but this was absent in tumors from *Unc93b1*^3d/3d^ mice (Fig. 1e, f and Supplementary Fig. 1g, h). Furthermore, αMARCO treatment in WT mice significantly increased the number of macrophages within the TME (Supplementary Fig. 1i, j). These macrophages exhibit an increased anti-tumor phenotype (increased MHC II expression and reduction in CD206 expression). In contrast, these phenotypic changes were not detected in *Unc93b1*^3d/3d^ mice (Fig. 1g–i). Interestingly, macrophages from WT mice treated with αMARCO upregulated PD-L1 expression, a process associated with IFN-γ induction. However, this PD-L1 upregulation was absent in *Unc93b1*^3d/3d^ macrophages (Fig. 1g–i). We detected production of IFN-γ in tumor-conditioned media from WT mice treated with αMARCO, whereas this was absent from *Unc93b1*^3d/3d^ mice in the B16-F10 model (Fig. 1j). No significant correlation between treatment responses and other immune cells, including neutrophil or B cell numbers, was observed (Supplementary Fig. 1i, j). Together, these results show that αMARCO treatment of cancer requires endosomal TLRs.

### Single-cell RNA sequencing reveals changes in macrophage polarization after αMARCO treatment that depend on endosomal TLRs

**Fig. 2:**
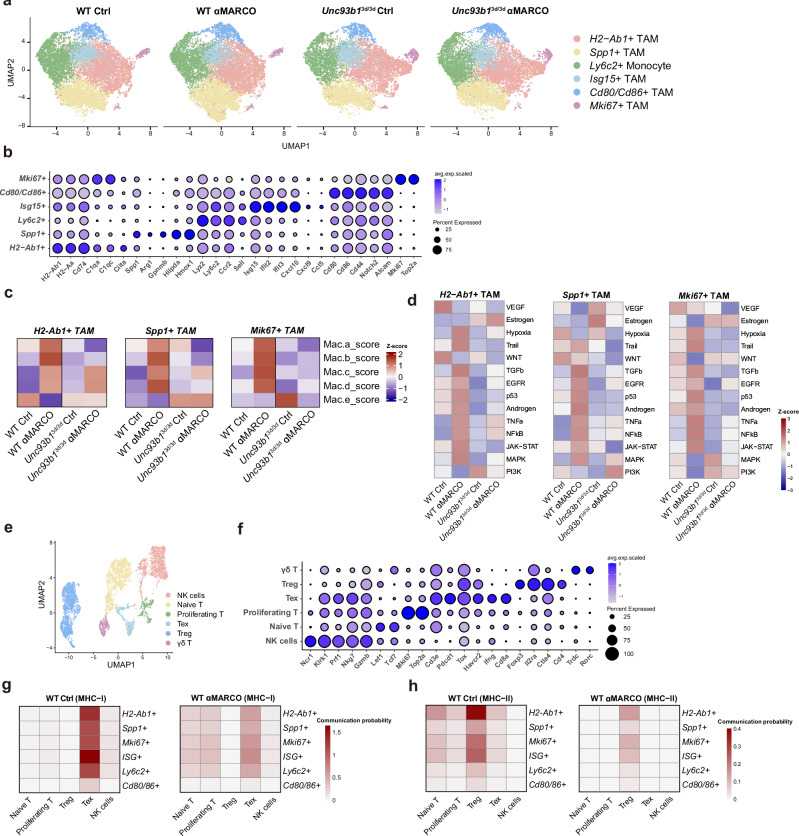
Single-cell RNA sequencing reveals that macrophage repolarization upon anti-MARCO treatment requires endosomal TLR signaling. **a** UMAP visualization of annotated cell clusters within the monocyte/macrophage compartment. Data are derived from scRNA-seq of EO771 tumor-bearing WT and *Unc93b1*^3d/3d^ mice treated with either control or αMARCO. Colors represent distinct cell subpopulations based on marker gene expression. **b** Dot plot showing the expression profiles of selected marker genes across the six annotated monocyte and macrophage clusters. Dot size represents the percentage of cells expressing the respective gene, and color represents the scaled average expression level within each cluster. **c** Heatmaps showing the normalized polarization scores (*Z*-scores) of five predefined macrophage states (Mac.a to Mac.e) across the *H2-Ab1*+, *Spp1*+, and *Mki67* + TAM subclusters from WT and *Unc93b1*^3d/3d^ tumor-bearing mice under control or αMARCO treatment. Polarization scores for each treatment group were calculated using Scupa (Single-cell unified polarization assessment). IFN-α and IFN-β induced a “Mac-a” state; IFN-γ induced a “Mac-b” state; IL-1 induced a “Mac-c” state; TNF induced a “Mac-d” state; IL-4 and IL-13 induced a “Mac-e” state. **d** Heatmaps showing the normalized pathway activity scores (*Z*-scores) across the *H2-Ab1*+, *Spp1*+, and *Mki67* + TAM subclusters from WT and *Unc93b1*^3d/3d^ tumor-bearing mice under control or αMARCO treatment. Pathway activities for each group were inferred using PROGENy. **e** UMAP visualization of annotated lymphoid cell clusters from the scRNA-seq data of EO771 tumor-bearing mice. Distinct colors represent the six annotated subpopulations: NK cells, Naive T cells, Proliferating T cells, exhausted T cells (Tex), regulatory T cells (Treg), and γδ T cells. **f** Dot plot showing the expression profiles of selected marker genes across the six annotated clusters within the lymphoid compartment. Dot size represents the percentage of cells expressing the respective gene, and color represents the scaled average expression level within each cluster. **g** Heatmaps showing the communication probability of MHC-I interactions inferred by CellChat from the six monocyte/macrophage subclusters (senders) to major lymphoid cell populations (receivers). The panels compare WT tumor-bearing mice treated with control (left) or αMARCO (right). Color intensity represents the communication probability score. **h** Heatmaps showing the communication probability of MHC-II interactions inferred by CellChat from the six monocyte/macrophage subclusters (senders) to major lymphoid cell populations (receivers).

Based on the finding that when targeting MARCO-expressing macrophages endosomal TLR signaling was required for a beneficial immunotherapeutic outcome, we next investigated the phenotype of the TAMs from WT and *Unc93b1*^3d/3d^ mice. We isolated TAMs from mice bearing EO771 breast cancer and performed bulk RNA-sequencing (RNA-seq) (Supplementary Fig. 2a). Principal components analysis (PCA) showed that endosomal TLR signaling is coupled to a distinct transcriptomic profile in TAMs (Supplementary Fig. 2b). Differential gene expression analysis revealed that compared to TAMs from *Unc93b1*^3d/3d^ mice, TAMs from WT mice exhibited elevated expression of genes associated with T cells recruitment and activation, including *Ccl5*, *Il12b*, and *Cxcl9*, and reduced expression of *Mrc1* and *Mertk*, which are markers of immunosuppressive macrophages. Gene Ontology (GO) pathway enrichment analyses also showed highly enrichment of T cell activation in TAMs from WT mice. On the other hand, Kyoto Encyclopedia of Genes and Genomes (KEGG) pathway enrichment analyses showed that TAMs were also enriched for PD-L1-related pathways, suggesting a mixed pro and anti-inflammatory phenotype (Supplementary Fig. 2c–e). To further characterize this heterogeneity at higher resolution, we next performed single-cell RNA sequencing (scRNA-seq) to investigate the phenotypic changes of TAMs before and after treatment. We isolated immune cells from the EO771 tumors that were either untreated or αMARCO treated in WT or *Unc93b1*^3d/3d^ mice. After quality control and filtering, a total of 59, 274 high-quality cells were obtained, of which 37,661 were monocytes and macrophages (Supplementary Fig. 2f, g). Unsupervised clustering identified five transcriptionally distinct TAM clusters annotated as *H2-Ab1*⁺, *Spp1*^+^, *Isg15*⁺, *Cd80/Cd86*⁺, *Mki67*⁺, and one additional cluster of *Ly6c2*^+^ monocytes (Fig. 2a, b). To investigate changes in TAM polarization, we used scores defined in the framework described by Cui and co-workers^16,17^. The analysis showed that among the TAM clusters *H2-Ab1*^+^, *Spp1*^+^ and *Mki67*^+^ were significantly shifted towards a pro-inflammatory phenotype as a result of αMARCO treatment (Fig. 2c and Supplementary Fig. 2h). Further analysis of these subsets showed that they had an anti-tumoral transcriptional repolarization with elevation of pathway activity scores, including those connected to TNF and NF-κB whereas there was a decrease of the PI3K activity (Fig. 2d and Supplementary Fig. 2i)^18^. The analysis also showed that all these changes in connection to αMARCO treatment were largely absent in TAMs from *Unc93b1*^3d/3d^ mice. To further analyze the effect on cytotoxic lymphocytes, we clustered the lymphoid cells into 6 clusters, including NK cells, regulatory T cells (Treg), γδ T cells, and three different CD8^+^ T cell activation states: naïve (*Lef1*^high^, *Tcf7* ^high^), exhausted (*Pdcd1* ^high^, *Havcr2* ^high^), and proliferating (*Mki67* ^high^, *Top2a* ^high^) (Fig. 2e, f). Analysis of MHC-I and MHC-II ligand-receptor pairs by utilizing CellChat analysis showed that the six TAM/monocyte clusters in WT tumors was primarily directed towards exhausted T cells and regulatory T cells^19^. After αMARCO treatment, these interactions were reduced and redirected towards naïve and proliferating T cells (Fig. 2g, h). This shift in communication was not observed in tumors from *Unc93b1*^3d/3d^ mice (Supplementary Fig. 2j, k). Taken together, these data demonstrate that in the absence of endosomal TLR signaling, TAMs maintain an unresponsive and suppressive phenotype that alters their interactions with T cells.

### Anti-PD-L1 but not anti-PD-1 treatment requires endosomal TLR signaling

**Fig. 3:**
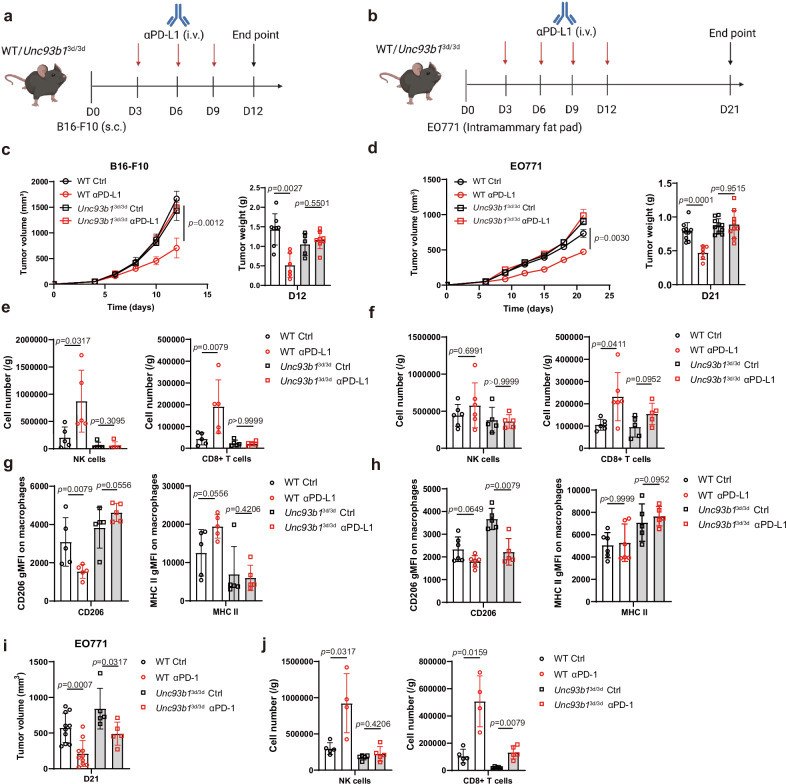
Myeloid targeting was perturbed in the absence of endosomal TLR signaling. **a** Scheme of the murine tumor models employed. B16-F10 tumor cells were injected into WT and *Unc93b1*^3d/3d^ mice. αPD-L1 treatment was administered every three days. Red: with αPD-L1 treatment; Black: with Ctrl treatment. Figure created in BioRender. Liang, H. (2026) https://BioRender.com/ax0e4oj. **b** EO771 tumor cells were injected into WT and *Unc93b1*^3d/3d^ mice. αPD-L1 treatment was administered every three days. Figure created in BioRender. Liang, H. (2026) https://BioRender.com/akjne6l. **c** B16-F10 tumor growth curves (left) and tumor weight at day 12 (right) as described in (**a**). WT Ctrl *n* = 8, WT αPD-L1 *n* = 6, *Unc93b1*^3d/3d^ Ctrl *n* = 5, *Unc93b1*^3d/3d^ αPD-L1 *n* = 8. **d** EO771 tumor growth curves (left) and tumor weight at day 21 (right) as described in (**b**). WT Ctrl *n* = 10, WT αPD-L1 *n* = 6, *Unc93b1*^3d/3d^ Ctrl *n* = 10, *Unc93b1*^3d/3d^ αPD-L1 *n* = 9. **e** Absolute number per gram of NK and CD8^+^T cells infiltration in B16-F10 tumors as described in (**a**). **f** Absolute number per gram of NK and CD8^+^T cells infiltration in EO771 tumors as described in (**b**). **g** Expression of CD206 and MHC II on TAMs in B16-F10 tumors as described in (**a**). *n* = 5 mice per group. **h** Expression of CD206 and MHC II on TAMs in EO771 tumors as described in (**b**). *n* = 6 for WT mice; *n* = 5 for *Unc93b1*^3d/3d^ mice. **i** Representative graph showing tumor volume at day 21 of the WT and *Unc93b1*^3d/3d^ mice treated with control or αPD-1 in EO771 breast cancer model. αPD-1 was injected (i.v.) into mice every three days as described before. WT Ctrl *n* = 10, WT αPD-1 *n* = 10, *Unc93b1*^3d/3d^ Ctrl *n* = 5, *Unc93b1*^3d/3d^ αPD-1 *n* = 5. **j** Absolute number per gram of NK and CD8^+^T cells infiltration in EO771 tumors from **i**. WT Ctrl *n* = 5, WT αPD-1 *n* = 4, *Unc93b1*^3d/3d^ Ctrl *n* = 5, *Unc93b1*^3d/3d^ αPD-1 *n* = 5. *n* is the number of mice from one (**e**–**h**, **j**) and two (**c**, **d**, **i**) independent experiments. Data are summarized from two independent experiments (**c**–**j**). Dots represent biological replicates (**c**–**j**). Bar plots show mean ± s.e.m. *P* values were calculated by two-side Mann–Whitney *U* test.

The gene expression analysis of TAMs lacking functional endosomal TLRs revealed that they retained a suppressive phenotype. Thus, we next investigated if there were also changes in response to checkpoint therapy targeting PD-1 or PD-L1. The sequencing data suggested that PD-L1 expression was affected by the UNC93B1 mutation. We confirmed this by FACS staining of TAMs from both B16-F10 and EO771 tumors, which showed reduced PD-L1 expression in the absence of functional UNC93B1 (Supplementary Fig. 3a, b). We also observed lower PD-L1 expression on dendritic cells (DC) but not on neutrophils. To investigate whether this influenced the treatment responsiveness to αPD-L1, we used the B16-F10 melanoma and EO771 breast cancer models and subjected them to αPD-L1 treatment (Fig. 3a, b). For both tumor models, αPD-L1 therapy was ineffective in the absence of functional UNC93B1, affecting tumor weight and volume (Fig. 3c, d). We also observed a reduced influx of both NK cells and CD8^+^ T cells in the B16-F10 model, and reduced CD8^+^ T cell numbers in the EO771 model in *Unc93b1*^3d/3d^ mice after treatment (Fig. 3e, f). In WT mice, TAMs responded to PD-L1 blockade by altering their phenotype with downregulation of CD206 and upregulation of MHC II, suggesting a more pro-inflammatory phenotype. However, in *Unc93b1*^3d/3d^ mice, these changes were absent. This was the case in the B16-F10 model, whereas this was not as prominent on day 21 in the EO771 model (Fig. 3g, h). These findings using αMARCO and αPD-L1 treatments showed that myeloid targeting was perturbed in the absence of endosomal TLRs. PD-L1 can be expressed by several types of cells in the TME, including the tumor cells themselves, which is the case for EO771 but not for B16-F10. Thus, we next explored whether direct targeting of T cells using αPD-1 treatment was affected by UNC93B1 deficiency. Using the same experimental setup, we found that unlike αPD-L1, αPD-1 treatment was effective in both WT and *Unc93b1*^3d/3d^ mice, and in both the B16-F10 melanoma model and the EO771 breast cancer model, respectively (Fig. 3i and Supplementary Fig. 3c, d). In addition, we recorded an influx of NK and CD8^+^ T cells as a result of PD-1 blocking (Fig. 3j). Together, these findings demonstrate that αPD-L1 treatment efficacy depends on endosomal TLR signaling, whereas αPD-1 treatment operates independently of this pathway. This is likely due to differences in target cell populations. Also, the UNC93B1 mutation does not affect the ability of cytotoxic cells to kill the tumor.

### The unresponsiveness of MARCO-expressing macrophages is cell intrinsic

**Fig. 4:**
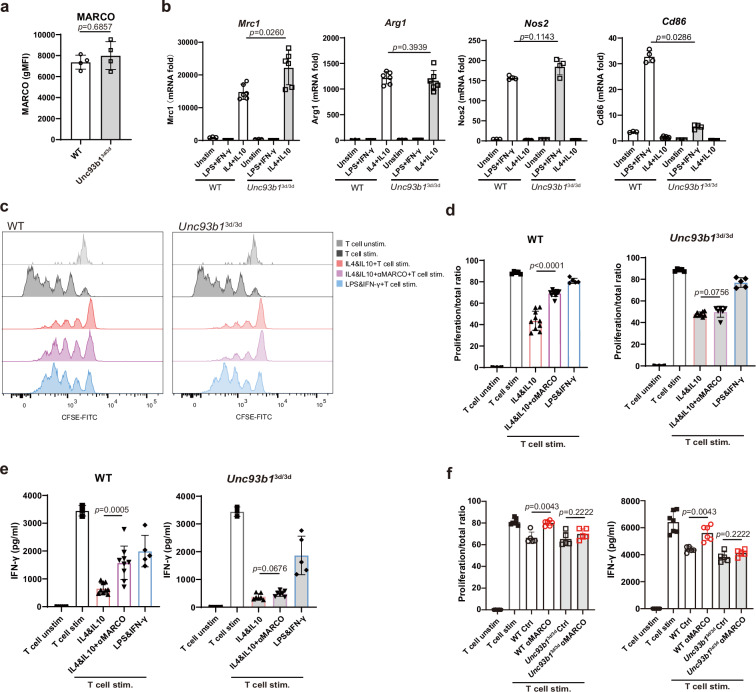
The unresponsiveness of MARCO-expressing macrophages is cell intrinsic. **a** Expression of MARCO on bone marrow derived macrophages (BMDM) stimulated with IL-4 (20 ng/ml) and IL-10 (20 ng/ml) for 48 h. *n* = 4 mice per group. **b** BMDMs were stimulated with indicated cytokines. *Mrc1*, *Arg1*, *Nos2* and *Cd86* mRNA expression were analyzed using RT-qPCR. *n* = 3 (Unstim), 4 (LSP + IFN-γ), and 6 (IL4 + IL10). **c** BMDMs from WT (left) and *Unc93b1*^3d/3d^ (right) mice were stimulated with indicated cytokines, treated with control or αMARCO antibody, and cocultured with CFSE labeled T cells from naive mice followed with CD3/CD28 T-cell activator beads for 48 h. Representative histograms of CFSE on T cells were assessed by flow cytometry. **d** Ratio of proliferating T cells out of total T cells were assessed by flow cytometry. T cell unstim *n* = 3, T cell stim *n* = 5; for WT BMDMs, IL4&IL10 + T cell stim *n* = 10, IL4&IL10 BMDM + αMARCO + T cell stim *n* = 10, LPS&IFN-γ + T cell stim *n* = 5; for *Unc93b1*^3d/3d^, IL4&IL10 + T cell stim *n* = 8, IL4&IL10 BMDM + αMARCO + T cell stim *n* = 6, LPS&IFN-γ + T cell stim *n* = 5. **e** IFN-γ levels detected in the supernatant from BMDMs and T cells coculturing system. T cell unstim *n* = 6, T cell stim *n* = 3; for WT BMDMs: IL4&IL10 + T cell stim *n* = 9, IL4&IL10 BMDM + αMARCO + T cell stim *n* = 9, LPS&IFN-γ + T cell stim *n* = 5; for *Unc93b1*^3d/3d^ BMDMs: IL4&IL10 + T cell stim *n* = 7, IL4&IL10 BMDM + αMARCO + T cell stim *n* = 7, LPS&IFN-γ + T cell stim *n* = 5. **f** Sorted TAMs from B16-F10 tumors in WT and *Unc93b1*^3d/3d^ mice treated with control or αMARCO were cocultured with CFSE labeled naive T cells in the presence of CD3/CD28 activator beads for 48 h. Ratio of proliferating T cells to total T cells (left) and IFN-γ levels in the coculture supernatant measured by ELISA (right). T cell unstim *n* = 5, T cell stim *n* = 7; WT Ctrl + T cell stim *n* = 5; WT αMARCO + T cell stim *n* = 6; *Unc93b1*^3d/3d^ Ctrl + T cell stim *n* = 5; *Unc93b1*^3d/3d^ αMARCO + T cell stim *n* = 5. Data are summarized from two independent experiments (**a**–**f**). Dots represent biological replicates (**a**, **b**, **d–f**). Bar plots show mean ± s.e.m. *P* values were calculated by Mann–Whitney *U* test.

Unlike PD-L1, we did not observe any changes in MARCO expression in the tumor, even though αMARCO treatment was also ineffective in mice lacking endosomal TLR signaling. To investigate if this was a macrophage intrinsic effect, we set up a co-culture system using bone marrow-derived macrophages (BMDM) and T cells (Supplementary Fig. 4a). BMDMs from WT or *Unc93b1*^3d/3d^ mice were polarized with IL-4 and IL-10 to give an anti-inflammatory activation state, or with IFN-γ and LPS to induce a pro-inflammatory activation state. Polarization of macrophages was confirmed both at gene and protein levels, and we observed that MARCO was equally expressed on WT or UNC93B1 deficient macrophages (Fig. 4a, b and Supplementary Fig. 4b). Investigating RNA expression levels, we found differences in anti-inflammatory marker *Mrc1* but not *Arg1*. And for pro-inflammatory markers, *Cd86* was lower, whereas *Nos2* was unchanged. However, there was no significant difference in protein levels for CD206, CD86 or MHC II (Fig. 4b and Supplementary Fig. 4b). We next tested the capacity of the in vitro derived macrophages to block T cell activation. For this experiment, anti-inflammatory BMDMs from either WT or *Unc93b1*^3d/3d^ mice were pretreated with αMARCO antibody or isotype controls, followed by incubation with purified splenic T cells. Macrophage function was evaluated by measuring T cell proliferation and cytokine secretion. Thus, we find that BMDMs from WT and *Unc93b1*^3d/3d^ mice both significantly reduced proliferation and IFN-γ production by T cells. However, when we incubated the macrophages with αMARCO, it was only macrophages from WT mice that responded by inducing T cell proliferation and IFN-γ production (Fig. 4c–e). A similar reversal of suppression by αMARCO antibodies was observed in BMDMs stimulated with tumor-conditioned media, but not in cultures with IFNγ and LPS-induced pro-inflammatory BMDMs (Supplementary Fig. 4c, d). Next, we sorted TAMs from B16-F10 tumors from either treated or untreated WT or *Unc93b1*^3d/3d^ mice. These were subsequently incubated with naïve T cells and the ability of the TAMs to block T cell proliferation and activation were measured in vitro. We found that TAMs from WT mice could significantly suppress T cell proliferation and IFNγ production, and that this effect was relieved when treating with αMARCO antibodies, whereas no such effect was observed in TAMs from tumors of *Unc93b1*^3d/3d^ mice (Fig. 4f). These results demonstrate that endosomal TLR signaling in macrophages is essential for αMARCO treatment to be efficient in inducing T cell proliferation and IFN-γ production.

### MARCO expression correlates with TLR9 activation

**Fig. 5:**
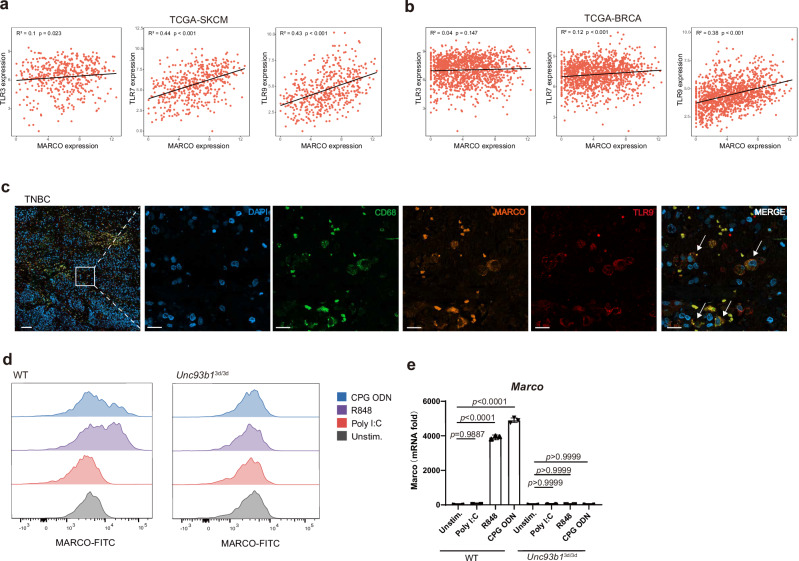
MARCO expression is correlated with TLR9 activation. **a** The FPKM expression of *MARCO* was plotted against *TLR3*, *TLR7* and *TLR9* in a subset of patients with melanoma included in the TCGA database. Correlation is shown in a xy-plot with a linear regression. *P* values were calculated by two-tailed Pearson’s correlation test. **b** The FPKM expression of *MARCO* was plotted against *TLR3*, *TLR7* and *TLR9* in a subset of patients with breast cancer included in the TCGA database. *P* values were calculated by two-tailed Pearson’s correlation test. **c** Immunofluorescence analysis of triple-negative breast cancer (TNBC) from a cohort of *n* = 3 patients. Representative immunofluorescence images are shown for a patient who received neoadjuvant chemotherapy and went for operation. Scale bars, 100 μm (far-left) and 20 μm. **d** BMDMs from WT and *Unc93b1*^3d/3d^ mice were stimulated with the indicated components. Black: without stimulation; red: Poly I:C (TLR3 agonist) (1 μg/ml); purple: R848 (TLR7 agonist) (1 μg/ml); blue: CPG ODN (TLR9 agonist) (1 μM). Representative histograms showing the MARCO expression. **e** Representative graph showing the relative gene expression of *Marco* under the indicated components stimulation. *n* = 3 mice per group from one experiment. Data are summarized from three (**d**, **e**) independent experiments. Bar plots show mean ± s.e.m. *P* values were calculated by ordinary one-way ANOVA followed by Tukey’s post hoc test (**e**).

Since we found that efficient myeloid targeting required endosomal TLRs, we next investigated samples from human tumors to determine if the gene expression of *MARCO* or *CD274* correlated with the expression of *TLR3*, *TLR7*, and *TLR9*. This was done using data from tumor biopsies of patients (The Cancer Genome Atlas (TCGA)-Skin Cutaneous Melanoma project (SKCM) and Breast Invasive Carcinoma (BRCA)). We found that in the SKCM patient group, *MARCO* expression correlated with *TLR7* and *TLR9* but not *TLR3* (Fig. 5a), whereas in the BRCA patient group, *MARCO* expression correlated with *TLR9* (Fig. 5b). We also found a positive correlation between *CD274* expression and *TLR3*, *TLR7*, and *TLR9* in both patient groups (Supplementary Fig. 5a, b). Given the association of TLR9 with DNA ligands and that MARCO mediates uptake of apoptotic cells that influence DNA-driven autoreactive immune responses^20^, we hypothesized that TLR9 was the most likely candidate of the endosomal TLRs. Next, to verify that MARCO-expressing human TAMs also expressed TLR9, we investigated tumor samples from breast cancer patients with different subtypes. Staining of the pan-macrophage marker CD68 and MARCO confirmed the TLR9 expression in MARCO^+^ macrophages across all breast cancer subtypes, including luminal A, luminal B, HER2, and triple-negative breast cancer (TNBC), by immunofluorescence imaging (Fig. 5c and Supplementary Fig. 5c). These data suggested that TLR9 was a likely candidate among the endosomal TLRs to be specifically needed for the response to αMARCO treatment. To further substantiate this, we performed in vitro experiments. It is known that MARCO is expressed at steady state by subpopulations of macrophages^21^, and it can also be upregulated on macrophages following stimulation of TLRs. To test this, BMDMs from WT and *Unc93b1*^3d/3d^ mice were stimulated with specific agonists: Poly I:C (TLR3 agonist), R848 (TLR7 agonist), and CPG ODN (TLR9 agonist). Both TLR7 and TLR9 but not TLR3 agonists markedly upregulated MARCO expression at the gene and protein levels in WT BMDMs, while this effect was absent in BMDMs from *Unc93b1*^3d/3d^ mice (Fig. 5d, e). This was different from PD-L1 which expression was upregulated by TLR3 and TLR7 ligands but not TLR9 (Supplementary Fig. 5d). Thus, the MARCO expressing phenotype of macrophages correlates with TLR9 activation, suggesting that this is a key endosomal TLR that could be connected to the absence of treatment effects using αMARCO antibodies.

### Specific deletion of TLR9 in MARCO-expressing macrophages blocks anti-MARCO-mediated immunotherapy

**Fig. 6:**
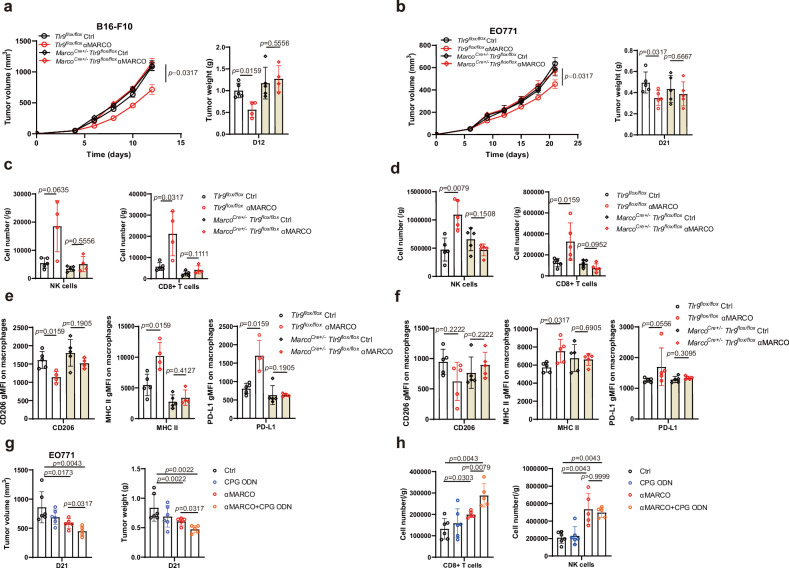
Specific deletion of TLR9 in MARCO-expressing macrophages block anti-MARCO- mediated immunotherapy. **a** B16-F10 tumor growth (left) and tumor weight at day 12 (right) in *Tlr9*^*flox/flox*^ and *Marco*^*Cre+/−*^ *Tlr9*^*flox/flox*^ mice following with a control or αMARCO treatment. Red, with αMARCO treatment; black, with isotype control (Ctrl) treatment. *Tlr9*^*flox/flox*^ Ctrl *n* = 5, *Tlr9*^*flox/flox*^ αMARCO *n* = 4, *Marco*^*Cre+/−*^ *Tlr9*^*flox/flox*^ Ctrl *n* = 5, *Marco*^*Cre+/−*^ *Tlr9*^*flox/flox*^ αMARCO *n* = 4. **b** EO771 tumor growth (left) and tumor weight at day 21 (right) in *Tlr9*^*flox/flox*^ and *Marco*^*Cre+/−*^ *Tlr9*^*flox/flox*^ mice following with a control or αMARCO treatment. *n* = 5 mice per group. **c** Absolute number per gram of NK and CD8^+^T cells infiltration in B16-F10 tumors as described in (**a**). *n* = 5 for control group, *n* = 4 for αMARCO treatment group. **d** Absolute number per gram of NK and CD8^+^T cells infiltration in EO771 tumors as described in (**b**). *n* = 5 mice per group. **e** The expression of CD206, MHC II and PD-L1 on TAMs in B16-F10 tumors as described in (**a**). **f** The expression of CD206, MHC II and PD-L1 on TAMs in EO771 tumors as described in (**b**). **g** EO771 tumor-bearing WT mice were treated with CpG ODN (20 μg) (blue), αMARCO (100 μg) (red), or a combination of αMARCO and CpG ODN (orange) every three days. Representative graphs showing tumor volume (left) and tumor weight (right) measured at day 21 post-tumor inoculation. Ctrl *n* = 6; CpG ODN *n* = 6; αMARCO *n* = 5; αMARCO + CpG ODN *n* = 5. **h** Absolute number per gram of CD8^+^T and NK cells infiltration in EO771 tumors as described in (**g**). *n* is the number of mice in one experiment (**a**–**h**). Data are summarized from two (**a**–**h**) independent experiments. Dots represent biological replicates (**a**–**h**). Bar plots show mean ± s.e.m. *P* values were calculated by two-side Mann–Whitney *U* test (**a**–**h**).

Next, to investigate the contribution of TLR9 in vivo, we generated a *Marco-Cre* mouse for targeted depletion of TLR9 from MARCO-expressing cells^22^. Using this mouse crossed to a Rosa-tdTomato reporter (ROSA)26Sor^tm14(CAG-tdTomato)Hze^/J mouse, we observed labelled macrophages in the peritoneum as well as in the TME by FACS, showing the efficacy of Cre activity (Supplementary Fig. 6a–c). Next, we crossed the *Marco-Cre* mouse with TLR9-floxed mice (*Tlr9*^*flox/flox*^)^23^ to generate conditionally targeted mice (Supplementary Fig. 6d). Investigating *Marco*^*Cre+/−*^ *Tlr9*^*flox/flo*x^ mice, we found that TLR9 was specifically knocked out in MARCO^+^ macrophages (Supplementary Fig. 6e–h). MARCO expression remained intact by analyzing in the peritoneum, spleen, and tumor tissues (Supplementary Fig. 6i, j). Next, we examined the role of TLR9 in MARCO-expressing cells by treating littermate controls and conditionally targeted mice with αMARCO in the setting of both B16-F10 and EO771 tumor models. We determined that αMARCO was not effective in reducing tumor growth within TLR9 conditionally targeted mice in both models, indicating a specific dependence on TLR9 (Fig. 6a, b). In addition, when compared to littermate controls in response to αMARCO treatment, we observed a reduced influx of NK cells and CD8^+^ T cells in TLR9 conditional knockout mice in both the B16-F10 and EO771 tumor models (Fig. 6c, d). Furthermore, TAMs displayed decreased CD206 and increased MHC II and PD-L1 expression in response to the treatment in littermate controls, but not in *Marco*^*Cre+/−*^ *Tlr9*^*flox/flox*^ mice in the B16-F10 model (Fig. 6e). In the EO771 model, we observed upregulation of MHC II on day 21, which was absent in *Marco*^*Cre+/−*^ *Tlr9*^*flox/flox*^ mice (Fig. 6f). Since we had observed that αPD-L1 treatment efficacy depends on endosomal TLR signaling, we next investigated whether αPD-L1 therapy depends on TLR9 signaling of MARCO^+^ macrophages. First, we investigated PD-L1 expression on myeloid cells, which was similar between littermate controls and the conditionally targeted mice (Supplementary Fig. 6k). Second, using the B16-F10 and EO771 tumor models, we found that αPD-L1 treatment effectively reduced tumor growth and altered the TME in both littermate controls and *Marco*^*Cre+/−*^ *Tlr9*^*flox/flox*^ mice, including the NK cells and CD8^+^ T cells influx and TAMs phenotype changes (Supplementary Fig. 6l–q). This supports that the therapeutic response to αPD-L1 does not rely on TLR9 signaling in MARCO^+^ macrophages. Dependence of TLR9 suggested that increased activation through this receptor would improve the efficacy of αMARCO treatment. To test this, we next injected αMARCO alone or in combination with TLR9 agonists for immunotherapy in the EO771 tumor model. The data showed that combinatory treatment significantly improved the treatment effect with reduced tumor volume and weight as well as increased the influx of CD8^+^ T cells to the tumor (Fig. 6g, h). This combinatory effect was also found in the B16-F10 model, where we found reduced tumor growth after injecting αMARCO together with TLR9 agonists (Supplementary Fig. 6r). Overall, these data shows that TLR9 is required for MARCO-expressing cells to respond to αMARCO treatments in vivo.

### TLR9 signaling is essential for anti-MARCO efficacy in vitro

**Fig. 7:**
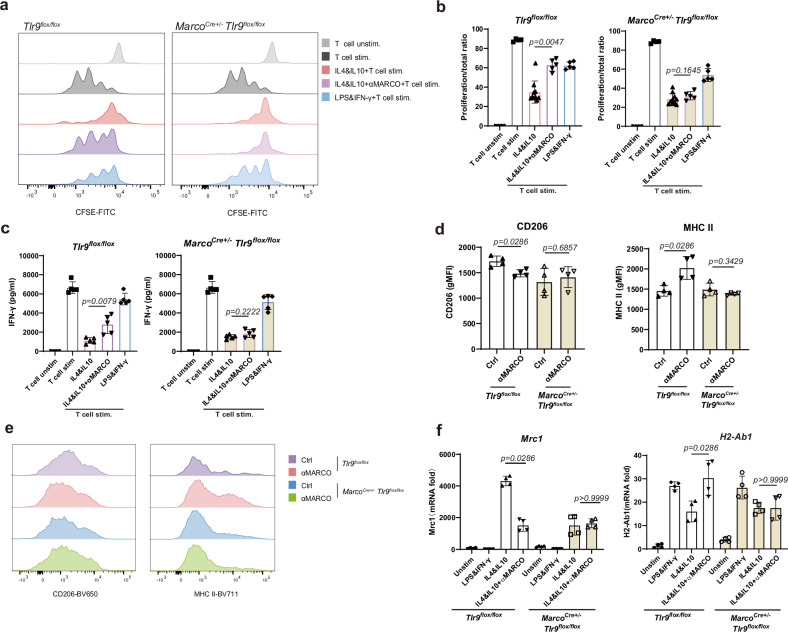
TLR9 signaling is essential for anti-MARCO efficacy in vitro. **a** BMDMs from *Tlr9*^*flox/flox*^ (left) and *Marco*^*Cre+/−*^ *Tlr9*^*flox/flox*^ mice (right) were stimulated with indicated cytokines, treated with control or αMARCO, and cocultured with CFSE labeled T cells from naive mice followed with CD3/CD28 T-cell activator beads for 48 h. Representative histograms of CFSE on T cells were assessed by flow cytometry. Grey: unstimulated T cell (no beads); black: stimulated T cell (with beads); red: IL4&IL10 stimulated BMDMs treated with isotype control; purple: IL4&IL10 stimulated BMDMs treated with αMARCO; blue: LPS&IFN-γ stimulated BMDMs treated with isotype control. **b** Representative graph showing the ratio of proliferating T cells out of total T cells was assessed by flow cytometry. T cell unstim *n* = 4, T cell stim *n* = 4; for both *Tlr9*^*flox/flox*^ and *Marco*^*Cre+/−*^ *Tlr9*^*flox/flox*^ BMDMs, IL4&IL10 + T cell stim *n* = 10, IL4&IL10 BMDM + αMARCO + T cell stim *n* = 5, LPS&IFN-γ + T cell stim *n* = 5. **c** IFN-γ levels detected in the supernatant from BMDMs and T cells coculturing system. *n* = 5 mice per group from one experiment. **d** Graphs showing the CD206 and MHCII expression of IL4&IL10 stimulated BMDMs from *Tlr9*^*flox/flox*^ and *Marco*^*Cre+/−*^ *Tlr9*^*flox/flox*^ mice treated with control or αMARCO for 10 h. *n* = 4 mice per group. **e** Representative histograms showing the CD206 and MHCII expression of IL4&IL10 stimulated BMDMs from *Tlr9*^*flox/flox*^ and *Marco*^*Cre+/−*^ *Tlr9*^*flox/flox*^ mice treated with control or αMARCO for 10 h. **f** Graphs showing the *Mrc1* and *H2-Ab1* gene expression of LPS&IFN-γ or IL4&IL10 stimulated BMDMs from *Tlr9*^*flox/flox*^ and *Marco*^*Cre+/−*^ *Tlr9*^*flox/flox*^ mice treated with control or αMARCO for 10 h. *n* = 4 mice per group. *n* is the number of mice from one (**b**, **d**, **f**) and two (**c**) independent experiments. Data are summarized from two independent experiments (**b**–**d**, **f**). Dots represent biological replicates (**b**–**d**, **f**). Bar plots show mean ± s.e.m. *P* values were calculated by two-side Mann–Whitney *U* test.

We further investigated how TLR9 deficiency modulated the function and responsiveness of MARCO-expressing macrophages in vitro using a co-culture system with T cells. At steady state, anti-inflammatory BMDMs from *Tlr9*^*flox/flox*^, *Marco*^*Cre+/−*^, and *Marco*^*Cre+/−*^ *Tlr9*^*flox/flox*^ exhibited comparable inhibitory effects on T cell proliferation and IFN-γ cytokine secretion. However, αMARCO antibody addition to the cultures rescued T cell proliferation and IFN-γ secretion in co-cultures with *Tlr9*^*flox/flox*^ and *Marco*^*Cre+/−*^ BMDMs but not in co-cultures with *Marco*^*Cre+/−*^ *Tlr9*^*flox/flox*^ BMDMs (Fig. 7a–c and Supplementary Fig. 7a, b). Similar results were obtained when using tumor-conditioned media, where we find that αMARCO restores T cell proliferation and IFNγ production in cultures with macrophages from littermate controls but not for conditionally targeted macrophages. Also, there was no effect on T cell activation when using pro-inflammatory macrophages stimulated with LPS and IFNγ (Supplementary Fig. 7a, b). This implies that TLR9 expression is needed for MARCO-expressing macrophages to respond to engagement with αMARCO and facilitate cytotoxic lymphocyte activation. This was also true for repolarization, where in the absence of TLR9, there were no changes in CD206 or MHC II expression at both mRNA and protein levels after αMARCO stimulation (Fig. 7d–f). Thus, TLR9 is intrinsically needed for repolarization of anti-inflammatory macrophages in vitro.

### Anti-MARCO promotes inflammasome activation and IL-1β secretion in a TLR9-dependent manner

**Fig. 8:**
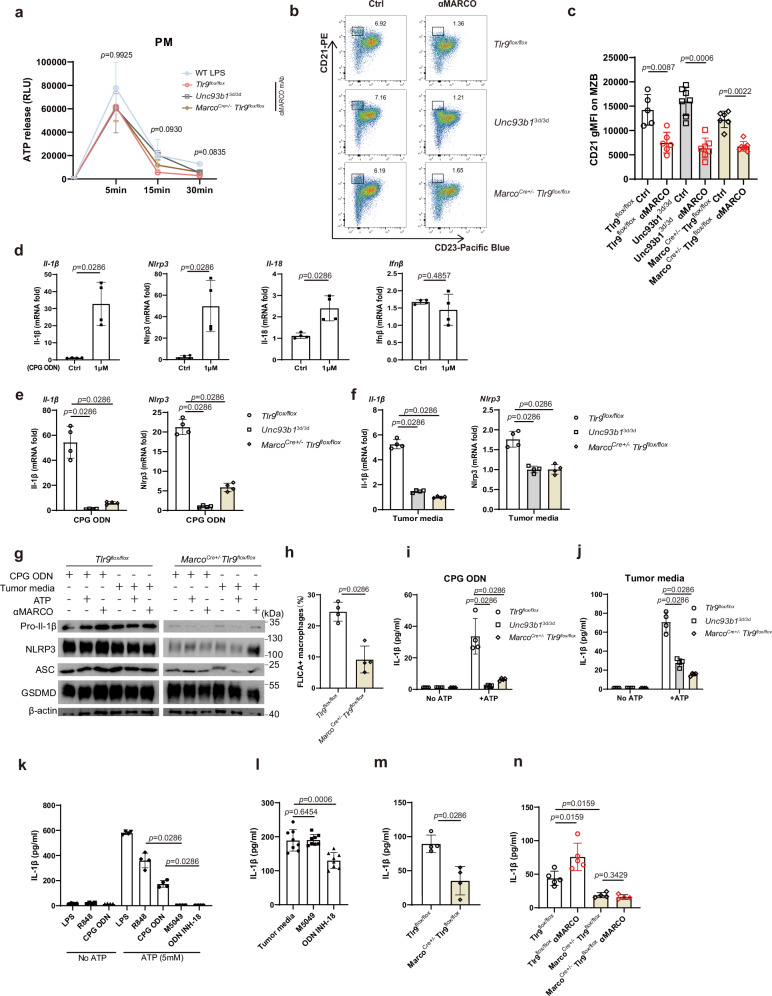
Anti-MARCO promotes inflammasome activation and IL-1*β* secretion in a TLR9 dependent manner. **a** ATP release upon αMARCO antibody stimulation of peritoneal macrophages (PM). Data have been subtracted from the baseline level. *n* = 3 mice per group. **b** Spleens from *Tlr9*^*flox/flox*^, *Unc93b1*^3d/3d^ and *Marco*^*Cre+/−*^ *Tlr9*^*flox/flox*^ mice injected (i.v.) with control or αMARCO (50 μg/mouse) for 24 h. Dot plots showing the percentage of CD21^+^ cells on marginal zone B cell (MZB). **c** The expression of CD21 on MZB gated using CD1d. *Tlr9*^*flox/flox*^ Ctrl *n* = 5, *Tlr9*^*flox/flox*^ αMARCO *n* = 6, *Unc93b1*^3d/3d^ Ctrl *n* = 7, *Unc93b1*^3d/3d^ αMARCO *n* = 7, *Marco*^*Cre+/−*^ *Tlr9*^*flox/flox*^ Ctrl *n* = 6, *Marco*^*Cre+/−*^ *Tlr9*^*flox/flox*^ αMARCO *n* = 6. **d** Relative expression of *Il-1β*, *Nlrp3*, *Il-18*, and *Ifnβ* on PMs from WT mice stimulated with or without CPG ODN (TLR9 agonist) (1 μM) for 12 h. *n* = 4 mice per group. **e** Relative expression of *Il-1β* and *Nlrp3* on PMs stimulated with CPG ODN (1 μM) for 12 h. *n* = 4 mice per group. **f** Relative expression of *Il-1β* and *Nlrp3* on PMs stimulated with tumor-conditioned media for 12 h. **g** Western blot analysis of inflammasome-related proteins in PMs isolated from *Tlr9*^*flox/flox*^ and *Marco*^*Cre+/−*^ *Tlr9*^*flox/flox*^ mice. **h** The percent of FLICA+ macrophage in PMs. *n* = 4 mice per group. Data have been subtracted from the baseline level. **i** IL-1β levels detected in the supernatant of PMs stimulated with CPG ODN (1 μM) for 12 h following with ATP (5 mM). *n* = 4 mice per group. **j** IL-1β levels detected in the supernatant of PMs stimulated with tumor-conditioned media for 12 h following with ATP (5 mM). *n* = 4 mice per group. **k** IL-1β levels detected in the supernatant of WT PMs stimulated with indicate reagents for 12 h following with ATP (5 mM). *n* = 4 mice per group. **l** IL-1β levels detected in the supernatant of WT PMs pretreated with M5049 (1 μM) or ODN INH-18 (1 μM) for 6 h followed with tumor-conditioned media for 6 h and stimulated with ATP (5 mM). *n* = 8 mice per group. **m** IL-1β levels detected in the supernatant of B16-F10 tumor-conditioned media after 24 h cultured. *n* = 4 mice per group. **n** IL-1β levels detected in the supernatant of EO771 tumor-conditioned media after 24 h cultured. *Tlr9*^*flox/flox*^ *n* = 5, *Marco*^*Cre+/−*^ *Tlr9*^*flox/flox*^ *n* = 4. Representative graph shows one representative experiment out of two (**a**–**c**, **g–n**) and three (**d**–**f**) independent repeats. Dots represent biological replicates (**a**, **c**–**f**, **h–n**). Bar plots show mean ± s.e.m. *P* values were calculated by two-side Mann–Whitney *U* test.

Next, we proceeded to investigate the underlying mechanism by which TLR9 was involved in αMARCO mediated immunotherapy. We have previously reported that after activation using αMARCO antibodies, macrophages release extracellular ATP^24^. We also showed that ATP release from MARCO expressing marginal zone macrophages (MZM) in vivo makes marginal zone B cells (MZB) shed their CD21 receptors^24^. To assess whether endosomal TLRs, and in particular TLR9, influence this mechanism, we examined macrophages from littermate controls, *Unc93b1*^3d/3d^, and *Marco*^*Cre+/−*^ *Tlr9*^*flox/flox*^ mice. The ATP release assay revealed that all the macrophages exhibited similar levels of ATP release following αMARCO stimulation (Fig. 8a). In vivo, we also found that the ATP-driven loss of CD21 on MZBs was similar in littermate controls, *Unc93b1*^3d/3d^ and *Marco*^*Cre+/−*^ *Tlr9*^*flox/flox*^ mice after αMARCO injection, while the CD1d expression on MZBs was unaffected (Fig. 8b, c and Supplementary Fig. 8a, b). These data suggested that the requirement for TLR9 was connected to a secondary step in the macrophage response to αMARCO. Even though persistent inflammation can drive tumor progression, therapeutic agent-induced acute inflammation often enhances antitumor immunity. Notably, certain cytotoxic chemotherapies depend on host pattern recognition receptors (PRRs), such as TLRs and inflammasomes, for their full therapeutic efficacy^25,26^. In connection with ATP, a damage-associated molecular pattern (DAMP) activates macrophages via the P2X7 channel, triggering NLRP3 inflammasome activation and subsequent IL-1β processing and secretion^27^. We hypothesized that TLR9 was needed in MARCO-expressing macrophages to prepare them for a response to ATP by regulating transcription of the inflammasome components. To investigate this possibility, we stimulated macrophages with TLR9 agonists and detected significantly elevated expression of inflammasome-related genes (Fig. 8d, e and Supplementary Fig. 8c, d). This was also true using tumor-conditioned media from the B16-F10 tumors, showing that TLR9 was needed in the TME to make TAMs ready to respond to ATP (Fig. 8f). To further assess inflammasome activation, we examined key components of the inflammasome using Western blot. We observed a reduction of pro-IL-1β, NLRP3 and ASC in the conditionally targeted macrophages when stimulated with TLR9 agonist or tumor-conditioned media, but not with TLR4 agonist (Fig. 8g and Supplementary Fig. 8e). This was also true regardless of stimuli. We next evaluated inflammasome activation in peritoneal macrophages and found that activation of Caspase-1 was reduced in macrophages from the conditionally targeted mice (Fig. 8h). We also found increased IL-1β secretion in WT macrophages upon ATP induced activation after preincubation with TLR9 agonists or tumor-conditioned media. However, consistent with reduced expression of the inflammasome machinery, this response was markedly impaired in macrophages lacking endosomal TLR signaling (*Unc93b1*^3d/3d^) or in MARCO^+^ macrophages with TLR9-specific deletion (*Marco*^*Cre+/−*^ *Tlr9*^*flox/flox*^) (Fig. 8i, j). This was specific to TLR9 since when we pre-incubated with LPS to condition macrophages from *Unc93b1*^3d/3d^ and *Marco*^*Cre+/−*^ *Tlr9*^*flox/flox*^ mice they had the capacity to secrete IL-1β following ATP stimulation (Supplementary Fig. 8f). In addition, blocking TLR9 but not TLR7 reduced the IL-1β production induced by the tumor-conditioned media, showing that TLR9 ligands are responsible for driving this response (Fig. 8k, l). These findings suggested that inflammasome activation in the TME is specific to the TLR9-ATP axis coupled to αMARCO antibody treatment. To validate these findings in vivo, tumor-conditioned media were harvested from littermate controls and *Marco*^*Cre+/−*^ *Tlr9*^*flox/flox*^ tumor-bearing mice. Baseline levels of IL-1β and IFN-γ secretion were significantly lower in *Marco*^*Cre+/−*^ *Tlr9*^*flox/flox*^ tumors compared to in controls (Fig. 8m and Supplementary Fig. 8g). Moreover, the increased production of IL-1β induced by αMARCO in vivo was also dependent on TLR9 (Fig. 8n). Together, these data show that αMARCO promotes inflammasome activation and IL-1β secretion through a TLR9-dependent mechanism. This includes upregulation of proteins of the inflammasome machinery which are critical for modulating macrophage repolarization and function and promoting anti-tumor immunity.

## Discussion

Macrophages are first-line responders in the immune system. In this role, they employ gene expression programs driven by environmental cues to prepare them for effector functions in the tissue they reside in. This applies to TAMs as well, where the microanatomical niches that exist in the tumor tissue will drive an array of polarization states that affect immunotherapeutic treatment outcomes^28^. This also relates to the local metabolic regulation of TAMs that directly impacts their function and mode of regulation^29^. In contrast to T cells, TAMs are consistently present in the tumor, regardless of whether it is “hot” or “cold” with respect to lymphocyte infiltration. In recent years, efforts have been made to harmonize the TAM data from a multitude of studies using single-cell analysis of tumors across different cancer types^2,30^. The analysis conducted so far suggests that specific clusters of polarization states exist across cancer types, with some exhibiting expression of MARCO alongside inflammasome components^31^. An important finding in this study is that endosomal TLRs are needed for the response to immunotherapy using αMARCO antibodies. However, there was no difference in tumor growth in the control groups even though there were clear changes in the overall gene expression of the TAMs and their numbers. When investigating the TAMs in bulk from WT or mice lacking functional endosomal TLRs, we found mixture of polarization towards both pro and anti-inflammatory states that could partly explain this. Thus, we went on to investigate the response in higher resolution, and our scRNA-seq data show six major myeloid populations, and three of these clearly respond to αMARCO treatment by changes in gene expression. Of these, the SPP1+ TAMs have been shown to also express MARCO in human tumors^32^. In addition, MARCO-expressing TAMs are coupled to poor prognosis in several cancer types, including breast cancer^33^, pancreatic cancer^34^, prostate cancer^35^, glioblastoma^36^. Moreover, MARCO expression has been suggested as a relevant marker for clinical evaluation of tumor grades^33^. Targeting TAMs for immunotherapy in humans has been attempted using two main approaches to date: **(1)** Suppressing TAM recruitment or survival primarily by blockade or modification of CSF1-receptor function, or **(2)** by reprogramming approaches using various drugs, including TLR agonists^37^. The findings here show that TAMs require endosomal TLRs for efficient anti-tumor responses using αMARCO and αPD-L1 antibodies in two tumor models. This requirement was fulfilled without injections of ligands for endosomal TLRs, which instead were provided endogenously by the TME, presumably from dying tumor cells and/or cellular stress responses. It is known that necrotic and apoptotic cells can activate TLR9 through release of genomic or mitochondrial DNA^38,39^. It is also likely that these ligands are more stable in the TME than ligands for TLR7 as these will be subjected to potent RNAse activity. In support of this, the tumor-conditioned media from untreated tumors was sufficient to upregulate components of the inflammasome in a TLR9-dependent manner in MARCO-expressing macrophages. In addition, combinatory treatment using TLR9 ligands and αMARCO showed increased treatment efficacy. These data collectively provide evidence for that for TAMs to be targetable using these antibodies, they need to be “pre-polarized” within the tumor and that this can also be achieved using exogenous ligands. This aligns with previous findings in which exogenous TLR9 ligands were necessary for generating effective tumoricidal macrophage phenotypes and overcoming the do-not-eat-me signals of tumor cells^40^. In addition, direct injection of poly(I:C) + R848 in the tumor altered the balance between pro- and anti-tumor TAMs^41^. In humans, TLR9 agonists (IMO-2055 and SD-101) have been evaluated in clinical trials and were well tolerated, but it is still too early to evaluate therapeutic efficacy^42–44^. Importantly, we also find that MARCO is co-expressed with TLR9 in the TME of breast cancer patients. It will be of interest to evaluate this combination of markers in future clinical evaluation of tumors before and after treatment. We are currently developing an anti-human MARCO antibody (anti-hMARCO), and a combination treatment with TLR9 agonists and anti-hMARCO may be a plausible approach for efficient TAM targeting. Also, the data herein showing TLR9 expression in MARCO-positive macrophages in all types of human breast cancer is encouraging for moving this treatment forward to clinical application. It is evident that new avenues for immunotherapy must be explored for many cancers. In this respect, targeting macrophages within the tumor represents an appealing strategy. Here, we report that the MARCO-expressing TAM subpopulation is indeed targetable and provides a mechanism of action whereby TLR9 pre-polarization/priming is essential for achieving beneficial treatment effects. This implies that tumors containing TAMs can be cold, and rather than differences in numbers of macrophages, the treatment effect is related to their polarization state. This is also true for αPD-L1 treatment, which relies on endosomal TLRs. As PD-L1 is expressed by many cell types in the TME, the data suggest that targeting TAMs is crucial for efficacy and treatment outcomes. Further studies will determine the exact mechanistic connection between endosomal TLRs and PD-L1 expression, including the effect on other cell types. The effect of TLR7 and TLR3 in the TME is worth pursuing, as we found that PD-L1 upregulation was connected to these receptors. Other venues for exploration are investigations into immunometabolism, as targeting TLR9 is known to modulate metabolism and thereby influences the polarization and function of TAMs^29^. Given the key role of macrophages in tumor progression, including generation of metastases, targeting the MARCO-expressing subpopulation of TAMs should improve immunotherapeutic treatment outcomes.

## Methods

### Mice

C57BL/6J (BL/6J) mice were purchased from Charles River Laboratories (Germany) under an agreement with The Jackson Laboratory (stock# 000664). *Unc93b1*^3d/3d^ mice were a gift from Dr. Bruce Beutler^10^. *MARCO-Cre* mice were made by Tak W. Mak (Toronto University)^45^. C57BL/6J-Tlr9^em1Ldm^/J (*Tlr9*^*flox/flox*^) mice (stock #:034448) and B6.Cg-Gt(ROSA)26Sor^tm14(CAG-tdTomato)Hze^/J (Rosa-tdTomato) reporter mice (stock #:007914) were obtained from The Jackson Laboratory (USA). All animals were randomly co-housed in groups under specified pathogen-free conditions at the Comparative Medicine Animal Facility at Karolinska Institutet and had unlimited access to standard laboratory chow and water. Mice were maintained under a light-dark cycle of 12 h, with a temperature of 20–25 °C and humidity of 50–60%. We confirm that the research performed in this study complies with all relevant ethical regulations. All animal experiments conducted in this study were approved by the Stockholm’s Animal Experimental Ethical Committee (Dnr 4240-2021).

### Tumor models

B16-F10 mouse melanoma cell line (ATCC, Cat#CRL-6475) was maintained in DMEM supplemented with 10% fetal bovine serum (FBS), 1% penicillin-streptomycin. EO771 mouse breast cancer cell line (CH3 BioSystems, Cat#94A001) was cultured in RPMI-1640 medium supplemented with 10% FBS, 1% penicillin-streptomycin, and 1% L-Glutamine. All cells used for the individual repeats of the experiment originate from the same early passage stock and are cultured for two passages before in vivo administration. 8–12 week-old male (B16-F10 model) or 8–14 week-old female (EO771 model) mice were randomly assigned to the experimental groups. Male mice were subcutaneously inoculated with 5 × 10^5^ B16-F10 tumor cells in 100 μL PBS on day 0 in the right flank. Female mice were orthotopically inoculated with 2 × 10^5^ EO771 tumor cells in 100 μL PBS on day 0 in the right mammary fat pad. Tumor volume (0.5236 × length × width × width) was measured every other day using a caliper and monitoring frequency was increased to daily once the tumor volume reached 0.8 cm^3^. No animal bore a tumor exceeding the maximum permitted size during the experiments. Animals were randomized based on tumor volume prior to treatment, and experimental measurements were conducted in a blinded manner. On day 12 (or 21 for EO771 model) of the experimental setup, tumor-bearing animals were euthanized via CO_2_ inhalation, and tumors were collected for further analysis.

### Immunotherapy

All the antibody treatment was performed by intravenous (i.v.) injection starting on the third day after tumor inoculation. For αMARCO treatment, the rat anti-MARCO IgG1 (Clone: ED31) was obtained from Mabtech. Mice were treated with 100 μg of anti-MARCO antibody (rat IgG1, clone ED31) or IgG1 isotype control antibody (BioXCell, rat IgG1, clone TNP6A7) on day 3, 6, 9 (and 12 for EO771 model). For the experiments of CD21 downregulation on MZB, mice were injected with 50 μg of anti-MARCO antibody or IgG1 control antibody for 24 h. For the anti-PD-1 antibody or anti-PD-L1 antibody treatments, mice were injected with 180 μg anti-PD1 (BioXCell, rat IgG2a, clone RMP1–14), or 75 μg anti-PD-L1 antibody (BioXCell, rat IgG2b, clone 10 F.9G2) on day 3, 6, 9 (and 12 for EO771 model). For combo therapy, mice were injected with 20 μg CPG ODN (Invivogen, #tlrl-2395) together with 100 μg of anti-MARCO antibody (rat IgG1, clone ED31) on day 3, 6, 9 (and 12 for EO771 model).

### Tumor and tissue preparation

Tumors were harvested at the indicated time point in cold DMEM digest medium (DMEM culture medium supplemented with 2% FBS, 2 mM L-glutamine, 10 mM sodium pyruvate, 1% non-essential AAs, 2% essential AAs, and 15 mM Hepes). Tumors were finely cut into pieces using surgical scissors and further enzymatically dissociated through the addition of 100 μg/ml DNAse (Roche), 150 μg/ml Liberase TL (Roche) for 30 min at 37 °C water baths. After incubation, cells were passed through 100μm filter strainer and washed thoroughly with 5 ml MACS Buffer (1 × PBS supplemented with 0.5% BSA, 2 mM EDTA, 2mM L-glutamine, 10 mM Sodium Pyruvate, 1% Non-essential AAs, 2% Essential AAs, and 25 mM D-Glucose). Later, cells were lysed with ACK Lysis Buffer (Gibco) to get rid of red blood cells (RBC). Tumor cells were either processed to obtain tumor-conditional media or CD45 selection. For tumor-conditioned media preparation, cells were seeded at a concentration of 5 × 10^6^ cells/ml and incubated for 48 h at 37 °C. Collected supernatant was filtered through a 0.22 μm filter and store at −20 °C until further use. For positive selection by magnetic cell isolation, cells were labeled with CD45 MicroBeads (Miltenyi Biotec) followed with the kit protocol. Purified CD45 cells were resuspended in PBS and ready for further staining. For spleen sample preparation, the spleen was dissected out, mushed through a 70 μm strainer, and followed by lysis of RBC before staining.

### Flow cytometry

Single-cell suspensions of tumors were prepared according to previous descriptions. Non-specific labeling was blocked with anti-CD16/32 (FC Block; Biolegend) before specific labeling. Viability was assessed with LIVE/DEAD Fixable Aqua (Invitrogen) in PBS for 15 min at room temperature (RT) before surface staining. Surface staining was performed in MACS buffer for 30 min at 4 °C. Subsequently, cells were fixed and permeabilized for intracellular staining using the FoxP3/Transcription Factor Staining Buffer Set (eBioscience) according to manufacturer’s protocol. Samples were analyzed using a BD LSR Fortessa ×-20 cytometer and analyzed with FlowJo software (v10.8.1). For FACS sorting, after tumor dissociation and CD45 positive selection, Live CD45^+^ cells or Live CD45^+^ CD11b^+^ F4/80^+^ Gr-1^−^ cells were sorted using a BD FACS Aria fusion cell sorter for RNA-sequencing or co-culturing with T cells. A full list of the antibodies used in the study and dilutions is provided in Supplementary Table 1. For gating strategy refer to Supplementary Fig. 9).

### Generation and processing of bulk RNA-sequencing data

TAMs (live, CD45^+^CD11b^+^F4/80^+^Gr-1^−^ cells) were isolated as indicated and directly collected in 1 ml of TRIzol (Invitrogen) before storage at −80 °C. Total RNA was extracted and isolated following TRIzol Reagent User Guide. Messenger RNA was purified from total RNA using poly-T oligo-attached magnetic beads. RNA integrity was assessed using the Bioanalyzer 2100 system (Agilent Technologies, CA, USA). After fragmentation, the first strand cDNA was synthesized using random hexamer primers, followed by the second strand cDNA synthesis. The library was ready after end repair, A-tailing, adapter ligation, size selection, amplification, and purification. The library was checked with Qubit and real-time PCR for quantification and bioanalyzer for size distribution detection. After library quality control, different libraries were pooled based on the effective concentration and targeted data amount, then subjected to Illumina sequencing by using the NovaSeq 6000 platform. As each sequencing cluster extends its complementary strand, the addition of each fluorescently labeled dNTP releases a corresponding fluorescence signal. The sequencer captures these fluorescence signals and converts them into sequencing peaks through computer software, thereby obtaining the sequence information of the target fragment.

### Bulk RNA-sequencing data analysis

Raw data of fastq format were first processed through fastp software. In this step, clean data was obtained by removing reads containing adapter, reads containing ploy-N and low-quality reads from raw data. At the same time, Q20, Q30 and GC content the clean data were calculated. All the downstream analyses were based on the clean data with high quality. Reference genome and gene model annotation files were downloaded from genome website directly. Index of the reference genome was built using Hisat2 v2.0.5, and paired-end clean 1 reads were aligned to the reference genome using Hisat2 v2.0.5. We selected Hisat2 as the mapping tool for that Hisat2 can generate a database of splice junctions based on the gene model annotation file and thus a better mapping result than other non-splice mapping tools. FeatureCounts v1.5.0 was used to count the reads numbers mapped to each gene, and then TPM of each gene was calculated based on the length of the gene and reads count mapped to this gene. Differential expression analysis for two conditions was performed using the DESeq2 R package (1.40.2). DESeq2 provides statistical programs for determining differential expression in digital gene expression data using models based on negative binomial distribution. The resulting *P*-value is adjusted using the Benjamini and Hochberg’s methods to control the error discovery rate. The corrected *P*-value ≤ 0.05 & |log2(foldchange)| ≥ 0.5 was set as the threshold of significant differential expression. GO and KEGG enrichment analysis of differentially expressed genes was implemented by the clusterProfiler R package (4.10.1), in which gene length bias was corrected. GO and KEGG terms with corrected *P*-value less than 0.05 were considered significantly enriched by differential expressed genes.

### Generation and library preparation of single-cell RNA-sequencing data

To minimize the impact of individual variation on sequencing outcomes, 3 to 4 tumor samples per experimental group were pooled into a single biological replicate prior to downstream processing. Single-cell suspensions generated from the EO771 tumors were stained with viability dye and anti-CD45 antibodies. Live CD45^+^ cells were subsequently isolated as previously indicated. Immediately following sorting, the collected cells were manually counted under the microscope, and the cell concentration was adjusted to 1200–1400 cells/μl. Approximately 9000–11,000 cells per well were loaded onto a GEM-X OCM 3′ Gene Chip v4 4-plex (10× Genomics, PN-1000747), which was then immediately run on a Chromium X (10× Genomics) to generate single-cell GEMs (Gel Beads-In-Emulsions). Single-cell RNA-seq libraries were constructed using the GEM-X Universal 3′ Gene Expression v4 4-plex kit (10× Genomics, PN-1000779) according to the manufacturer’s instructions. Sequencing was performed on a DNBSEQ-G400 sequencer (MGI Tech) with the following run parameters: Read 1: 28 bp; Read 2: 150 bp; i7 index: 10 bp; i5 index: 10 bp. Following sequencing, raw reads were demultiplexed using SplitBarcode (v1.0.1). The demultiplexed data were then processed using the Cell Ranger pipeline (v9.0.1, 10× Genomics) for alignment, barcode assignment, and UMI counting against the pre-compiled mouse reference transcriptome (refdata-gex-mm10-2020-A, 10× Genomics). The resulting raw gene expression count matrices were used for subsequent downstream analysis.

### Histology and immunofluorescence

Tumors from mice were isolated and directly fixed in 4% paraformaldehyde (PFA) overnight at 4 °C followed by 15 and 30% sucrose to dehydrate the tissue and embed with OCT medium (Bio-Optica), while spleens were cryopreserved in OCT medium directly. Embedded samples were cut into 10-μm-thick sections to Superfrost Ultra Plus slides (Thermo Scientific) using a cryostat microtome (NX70-Thermo cryostat). Slides were stored at −20 °C for short-term or −80 °C for long-term storage.

Tumors from human patients were paraffin-embedded and sectioned into 5-μm-thick slices. Heated paraffin sections were dewaxed in xylene, followed by ethanol series. After blocking endogenous peroxidase activity with 3% hydrogen peroxide for 20 min, slides were boiled in 10 mM citrate buffer (pH 6.0) for 20 min to perform antigen retrieval. Slides were ready for the following staining steps. Before staining, mouse sections were blocked with 5% goat serum (Dako Denmark A/S) and Fc Block in PBS for 30 min at RT. For human sections, blocking buffer also add 0.05% Triton ×-100 for cell permeabilization. Mouse tumor slides were incubated with anti-MARCO antibody conjugated to AF488 (Life Technologies), anti-F4/80 eFluor 570 (Invitrogen), anti-CD31 AF647 (Biolegend); while spleen slides were incubated with anti-MARCO AF488 (as previously); anti-F4/80 eFluor 570 (as previously); anti-B220 AF647 (BD Biosciences). Human breast carcinoma sections were incubated with primary antibody at 4 °C overnight followed by secondary antibody for 1 h at RT. Samples were stained with primary antibody mouse-anti-human TLR9 (Abcam), and secondary goat-anti-mouse IgG (H + L) AF647 antibody (Invitrogen). After washing out, slides were incubated with anti-human MARCO antibody conjugated to AF555 (Life Technologies); anti-human CD68 AF488 (Santa Cruz Biotechnology) at 4 °C overnight. The nuclei were counterstained with DAPI in PBS for 5 min (dilution 1:3000) in the dark and mounted with ProLong Diamond Antifade Mountant (Thermo Scientific). All sections were imaged using a confocal microscope (Leica LSM 980) and images were analyzed with ImageJ (Fiji) software. A full list of the antibodies used in the study and dilutions is provided in Supplementary Table 1.

### Macrophage isolation and polarization

For BMDMs, bone marrow was isolated from femur and tibia and subsequently lysed with ACK Lysis Buffer (Gibco) for 3 min at RT. Murine monocytes were cultured in BMDM culture medium (DMEM supplemented with 20% Cytiva HyClone FBS, 1% GibcoTM P/S and 10 ng/ml M-CSF) for 8 days to allow differentiation of monocytes to macrophages. Half of the cell culture medium was replaced with fresh BMDM culture medium on day 3, and the entire volume of cell culture medium was replaced with fresh BMDM medium on day 6. BMDMs were then polarized toward either pro-inflammatory (20 ng/ml IFN-γ (Peprotech) and 10 ng/ml LPS (Invivogen)) phenotype or immune suppressive (20 ng/ml IL-4 (Peprotech) and 20 ng/ml IL-10 (Peprotech)) phenotype for 48 h. To generate in vitro-induced TAMs, unpolarized BMDMs were cultured for 48 h with tumor-conditioned media mixed with 10% FBS DMEM medium at a ratio of 1:1, and cells were cultured for 48 before further functional analysis. If stated, macrophages were treated with αMARCO antibody (10 μg/ml) for 10 h. Following this incubation, BMDMs were either evaluated for their gene and surfacer markers expression or further co-cultured with T cell. BMDMs were detached from cell culture plates by using PBS with 10 mM EDTA with 20 min ice bath. Peritoneal macrophages (PM) were extracted from the peritoneal cavity of 8–12 weeks mice. The cells were cultured for 4–6 h in DMEM (10% Cytiva HyClone FBS, 1% Gibco P/S) to allow all the macrophages in the cell suspension to attach to the bottom of the cell culture plate. All cells were cultured in a humidified environment at 37 °C and 5% CO_2_.

### Co-culture experiments

For co-culture experiments, murine T cells were isolated from spleens via negative selection (Mouse pan T cell isolation kit, Miltenyi) and cultured in RPMI supplemented with 10% FBS, 1% PenStrep and 0.1% β-mercaptoethanol followed with CFSE labeling (Invitrogen) and stimulated with CD3/CD28 dynabeads (ThermoFisher) activation. Murine BMDMs or TAMs sorted from the tumors were co-cultured with splenic T cells for 2 days at an effector to target (E:T) ratio of 1:8, and T cell proliferation and IFN-γ production were measured.

### Macrophage activation assay

To assess the contribution of TLR ligands for macrophage activation, BMDMs or PMs were stimulated with 1 μg/ml TLR3 agonist Poly I:C (Invivogen, #tlrl-pic), 1 μg/ml TLR7 agonist R848 (Invivogen, #trlr-r848) or 1 μM TLR9 agonist CPG ODN (Invivogen, #tlrl-2395) for 12 h after which they were analyzed by flow cytometry or for RNA extraction.

### Analyses of cell culture supernatant by ELISA

To assess the IFN-γ production by T cells, 96-wells flat-bottom ELISA plate was coated with 1 μg/ml IFN-γ capture antibody (AN18) at 4 °C overnight. After blocking with 1% BSA for 1 h, cell supernatants or standard substance were incubated at RT for 2 h or at 4 °C overnight. After washing, 2 μg/ml IFN-γ biotin-labeled detection antibody (R4-6A2-biotin) was added and incubated at RT for 1 h, followed by another one-hour incubation with streptavidin-HRP. Mouse PMs and tumor-conditional media were harvested as indicated. For quantification of IL-1β, mouse PMs were stimulated for 12 h with either 100 ng/ml LPS, 1 μg/ml R848, 1 μM CPG ODN or tumor-conditional media and ATP (5 mM) was added for the last 30 min for stimulation. To assess the contribution of TLR for inflammasome activation, mouse PMs were stimulated with either 1 μM TLR7 antagonist M5049 (Invivogen, #inh-m5049) or 1 μM TLR9 antagonist ODN INH-18 (Invivogen, #tlrl-inh18) for 6 h, followed by 6 h tumor-conditional media stimulation and ATP (5 mM) was added for the last 30 min for stimulation. Supernatants were collected and centrifuged to remove cellular debris. IL-1β (ELISA Flex: Mouse IL‑1β (HRP), Mabtech) was measured following manufacturer’s instructions. Absorbance was measured using a Microplate Spectrophotometer (BioTek).

### ATP-release assays

For ATP release assay, PMs were seeded in 24-well plates in serum-free medium and nonadherent cells were removed. The assay was performed at room temperature and cells were stimulated with either 10 μg/ml αMARCO antibody, 10 μg/ml isotype control, or 10 ng/ml LPS. Extracellular ATP release was measured with a luminometer using the ENLITEN ATP Assay System Kit (Promega). Luminescence was measured on a luminometer (TECAN Infinite).

### Caspase-1 FLICA analysis

Activated Caspase-1 was detected using the FAM-FLICA Caspase-1 Assay Kit (ImmunoChemistry Technologies) according to the manufacturer’s instructions. In brief, PMs were stimulated with tumor-conditioned media for 12 h and followed with ATP for the last 30 min as previously indicated. After ATP stimulation, immediately washed away the media and incubated the PMs with FLICA for 30 min at 37 °C in dark. Later PMs were washed and detached from cell culture plates by using PBS with 10 mM EDTA with 20 min ice bath. Cells were stained with LIVE/DEAD Fixable Aqua followed by fluorochrome-conjugated antibody cocktail for cell surface markers as previously described and detected by flow cytometry.

### Western blotting

To assess inflammasome activation, whole-cell lysates were prepared by lysing cells in RIPA buffer (Sigma-Aldrich) supplemented with protease and phosphatase inhibitors (Thermo Fisher Scientific) on ice for 30 min. The lysates were mixed with LDS sample buffer (Life Technologies) and incubated at 100 °C for 5 min. Samples were separated by sodium dodecyl sulfate-polyacrylamide gel electrophoresis (SDS-PAGE) using Bolt 4–12% Bis-Tris Plus gels (Invitrogen) and transferred to PVDF membranes (Bio-Rad) using Bolt Transfer Buffer (Invitrogen). Membranes were blocked for 1 h at room temperature (RT) with 5% nonfat milk in Tris-buffered saline containing 0.05% Tween-20 (TBST). Following blocking, membranes were incubated overnight at 4 °C with primary antibodies diluted in TBS-BSA-Tween. The membranes were then washed, incubated with HRP-conjugated secondary antibodies for 1 h at RT, and washed again before analysis using a chemiluminescent substrate kit (Thermo Fisher Scientific). The following rabbit mAbs were used: anti-NLRP3 (D4D8T, 1:1000), anti-ASC (D2W8U, 1:1000), and anti-Pro-IL-1β (D6D6T, 1:1000) from Cell Signaling Technology (CST); anti-GSDMD (EPR20859, 1:1000) and anti-β-actin (mAbcam 8226, 1:5000) from Abcam. A secondary anti-rabbit HRP-linked antibody (#7074, 1:2000, CST) was used for detection. A full list of the antibodies used in the study and dilutions is provided in Supplementary Table 1.

### Quantitative real-time PCR

Total RNA was isolated from BMDMs or PMs using RNeasy Kit (QIAGEN) and quantified by Nanodrop (ThermoFisher). RNA was reverse transcribed with iScript cDNA synthesis kit (Bio-Rad). cDNA (300 ng) was mixed with 10 nM primers and iQ SYBR Green Supermix (Bio-Rad) and analyzed on CFX Touch Real-Time PCR Detection system (Bio-Rad). All primers used in this study are included in Supplementary Table 2. Data were analyzed with Bio-Rad software.

### TCGA melanoma and breast cancer data analysis

Gene expression data of melanoma in TCGA-SKCM (17 datasets, *n* = 481) and breast cancer in TCGA-BRCA (24 datasets, *n* = 1247) project were obtained from the official website of the project (http://cancergenome.nih.gov) and UCSC Xena datasets (https://xenabrowser.net/).

### Patient samples

Ten patient samples have been obtained as part of routine diagnosis. Histological evaluations of patient tumors were evaluated by pathologists for diagnostic purposes: tumor characteristics, including hormone receptors, HER2, and Ki67 expression, and were classified as Luminal A, Luminal B, HER2, and triple-negative breast cancer (TNBC). Samples from Luminal A, Luminal B, and HER2 were all from treatment-naïve patients. Samples from two TNBC patients received neoadjuvant chemotherapy, but both stopped halfway and underwent surgery. All patient samples were fully anonymized with only the treatment status disclosed and all recruited patients provided written informed consent. Human breast cancer samples were obtained by Dr. Johan Hartman from the Department of Clinical Pathology and Cancer Diagnostics at Karolinska University Hospital, Stockholm, Sweden. Experimental procedures and protocols of the study were previously approved by the regional ethics review board (Etikprövningsnämnden) in Stockholm.

### Single-cell RNA-sequencing data processing and analysis

Raw single-cell RNA-sequencing data were processed as previously described. All downstream analyses were performed in the R environment (v4.2.0) using Seurat (v4.4.0) and ggplot2 (v3.5.2). Initial cell quality control was conducted by filtering out low-quality cells with nFeature_RNA < 200, nCount_RNA < 200, and mitochondrial content (percent.mt) > 30%. Subsequently, to minimize technical noise and focus on biologically informative protein-coding transcripts for cell type identification, specifically identified non-coding and interfering genes were physically removed from the dataset based on the Mouse genome (mm10). To ensure robust signal-to-noise ratio, genes expressed in fewer than 0.5% of the total cell population or with a total count of less than 3 were excluded. The filtered data were normalized and variance-stabilized using SCTransform workflow. Dimensionality reduction was performed via Principal Component Analysis (PCA), and the first 30 principal components were used for two-dimensional Uniform Manifold Approximation and Projection (UMAP) visualization. Cluster-specific genes were identified using the FindAllMarkers function with option only.pos = TRUE and logfc.threshold = 0.5, setting a cut-off of adjusted *p*-value < 0.05. Cell types were manually annotated based on the expression of canonical marker genes and prior biological knowledge.

To characterize the polarization states of TAMs, polarization scores were calculated using single-cell unified polarization assessment (Scupa) based on the predefined macrophage states (Mac.a to Mac.e) according to the Immune Dictionary^16^ Pathway activities were inferred using PROGENy (v 1.20.0)^46^ Cell–cell communication analysis was performed using CellChat (v 1.6.1) to explore interactions among macrophages, monocytes, and major lymphoid cell populations. The custom code used for this analysis are available in the GitHub repository (see “Code Availability”).

### Quantification and statistical analysis

Data was analyzed by a two-side Mann–Whitney *U* test for two-group comparisons or an ordinary one-way ANOVA followed by Tukey’s post hoc test for multiple-group comparisons as appropriate. All statistical tests were performed using the GraphPad Prism 8 software. All quantitative data are presented as the mean ± SEM unless stated otherwise. The exact *p* values are presented in the figures to indicate statistical significance. Data are representative of at least two independent experiments.

### Reporting summary

Further information on research design is available in the Nature Portfolio Reporting Summary linked to this article.

## Supplementary information

Supplementary Information

Reporting Summary

Transparent Peer Review file

## Source data

Source Data

## Data Availability

Single-cell and bulk RNA-seq data have been deposited at NCBI GEO data repository under accession number GSE303908 [https://www.ncbi.nlm.nih.gov/geo/query/acc.cgi?acc=GSE303908] and NCBI SRA data repository under accession number SRP604259 [https://www.ncbi.nlm.nih.gov/sra/?term=SRP604259]. Source data are provided with this paper. All data are included in the supplementary information or available from the authors, as are unique reagents used in this article. The raw numbers for charts and graphs are available in the Source Data file whenever possible. Source data are provided with this paper.

## Code availability

The codes for the bulk RNA-seq and scRNA-seq data analysis is available at https://github.com/yuepan96/RNA-Seq.
