# Supplementary material for: Cancer immunotherapy targeting murine myeloid cells requires endosomal pattern recognition
Source: Nat Commun. 2026 Jul 27;17:7410. doi: 10.1038/s41467-026-75543-2 (PMC13408345; doi:10.1038/s41467-026-75543-2)
Supplement: Supplementary file 1 — Supplementary Information [file 41467_2026_75543_MOESM1_ESM.pdf]

Supplementary Information

Cancer immunotherapy targeting MARCO on myeloid cells requires endosomal pattern recognition in mice.

Yueyun Pan[1], Shengduo Pei[1], Heng Liang[1], Kajsa Westberg[1], Lars Nitschke[2], Xinsong Chen[3], Yinda Yu[4], Ning He[4], Qirong Lin[5], Li Lei[5], Anton Gisterå[4], Johan Hartman[3], Jeffrey V. Ravetch[6], Tak W. Mak[7], Tracy L. McGaha[7] and Mikael C.I. Karlsson[1*]

[1] Department of Microbiology, Tumor and Cell Biology, Karolinska Institutet, Solna Campus, Stockholm, Sweden

[2] Division of Genetics, University of Erlangen, Germany

[3] Department of Oncology-Pathology, Karolinska Institutet, Stockholm, Sweden.

[4] Department of Medicine Solna, Center for Molecular Medicine, Karolinska University Hospital, Karolinska Institutet, Stockholm, Sweden

[5] Department of Cell and Molecular Biology, Karolinska Institutet, Solna Campus, Stockholm, Sweden

[6] Laboratory of Molecular Genetics and Immunology, Rockefeller University, New York, NY, USA

[7] Princess Margaret Cancer Centre, University Health Network, Toronto, ON, Canada

[*] *Corresponding author:* Mikael C.I. Karlsson: Email: Mikael.Karlsson@ki.se

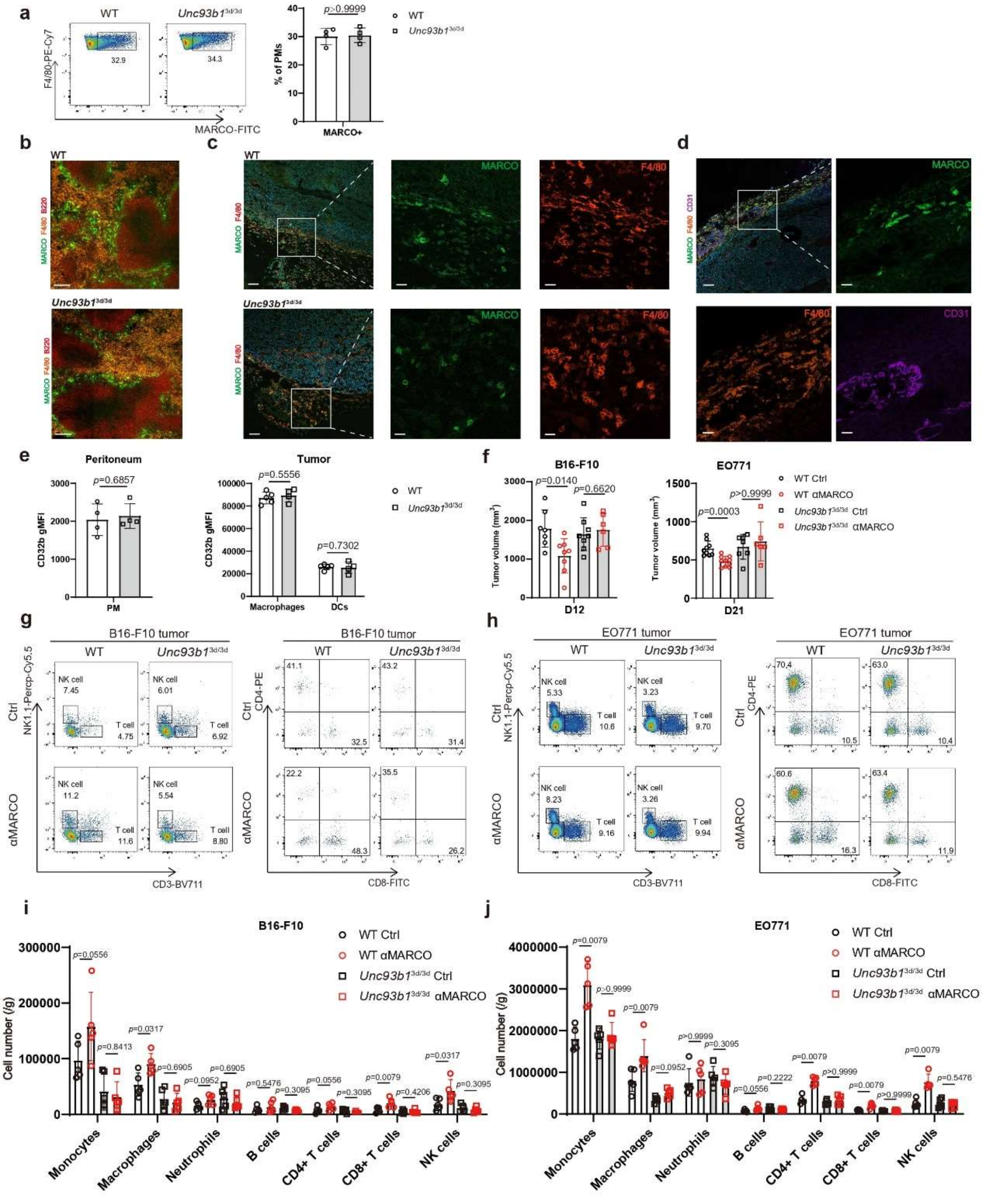

Supplementary Fig. 1 Endosomal TLR signaling is required for anti-MARCO antibody-mediated immune remodeling and tumor suppression

a, Representative plot (left) and graph (right) showing the percentage of MARCO$^+$ cells out of total PMs. n=4 mice per group **b**, Immunofluorescence images of spleen. Scale bar, 100μm. **c**, Immunofluorescence images of B16-F10 tumor. Scale bars, 100μm (left) and 30μm (middle and right). **d,** Immunofluorescence images of B16-F10 tumor from WT mice. Scale bars, 100μm (top-left) and 30μm. **e**, Representative graphs showing the expression of CD32b (FcγRIIB) in peritoneum (left), macrophages and dendritic cells (DC) in EO771 tumor (right). n=4 (peritoneum and *Unc93b1*$^{3d/3d}$ tumor) and 5(WT tumor). **f**, Representative graphs

showing tumor volume of the B16-F10 (left) and EO771 (right) tumor. For B16-F10 tumors: WT Control n=7, WT αMARCO n=8, *Unc93b1*$^{3d/3d}$ Ctrl n=8, *Unc93b1*$^{3d/3d}$ αMARCO n=6; for EO771tumors: WT Ctrl n=9, WT αMARCO n=10, *Unc93b1*$^{3d/3d}$ Ctrl n=7, *Unc93b1*$^{3d/3d}$ αMARCO n=6. **g**, Dot plots showing NK cells (left) and CD8$^+$ T cells (right) in B16-F10. **h**, Dot plots showing NK cells (left) and CD8$^+$ T cells (right) in EO771 tumors. **i**, Representative graphs showing absolute number per gram of immune cell infiltration in B16-F10 tumors. n=5 mice per group. **j**, Representative graphs showing absolute number per gram of immune cell infiltration in EO771 tumors. n=5 mice per group. Representative graph shows one representative experiment out of two independent repeats (**a-e**,**g-j**). Dots represent biological replicates (**a**,**e**,**f**,**i**,**j**). Bar plots show mean ± s.e.m. P values were calculated by Mann-Whitney U test.

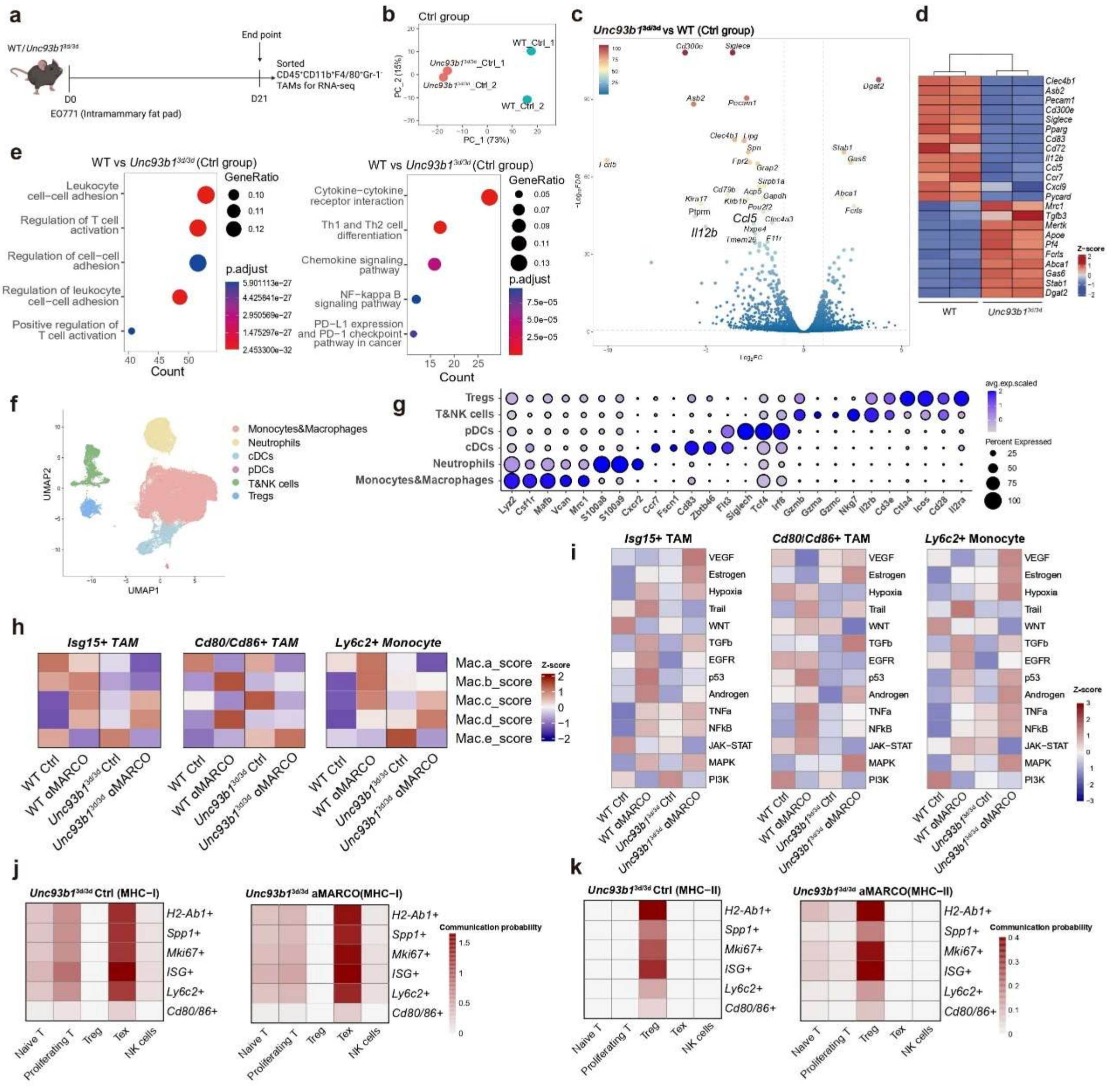

Supplementary Fig. 2 Transcriptomic profiling reveals the essential role of endosomal TLR signaling in anti-MARCO-driven macrophage repolarization

a, Schematic of the experimental design for bulk RNA-seq in the control group. Figure created in BioRender. Liang, H. (2026) https://BioRender.com/kniq4cq. **b**, PCA of the global transcriptomic profiles of TAMs isolated from WT and *Unc93b1*$^{3d/3d}$ tumor-bearing mice in the control group. n=2 samples per group, each sample pooled from three mice's TAMs. **c**, Volcano plot showing DEGs comparing *Unc93b1*$^{3d/3d}$ and WT TAMs under control conditions. Selected top 30 significantly altered genes are highlighted. **d**, Heatmap showing the indicated DEGs from bulk RNA-seq analysis of TAMs isolated from WT versus *Unc93b1*$^{3d/3d}$ mice in EO771 tumors. Colors indicate Z-score normalized expression values. **e**, Functional enrichment analysis of genes upregulated in WT compared to *Unc93b1*$^{3d/3d}$ TAMs in the control group. Dot plots display key Gene Ontology Biological Processes (GO-BP, left) and KEGG pathways (middle). **f**, UMAP visualization of the sorted CD45^{+} immune cell populations from the scRNA-seq dataset of WT and *Unc93b1*$^{3d/3d}$ EO771 tumor-bearing mice. Colors represent distinct cell subpopulations based on marker gene expression. **g**, Dot plot showing the expression profiles of selected marker genes across the annotated CD45^{+} immune cell clusters. Dot size represents the percentage of cells expressing the respective gene, and

color represents the scaled average expression level within each cluster. **h**, Heatmaps showing Scupa-derived normalized polarization scores (Z-scores) of five predefined macrophage states (Mac.a to Mac.e) across the *Isg15*+ TAM, *Cd80/Cd86*+ TAM, and *Ly6c2*+ monocyte subclusters from WT and *Unc93b1*$^{3d/3d}$ tumor-bearing mice under control or αMARCO treatment. **i**, Heatmap showing the normalized pathway activity scores (Z-scores) across the *Isg15*+ TAM, *Cd80/Cd86*+ TAM, and *Ly6c2*+ monocyte subclusters from WT and *Unc93b1*$^{3d/3d}$ tumor-bearing mice under control or αMARCO treatment. Pathway activities for each group were inferred using PROGENy. **j**, Heatmaps showing the communication probability of MHC-I interactions inferred by CellChat from the six monocyte/macrophage subclusters (senders) to major lymphoid cell populations (receivers). The panels compare *Unc93b1*$^{3d/3d}$ tumor-bearing mice treated with control (left) or αMARCO (right). Color intensity represents the communication probability score. **k**, Heatmaps showing the communication probability of MHC-II interactions inferred by CellChat from the six monocyte/macrophage subclusters (senders) to major lymphoid cell populations (receivers). Color intensity represents the communication probability score.

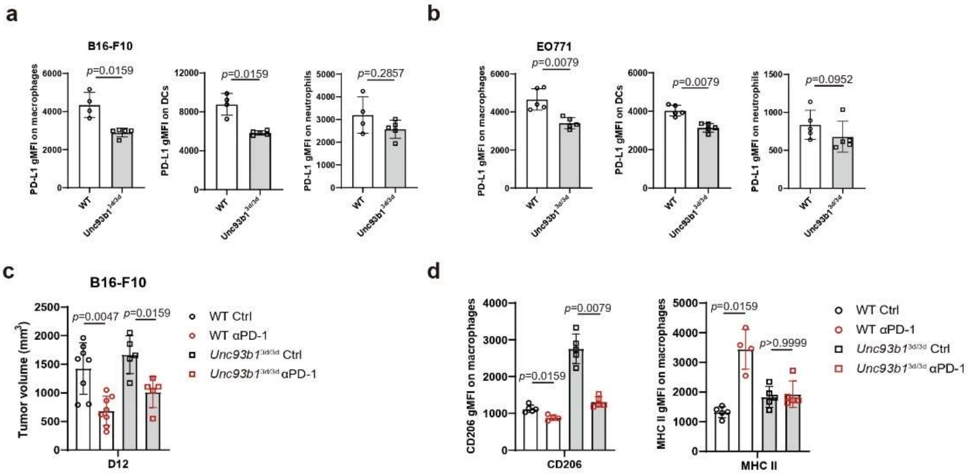

Supplementary Fig. 3 *α*PD-1 treatment operates independently of endosomal TLR signaling

a, Representative graphs showing gMFI of the expression of PD-L1 on macrophages, DCs and neutrophils in B16-F10 tumors at baseline level. WT group n=4, *Unc93b1*$^{3d/3d}$ group n=5. **b**, Representative graphs showing gMFI of the expression of PD-L1 on macrophages, DCs and neutrophils in EO771 tumors at baseline level. WT group n=5, *Unc93b1*$^{3d/3d}$ group n=5. **c**, Tumor volume of the B16-F10 tumors from WT and *Unc93b1*$^{3d/3d}$ mice treated with control or αPD-1 at day 12. WT Ctrl n=8, WT αPD-1 n=8, *Unc93b1*$^{3d/3d}$ Ctrl n=5, *Unc93b1*$^{3d/3d}$ αPD-1 n=5. **d**, Representative graphs showing gMFI of the expression of CD206 and MHCII on TAMs from WT and *Unc93b1*$^{3d/3d}$ mice in EO771 tumors treated with control or αPD-1 at day 21. WT Ctrl n=5, WT αPD-1 n=4, *Unc93b1*$^{3d/3d}$ Ctrl n=5, *Unc93b1*$^{3d/3d}$ αPD-1 n=5. n is the number of mice from one (**a**,**b**,**d**) and two (**c**) independent experiments. Representative graph shows one representative experiment out of two independent repeats (**a-d**). Dots represent biological replicates (**a-d**). Bar plots show mean ± s.e.m. P values were calculated by Mann-Whitney U test.

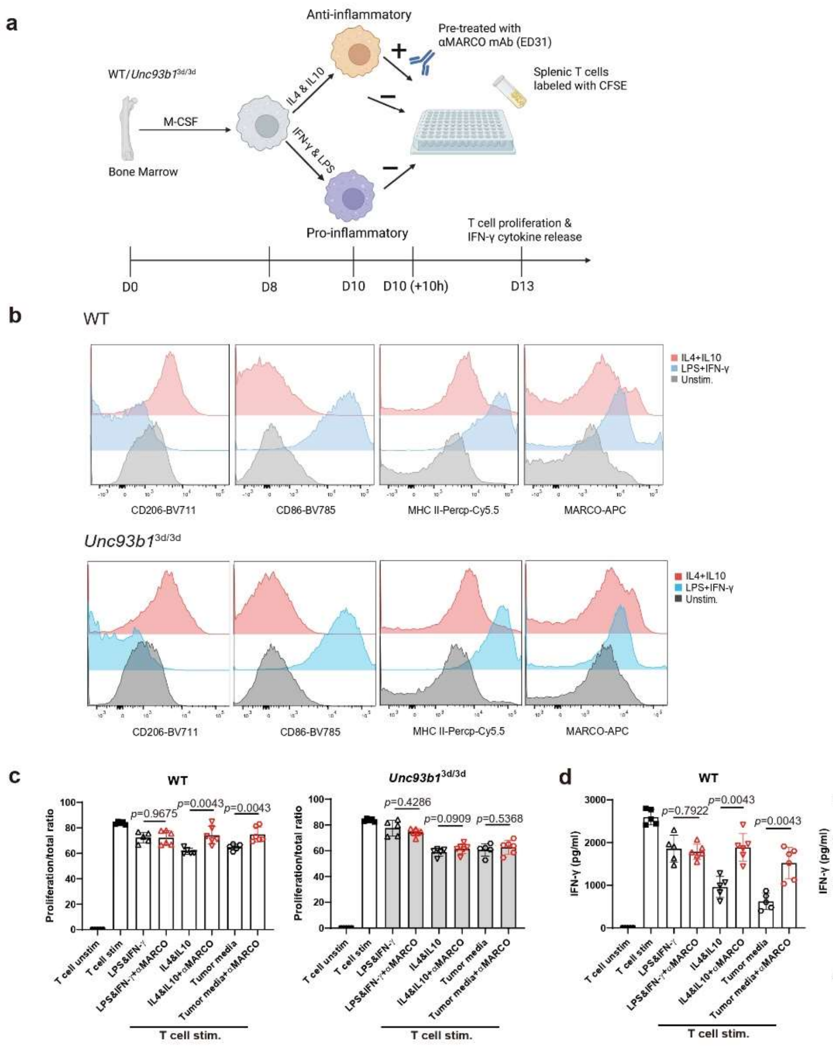

Supplementary Fig. 4 A similar reversal of suppression by αMARCO antibodies was observed in tumor-conditioned media-stimulated macrophages *in vitro*

a, Schematic of *in vitro* coculturing system of bone marrow derived macrophages (BMDMs) with T cells. Figure created in BioRender. Liang, H. (2026) https://BioRender.com/f9oz7x1. **b**, Representative histograms of surface marker of CD206, CD86, MHC II and MARCO expression on unstimulated, LPS&IFN-γ, or IL4&IL10-stimulated BMDMs from WT and *Unc93b1*$^{3d/3d}$ mice. **c**, BMDMs from WT (left) and *Unc93b1*$^{3d/3d}$ (right) mice were stimulated with indicated cytokines or tumor-conditioned media, treated with control or αMARCO antibody, and cocultured with CFSE labeled naive T cells in the presence of CD3/CD28 activator beads for 48h. Representative graphs showing the ratio of proliferating T cells out of total T cells, as assessed by flow cytometry. **d**, Representative graphs showing IFN-γ levels detected in the supernatant from WT (left) or *Unc93b1*$^{3d/3d}$ (right) BMDMs-T cells coculturing system. T cell unstim n=5, T cell stim n=5; for both WT and *Unc93b1*$^{3d/3d}$ BMDMs, LPS&IFN-γ + T cell stim n =5; LPS&IFN-γ + αMARCO + T cell stim n =6; IL4&IL10 + T cell stim n=5; IL4&IL10 BMDM + αMARCO + T cell stim n=6; Tumor media + T cell stim n=5; Tumor media + αMARCO + T cell stim n=6. Representative graph shows one representative experiment out of two independent repeats (**b**,**c**). Barplots show mean ± s.e.m. P values were calculated by Mann-Whitney U test.

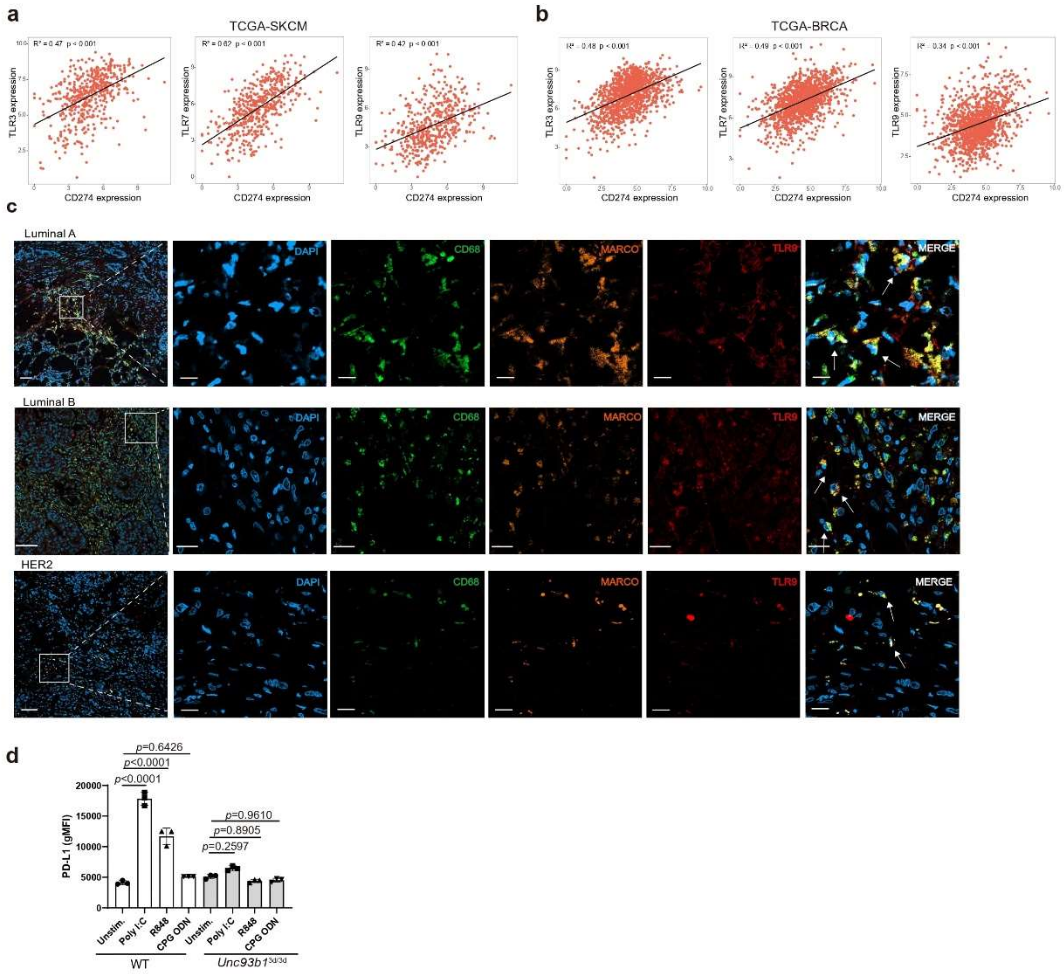

Supplementary Fig. 5 The correlation of MARCO/PD-L1 and endosomal TLRs in humancancer

a, The FPKM expression of *CD274* was plotted against *TLR3*, *TLR7* and *TLR9* in a subset of patients with melanoma included in the TCGA database. Correlation is shown in a xy-plot with a linear regression. P values were calculated by two-tailed Pearson's correlation test. **b**, The FPKM expression of *CD274* was plotted against *TLR3*, *TLR7* and *TLR9* in a subset of patients with breast cancer included in the TCGA database. P values were calculated by two-tailed Pearson's correlation test. **c**, Immunofluorescence analysis of breast cancer from a cohort of n=7 patients. Representative immunofluorescenceimages of patients with Luminal A, Luminal B, Her2 subtypes are shown, respectively. Scale bars, 100μm (far-left) and 20μm. **d**, BMDMs from WT and *Unc93b1*$^{3d/3d}$ mice were stimulated with Poly I:C (TLR3 agonist) (1μg/ml), R848 (TLR7 agonist) (1μg/ml) or CPG ODN (TLR9 agonist) (1μM). Representative graphs showing the gMFI of the expression of PD-L1 from BMDMs under the indicated components stimulation. n=3 mice per group from one experiment. Data are summarized from two independent experiments (**d**). Bar plots show mean ± s.e.m. P values were calculated by ordinary one-way ANOVA followed by Tukey's post hoc test.

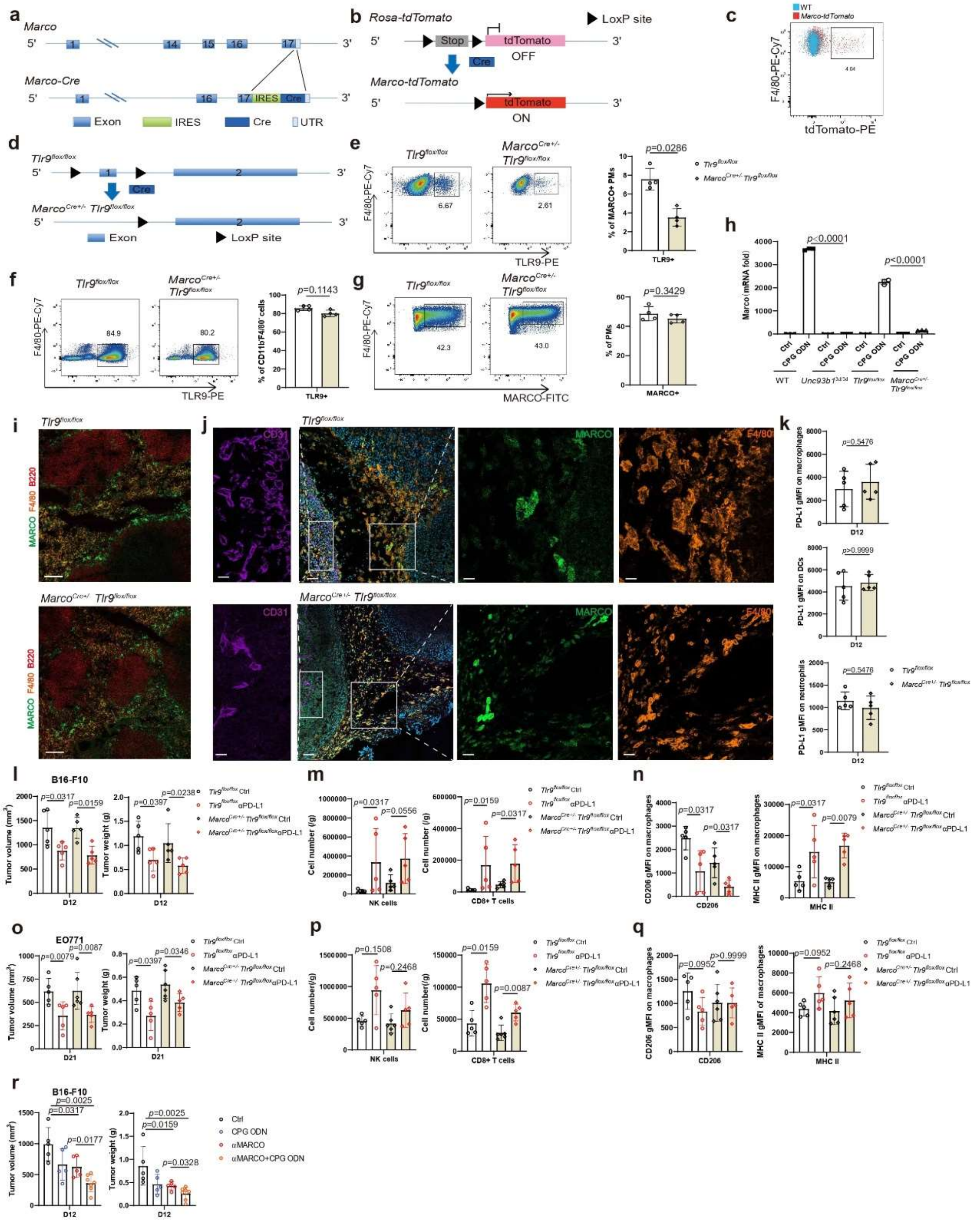

Supplementary Fig. 6 Generation and characterization of *Marco*$^{Cre+/-}$ *Tlr9*$^{flox/flox}$ mice

a, Schematic of the strategy for the creation of *Marco-Cre* mice. **b**, Schematic of the strategy for the creation of *Marco-tdTomato* mice. **c**, Flow cytometry dot plot showing the percentage of tdTomato^{+} TAMs from *Marco-tdTomato* mice in B16-F10 tumor model at day 12. **d**, Schematic of the strategy for the creation of *Marco*$^{Cre+/-}$ *Tlr9*$^{flox/flox}$ mice. **e**, Dot plots (left) and graph (right) showing the percentage of TLR9^{+} cells out of total MARCO^{+} PMs. n=4 mice. **f**, Dot plots (left) and graph (right) showing the percentage of TLR9^{+} cells out of total CD11b^{-} F4/80^{-} non-macrophage cells in peritoneum. n=4 mice. **g**, Dot plots (left) and graph (right) showing the percentage of MARCO^{+} cells out of total PMs. n=4 mice. **h**, Relative gene expression of *Marco* on BMDMs stimulated with or without CPG ODN (TLR9 agonist)

(1μM) for 12h. n=3 mice. **i**, Immunofluorescence images of spleens. Scale bar, 100μm. **j**, Immunofluorescence images of B16-F10 tumor. Scale bars, 100μm (middle-left) and 30μm. **k**, The expression of PD-L1 on macrophages, DCs and neutrophils in B16-F10 tumors at baseline level. n=5 mice per group. **l**, Representative graph showing the volume (left) and weight (right) of B16-F10 tumors treated with control or αPD-L1 antibody at day 12. **m**, Absolute number per gram of NK and $CD8^{+}$T cells as described in **l**. **n**, Expression of CD206 and MHC II on TAMs as described in **l**. n=5 mice. **o**, Representative graph showing the tumor volume (left) and weight (right) of EO771 tumors treated with control or αPD-L1 antibody at day 21. **p**, Absolute number per gram of NK and $CD8^{+}$T cells as described in **o**. **q**, The expression of CD206 and MHC II on TAMs as described in **o**. n=5 and 6 ($Marco^{Cre+/-}$ $Tlr9^{flox/flox}$ Ctrl) mice per group. **r**, Volume (left) and tumor weight (right) of B16-F10 tumor-bearing WT mice treated with CpG ODN (20μg), αMARCO (100μg), or a combination of αMARCO and CpG ODN at day 12. n=5 and 7 (combo therapy) mice per group. Bar plots show mean ± s.e.m. P values were calculated by ordinary one-way ANOVA followed by Tukey's post hoc test (**h**) and Mann-Whitney U test (**e-g**,**k-r**).

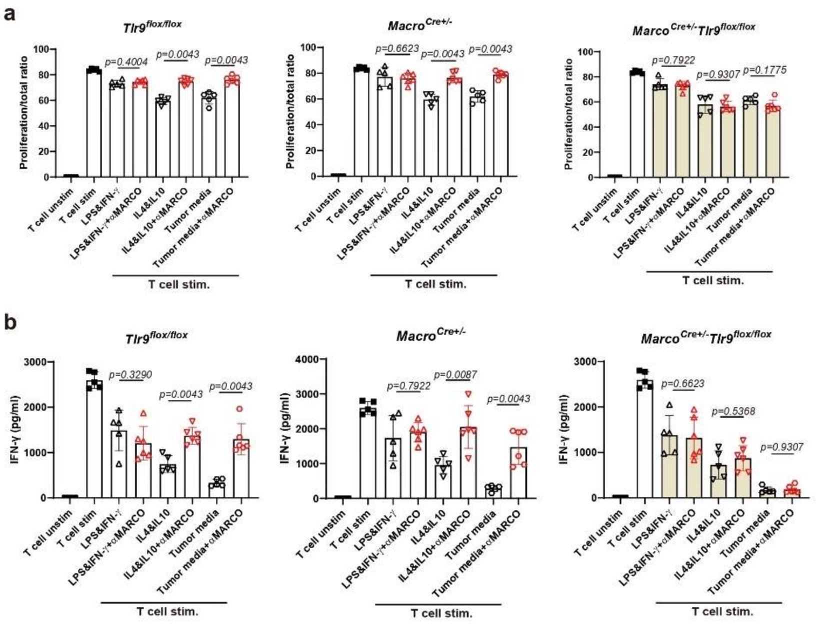

Supplementary Fig. 7 anti-MARCO restores macrophage-mediated T cell activation via the TLR9 signaling pathway

a, BMDMs from $Tlr9^{flox/flox}$ (left), $Marco^{Cre+/-}$ (middle) and $Marco^{Cre+/-}$ $Tlr9^{flox/flox}$ (right) mice were stimulated with indicated cytokines or tumor-conditioned media, treated with control or αMARCO antibody, and cocultured with CFSE labeled naive T cells in the presence of CD3/CD28 activator beads for 48h. Representative graphs showing the ratio of proliferating T cells out of total T cells, as assessed by flow cytometry. **b**, Representative graphs showing IFN-γ levels detected in the supernatant from $Tlr9^{flox/flox}$ (left), $Marco^{Cre+/-}$ (middle) and $Marco^{Cre+/-}$ $Tlr9^{flox/flox}$ (right) BMDMs-T cells coculturing system. T cell unstim n=5, T cell stim n=5; for all the $Tlr9^{flox/flox}$, $Marco^{Cre+/-}$ and $Marco^{Cre+/-}$ $Tlr9^{flox/flox}$ BMDMs, LPS&IFN-γ + T cell stim n =5; LPS&IFN-γ + αMARCO + T cell stim n =6; IL4&IL10 + T cell stim n=5; IL4&IL10 BMDM + αMARCO + T cell stim n=6; Tumor media + T cell stim n=5; Tumor media + αMARCO + T cell stim n=6. Representative graph shows one representative experiment out of two independent repeats. Barplots show mean ± s.e.m. P values were calculated by Mann-Whitney U test.

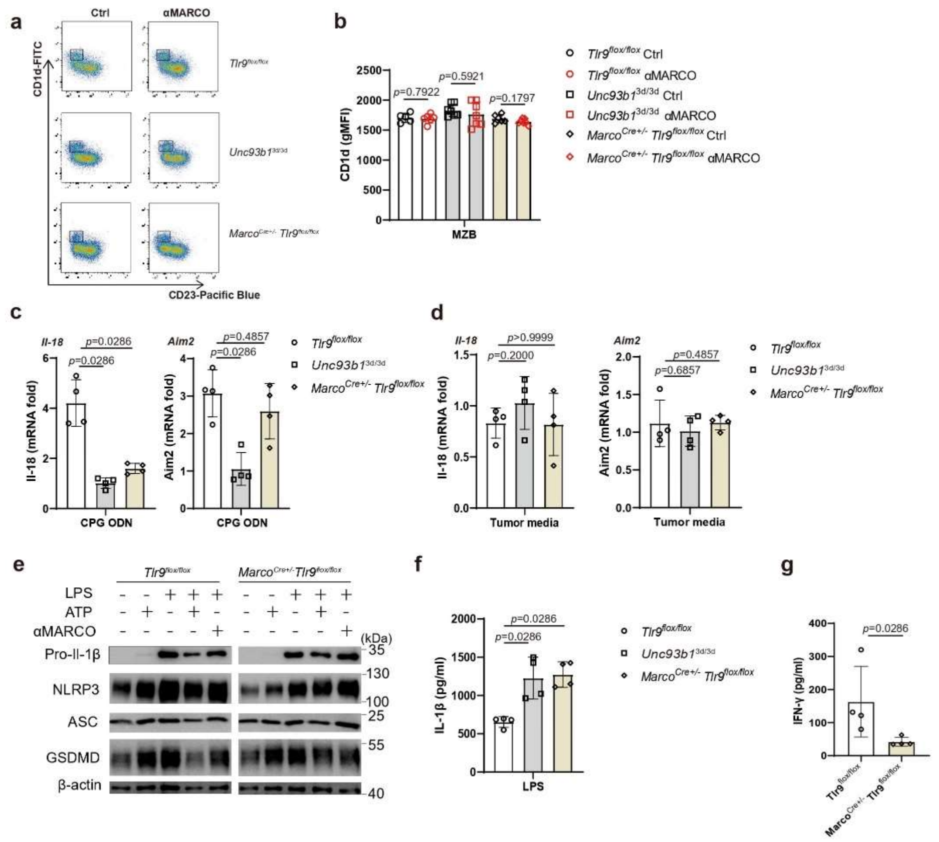

Supplementary Fig. 8 NLRP3 inflammasome activation is specific to the TLR9-ATP axis within MARCO-expressing cells

a, Spleens were harvested from *Tlr9*$^{flox/flox}$, *Unc93b1*$^{3d/3d}$ and *Marco*$^{Cre+/-}$ *Tlr9*$^{flox/flox}$ mice that were injected (i.v.) with control or αMARCO (50μg/mouse) for 24h. Representative dot plots showing the gating of CD1d^{+} marginal zone B (MZB). **b**, Representative graph showing gMFI of the expression of CD1d on MZB. *Tlr9*$^{flox/flox}$ Ctrl n=5, *Tlr9*$^{flox/flox}$ αMARCO n=6, *Unc93b1*$^{3d/3d}$ Ctrl n=7, *Unc93b1*$^{3d/3d}$ αMARCO n=7, *Marco*$^{Cre+/-}$ *Tlr9*$^{flox/flox}$ Ctrl n=6, *Marco*$^{Cre+/-}$ *Tlr9*$^{flox/flox}$ αMARCO n=6. **c**, Relative expressionof *Il-18* and *Aim2* on PMs from *Tlr9*$^{flox/flox}$, *Unc93b1*$^{3d/3d}$ and *Marco*$^{Cre+/-}$ *Tlr9*$^{flox/flox}$ mice stimulated with CPG ODN (TLR9 agonist) (1μM) for 12h. n=4 mice per group. **d**, Relative expressionof *Il-18* and *Aim2* on PMs from *Tlr9*$^{flox/flox}$, *Unc93b1*$^{3d/3d}$ and *Marco*$^{Cre+/-}$ *Tlr9*$^{flox/flox}$ mice stimulated with tumor media for 12h. n=4 mice per group. **e**, Western blot (WB) analysis of inflammasome-related proteins in PMs isolated from *Tlr9*$^{flox/flox}$ and *Marco*$^{Cre+/-}$ *Tlr9*$^{flox/flox}$ mice. Cells were stimulated with LPS (100ng/ml) for 12h following with ATP (5mM) or αMARCO (10μg/ml) for 30min. Representative blots show protein levels of Pro-IL-1β, NLRP3, ASC, and GSDMD. Numbers on the right indicate molecular weights in kDa. **f**, IL-1β levels detected in the supernatant of PMs from *Tlr9*$^{flox/flox}$, *Unc93b1*$^{3d/3d}$ and *Marco*$^{Cre+/-}$ *Tlr9*$^{flox/flox}$ mice stimulated with LPS (100ng/ml) for12h following with ATP (5mM) for 30min. n=4 mice per group. **g**, IFN-γ levels detected in the supernatant of B16-F10 tumor-conditioned media from *Tlr9*$^{flox/flox}$ and *Marco*$^{Cre+/-}$ *Tlr9*$^{flox/flox}$ mice after 24 hours cultured. n=4 mice per group. Representative graph shows one representative experiment out of two independent repeats (**a-g**). Dots represent biological replicates (**b-d**,**f**,**g**). Bar plots show mean ± s.e.m. P values were calculated by Mann-Whitney U test.

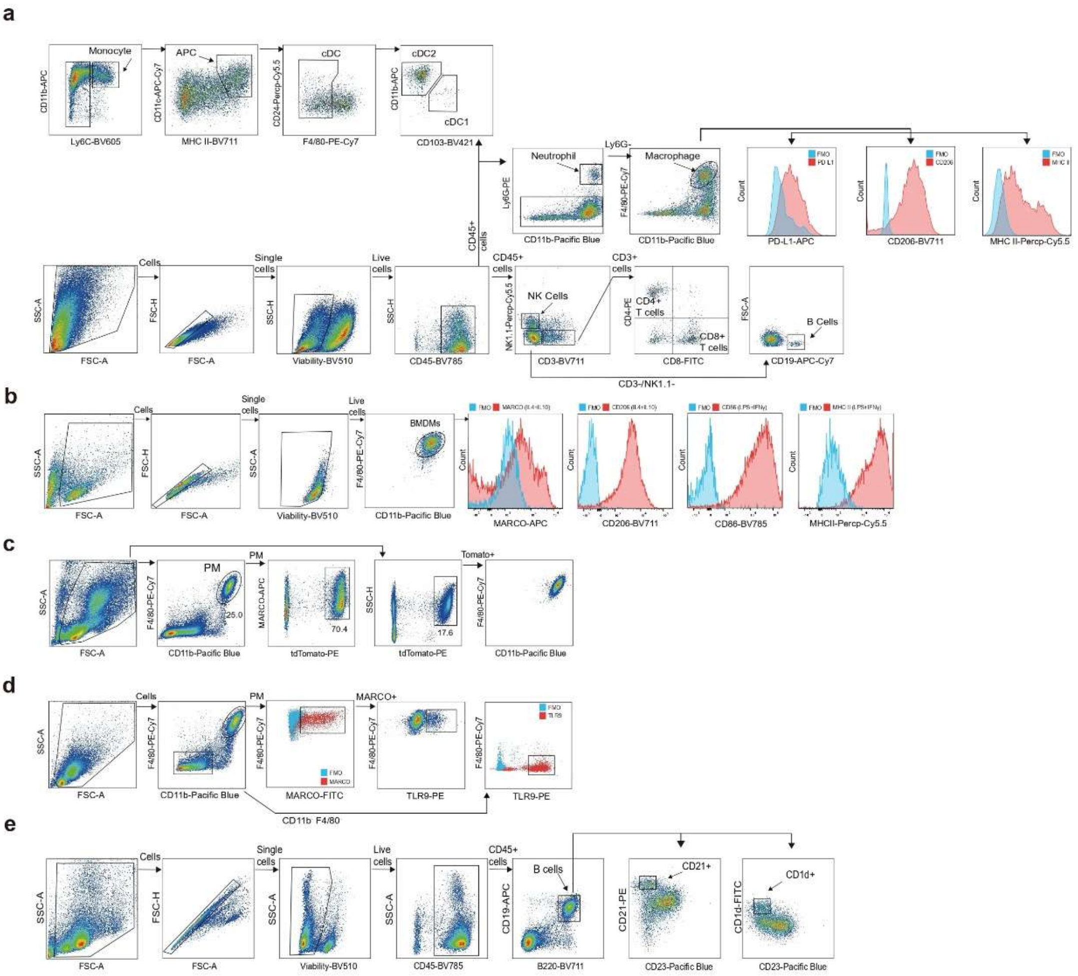

Supplementary Fig. 9 Gating strategies in this study

a, Gating strategy for lymphoid cells and myeloid cells within tumor presented on Fig. 1g-i; Fig. 2e-h, j; Fig. 6c-f, h; Supplementary Fig. 1e, g-j; Supplementary Fig. 3a, b, d; Supplementary Fig. 6k, m, n, p, q.

b, Gating strategy for BMDMs in vitro presented on Fig. 4a; Fig. 5d; Fig. 7d, e; Supplementary Fig. 4b; Supplementary Fig. 5d.

c, Gating strategy for PMs in *Marco-tdTomato* mice and PMs presented on Supplementary Fig. 1a, e.

d, Gating strategy for PMs in *Marco*$^{Cre+/-}$ *Tlr9*$^{flox/flox}$ mice presented on Supplementary Fig. 6e-g.

e, Gating strategy for MZB cells (B220^{+}CD19^{+}CD23^{-}CD1d^{+}) in spleen presented on Fig. 8b, c; Supplementary Fig. 8a, b.

Supplementary Table 1 Antibodies used in the study

	Target	Conjugate	Vendor	Catalog#	Dilution	App.
Anti-mouse	CD1d	FITC	BioLegend	123508	1:200	FC
	CD3ε	BV711		100349	1:500	
	CD8a	FITC		100706	1:400	
	CD11b	Pacific blue		101224	1:200	
	CD11b	APC		101212	1:200	
	CD11c	APC-Cy7		117324	1:300	
	CD19	APC		152410	1:400	
	CD21/CD35	PE		123410	1:200	
	CD23	Pacific blue		101616	1:300	
	CD24	PerCP-Cy5.5		101824	1:200	
	CD45	BV785		103149	1:400	
	CD86	BV785		105043	1:300	
	CD103	BV421		121422	1:200	
	CD206	BV650		141723	1:400	
	CD206	BV711		141727	1:400	
	CD274	APC		124312	1:400	
	F4/80	PE/Cy7		123114	1:400,	
	MHCII	PerCP-Cy5.5		107626	1:200	
	Ly6C	BV605		128035	1:500	
	Ly6G	PE		127608	1:500	
	TLR9	PE		159103	1:100	
	Viability	Zombia Aqua		423102	1:1000	
	CD4	PE	BD Biosciences	553048	1:400	
	CD19	APC-H7		560143	1:400	
	B220	BV711		563892	1:500	
	MHCII	BV711		563414	1:200	
	NK1.1	PerCP-Cy5.5		551114	1:200	
	CD32b	PE	eBioscience	3196365	1:300	
	F4/80	eFluor 570	Invitrogen	41-4801-80	1:100	IF
	CD31	AF647	BioLegend	102516	1:100	
	B220	AF647	BD Biosciences	557683	1:100	
Anti-human	TLR9	unconjugated	Abcam	ab134368	1:100	
Anti-human	CD68	AF488	Santa Cruz Biotechnology	K2106	1:100	
	secondary	AF647	Invitrogen	A21236	1:1000	
Anti-mouse	NLRP3	N/A	Cell Signaling Technology	15101	1:1000	WB
	ASC	N/A		67824	1:1000	
	Pro-IL-1β	N/A		31202	1:1000	

	GSDMD	N/A	Abcam	ab219800	1:1000	
	β-actin	HRP		ab20272	1:5000	
Anti-rabbit	secondary	HRP	Cell Signaling Technology	7074	1:2000	

Supplementary Table 2 DNA oligonucleotide sequences used in the study

Target gene	Primer labels	Sequences
mMARCO	Forward	GGCACCCCAAGGGAGACAAA
	Reverse	TCCCTTCATGCCATGTC
mMrc1	Forward	CTCTGTTCAGCTATTGGACGC
	Reverse	TGGCACTCCCAAACATAATTTGA
mArg1	Forward	CCACAGTCTGGCAGTTGGAAG
	Reverse	GGTTGTCAGGGGAGTGTTGATG
mNos2	Forward	CGAAACGCTTCACTTCCAA
	Reverse	TGAGCCTATATTGCTGTGGCT
mH2-Ab1	Forward	TTTGCTTTCTGAAGGGGGCA
	Reverse	TCGCCCATGAACTGGTACAC
mCd86	Forward	TCAATGGGACTGCATATCTGCC
	Reverse	GCCAAAATACTACCAGCTCACT
mIl-1β	Forward	TGGAAAAGCGGTTTGTCT
	Reverse	ATAAATAGGTAAGTGGTTGCC
mNlrp3	Forward	TGCTCTTCACTGCTATCAAGCCCT
	Reverse	ACAAGCCTTTGCTCCAGACCCTAT
mIl-18	Forward	TGGTTCCATGCTTTCTGGACTCCT
	Reverse	TTCCTGGGCCAAGAGGAAGTGATT
mIfnβ	Forward	GACGGAGAAGATGCAGAAGAGTT
	Reverse	AGTTCATCCAGGAGACGTACAAC
mAim2	Forward	AGGCTGCTACAGAAGTCTGTCC
	Reverse	TCAGCACCGTGACAACAAGTGG
