# Supplementary material for: Cancer immunotherapy targeting murine myeloid cells requires endosomal pattern recognition
Source: Nat Commun. 2026 Jul 27;17:7410. doi: 10.1038/s41467-026-75543-2 (PMC13408345; doi:10.1038/s41467-026-75543-2)
Supplement: Supplementary file 2 — Reporting Summary [file 41467_2026_75543_MOESM2_ESM.pdf]

nature portfolio

Corresponding author(s): Mikael Karlsson

Last updated by author(s): Jun 1, 2026

Reporting Summary

Nature Portfolio wishes to improve the reproducibility of the work that we publish. This form provides structure for consistency and transparency in reporting. For further information on Nature Portfolio policies, see our Editorial Policies and the Editorial Policy Checklist.

Statistics

For all statistical analyses, confirm that the following items are present in the figure legend, table legend, main text, or Methods section.

n/a	Confirmed	
☐	☒	The exact sample size (*n*) for each experimental group/condition, given as a discrete number and unit of measurement
☐	☒	A statement on whether measurements were taken from distinct samples or whether the same sample was measured repeatedly
☐	☒	The statistical test(s) used AND whether they are one- or two-sided<br>*Only common tests should be described solely by name; describe more complex techniques in the Methods section.*
☒	☐	A description of all covariates tested
☐	☒	A description of any assumptions or corrections, such as tests of normality and adjustment for multiple comparisons
☐	☒	A full description of the statistical parameters including central tendency (e.g. means) or other basic estimates (e.g. regression coefficient) AND variation (e.g. standard deviation) or associated estimates of uncertainty (e.g. confidence intervals)
☐	☒	For null hypothesis testing, the test statistic (e.g. *F*, *t*, *r*) with confidence intervals, effect sizes, degrees of freedom and *P* value noted<br>*Give P values as exact values whenever suitable.*
☒	☐	For Bayesian analysis, information on the choice of priors and Markov chain Monte Carlo settings
☒	☐	For hierarchical and complex designs, identification of the appropriate level for tests and full reporting of outcomes
☐	☒	Estimates of effect sizes (e.g. Cohen's *d*, Pearson's *r*), indicating how they were calculated

Our web collection on statistics for biologists contains articles on many of the points above.

Software and code

Policy information about availability of computer code

Data collection	Microscopy images for immunofluorescence were acquired on Zeiss LSM980-Airy confocal microscope with the ZEN software.<br><br>Flow cytometry data were collected with on BD LSR Fortessa X-20 analyzer or BD FACS Aria Fusion sorter with BD FACSDIVA software.<br><br>Sequencing was performed on the Illumina NovaSeq 6000 sequencer and DNBSEQ-G400 sequencer.<br><br>C1000 Touch Thermal Cycler equipped with a CFX96 Real-Time System (Bio-Rad) was used for qRT-PCR with CFX Maestro software.
Data analysis	Microscopy images were analyzed with ImageJ (v1.52h).<br><br>Flow cytometry data was analyzed with the FlowJo software (v10.8.1).<br><br>RNA-seq data sets were analyzed using the DESeq2 R package (v1.40.2) and Seurat (v4.4.0) in the R software (v4.2.0).<br><br>Statistical analysis was performed using GraphPad Prism software (v8.0.2).

For manuscripts utilizing custom algorithms or software that are central to the research but not yet described in published literature, software must be made available to editors and reviewers. We strongly encourage code deposition in a community repository (e.g. GitHub). See the Nature Portfolio guidelines for submitting code & software for further information.

Data

Policy information about availability of data

All manuscripts must include a data availability statement. This statement should provide the following information, where applicable:

- Accession codes, unique identifiers, or web links for publicly available datasets
- A description of any restrictions on data availability
- For clinical datasets or third party data, please ensure that the statement adheres to our policy

Publicly available datasets: The Cancer Genome Atlas (TCGA)- SKCM project and BRCA project (Web links: http://cancergenome.nih.gov and https://xenabrowser.net/)
Single-cell and bulk RNA-seq data have been deposited at NCBI GEO data repository under accession number GSE303908 and NCBI SRA data repository under accession number SRP604259

Research involving human participants, their data, or biological material

Policy information about studies with human participants or human data. See also policy information about sex, gender (identity/presentation), and sexual orientation and race, ethnicity and racism.

Reporting on sex and gender	A total of ten female patients with four different breast cancer subtypes were recruited for this study.
Reporting on race, ethnicity, or other socially relevant groupings	Race, ethnicity or other socially relevant information have not been collected.
Population characteristics	Histological evaluations of patient tumors were evaluated by pathologists for diagnostic purposes: tumor characteristics, including hormone receptors, HER2, and Ki67 expression, and were classified as Luminal A, Luminal B, HER2, and triple-negative breast cancer (TNBC). Samples from Luminal A, Luminal B, and HER2 were all from treatment-naïve patients. Samples from two TNBC patients received neoadjuvant chemotherapy, but both stopped halfway and underwent surgery. All patient samples were anonymized with only the treatment status disclosed.
Recruitment	Patient samples have been obtained as part of routine diagnosis. All procedures were in accordance with the national guidelines of the Department of Clinical Pathology and Cancer Diagnostics at Karolinska University Hospital (Stockholm).
Ethics oversight	Experimental procedures and protocols of the study were previously approved by the regional ethics review board (Etikprövningsnämnden) in Stockholm. All patients signed a written informed consent and did not receive compensation.

Note that full information on the approval of the study protocol must also be provided in the manuscript.

Field-specific reporting

Please select the one below that is the best fit for your research. If you are not sure, read the appropriate sections before making your selection.

- [x] Life sciences
- [ ] Behavioural & social sciences
- [ ] Ecological, evolutionary & environmental sciences

For a reference copy of the document with all sections, see nature.com/documents/nr-reporting-summary-flat.pdf

Life sciences study design

All studies must disclose on these points even when the disclosure is negative.

Sample size	No formal statistical methods were used to predetermine sample sizes, but they were chosen based on prior experience and previous publications for the same type of experiments from our lab (Eisinger et al. PNAS 2020). The exact sample sizes (n) are given in the Figure legends. A minimum of 3 mice per group was used in each independent experiment, and all experiments were highly reproducible.
Data exclusions	Animals were excluded from experiments if they died, or had to be killed to comply to ethical regulations.
Replication	Representative plots and graphs summarize results of at least two to three independent experiments.
Randomization	Animals were matched for age and sex to minimize potential confounding variables and randomly allocated to experimental groups.
Blinding	The researchers were not blinded to allocation during experiments and outcome assessments as analyses were performed using predefined, objective criteria.

Reporting for specific materials, systems and methods

We require information from authors about some types of materials, experimental systems and methods used in many studies. Here, indicate whether each material, system or method listed is relevant to your study. If you are not sure if a list item applies to your research, read the appropriate section before selecting a response.

Materials & experimental systems

n/a	Involved in the study
	☒ Antibodies
	☒ Eukaryotic cell lines
☒	Palaeontology and archaeology
	☒ Animals and other organisms
☒	Clinical data
☒	Dual use research of concern
☒	Plants

Methods

n/a	Involved in the study
☒	ChIP-seq
	☒ Flow cytometry
☒	MRI-based neuroimaging

Antibodies

Antibodies used

Antibodies are described in this order: target, catalog number, clone name, and dilution to ensure precise identification of the reagent used.

Antibodies for immunofluorescence (IF) staining:
Rat anti-mouse MARCO produced by ED31 hybridoma and conjugated to AF488 (Invitrogen, A20181, 2563692), Rat anti-mouse F4/80 Antibody eFluor 570 (Invitrogen, Cat#41-4801-80, BM8, 1:100, RRID: AB_2573611), Rat anti-mouse CD31 Antibody AF647 (BioLegend, Cat#102516, MEC13.3, 1:100, RRID: AB_2161029), Rat anti-mouse B220 Antibody AF647 (BD Biosciences, Cat#557683, RA3-6B2, 1:100, RRID: AB_396793), Mouse anti-human TLR9 (Abcam, Cat#ab134368, 26C593.2, 1:100), Goat anti-mouse IgG (H+L) AF647 Antibody (Invitrogen, Cat#A21236, Polyclonal, 1:1000), Mouse anti-human MARCO produced by 71H01 hybridoma and conjugated to AF555 (Life Technologies, A20187, 2057467), anti-human CD68 AF488 (Santa Cruz Biotechnology, Cat# K2106, KP1, 1:100).

Antibodies for flow cytometry:
anti-mouse CD1d Antibody FITC (BioLegend, Cat#123508, 1B1, 1:200, RRID: AB_1236549), anti-mouse CD3ε Antibody BV711 (BioLegend, Cat#100349, 145-2C11, 1:500, RRID: AB_2565841), anti-mouse CD4 Antibody PE (BD Biosciences, Cat#553048, RM4-5, 1:400, RRID: AB_394585), anti-mouse CD8a Antibody FITC (BioLegend, Cat#100706, 53-6.7, 1:400, RRID: AB_312745), anti-mouse CD11b Antibody Pacific blue (BioLegend, Cat#101224, M1/70, 1:200, RRID: AB_755986), anti-mouse CD11b Antibody APC (BioLegend, Cat#101212, M1/70, 1:200, RRID: AB_312795), anti-mouse CD11c Antibody APC-Cy7 (BioLegend, Cat#117324, N418, 1:300, RRID: AB_830649), anti-mouse CD19 Antibody APC (BioLegend, Cat#152410, 1D3, 1:400, RRID: AB_2629839), anti-mouse CD19 Antibody APC-H7 (BD Biosciences, Cat#560143, 1D3, 1:400, RRID: AB_1645234), anti-mouse CD21/CD35(CR2/CR1) Antibody PE (BioLegend, Cat#123410, 7E9, 1:200, RRID: AB_940413), anti-mouse CD23 Antibody Pacific blue (BioLegend, Cat#101616, B3B4, 1:300, RRID: AB_2103306), anti-mouse CD24 Antibody PerCP-Cy5.5 (BioLegend, Cat#101824, M1/69, 1:200, RRID: AB_1595491), anti-mouse CD32b Antibody PE(eBioscience, lot#3196365, AT130-2, 1:300) anti-mouse CD45 Antibody BV785 (BioLegend, Cat#103149, 30-F11, 1:400, RRID: AB_2564590), anti-mouse CD86 Antibody BV785 (BioLegend, Cat#105043, GL-1, 1:300, RRID: AB_2566722), anti-mouse CD103 Antibody BV421 (BioLegend, Cat#121422, 2E7, 1:200, RRID: AB_2562901), anti-mouse CD206 Antibody BV650 (BioLegend, Cat#141723, C068C2, 1:400, RRID: AB_2562445), anti-mouse CD206 Antibody BV711 (BioLegend, Cat#141727, C068C2, 1:400, RRID: AB_2565822), anti-mouse CD274(PD-L1) Antibody APC (BioLegend, Cat#124312, 10F.9G2, 1:400, RRID: AB_10612741), anti-mouse B220 Antibody BV711 (BD Biosciences, Cat#563892, RA3-6B2, 1:500, RRID: AB_2738470), anti-mouse F4/80 Antibody PE/Cy7 (BioLegend, Cat#123114, BM8, 1:400, RRID: AB_893478), anti-mouse MHCII Antibody PerCP-Cy5.5 (BioLegend, Cat#107626, M5/114.15.2, 1:200, RRID: AB_2191071), anti-mouse MHCII Antibody BV711 (BD Biosciences, Cat#563414, M5/114.15.2, 1:200, RRID: AB_2738191), anti-mouse Ly6C Antibody BV605 (BioLegend, Cat#128035, HK1.4, 1:500, RRID: AB_2562352), anti-mouse Ly6G Antibody PE (BioLegend, Cat#127608, 1A8, 1:500, RRID: AB_1186099), anti-mouse TLR9 Antibody PE (BioLegend, Cat#159103, S18025A, 1:100, RRID: AB_2876552), anti-mouse NK1.1 Antibody PerCP-Cy5.5 (BD Biosciences, Cat#551114, PK136, 1:200, RRID: AB_394052), anti-mouse MARCO produced by ED31 hybridoma and conjugated to AF488 (Life Technologies, A20181, 2563692), anti-mouse MARCO produced by ED31 hybridoma and conjugated to AF647 (Life Technologies, A20186, 1831225).

For in vivo experiments:
Rat IgG1 anti-mouse MARCO Antibody (Mabtech, ED31, 100μg/mouse); InVivoMAb rat IgG1 isotype control (Bio X Cell, Cat#BE0290, TNP6A7); InVivoMAb anti-mouse PD-1 (CD279) (Bio X Cell, Cat#BE0146, RMP1-14, 180μg/mouse); InVivoMAb anti-mouse PD-L1 (B7-H1) (Bio X Cell, Cat#BE0101, 10F.9G2, 75μg/mouse); CPG ODN (Invivogen, #tlrl-2395, 20μg/mouse)

Validation

All primary antibodies were purchased from well-established commercial suppliers recognized for quality and reproducibility. Antibodies were titrated and tested prior to use in each experimental setup. For IF staining, specificity of antibodies was assessed with negative controls. For flow cytometry experiments, isotype controls and fluorescence minus one (FMO) controls were done to evaluate antibody specificity. For the anti-mouse MARCO antibody, we also assessed specificity using a MARCO knock-out mouse.

Eukaryotic cell lines

Policy information about cell lines and Sex and Gender in Research

Cell line source(s)

The B16-F10 cell line and EO771 cell line were purchased from the American Type Culture Collection (ATCC).

Authentication

Cell lines were authenticated by morphological criteria only. Cells used for experiments were cultured for no more than five passages.

Mycoplasma contamination

All cell lines tested negative for mycoplasma contamination.

Commonly misidentified lines
(See ICLAC register)

No commonly misidentified cell lines were used.

Animals and other research organisms

Policy information about studies involving animals; ARRIVE guidelines recommended for reporting animal research, and Sex and Gender in Research

Laboratory animals

8-12 week-old male mice and 8-14 week-old female mice were used in this study.
C57BL/6J (BL/6J) mice were purchased from Charles River Laboratories.
Tlr9flox/flox (C57BL/6J-Tlr9em1Ldm/J) mice (Strain #:034448, RRID:IMSR_JAX:034448) were purchased from Jackson Laboratory.
Rosa-tdTomato reporter mice (B6.Cg-Gt(ROSA)26Sortm14(CAG-tdTomato)Hze/J) (Strain #:007914, RRID:IMSR_JAX:007914) were obtained from Jackson Laboratory.
Unc93b13d/3d (Tabeta et al. 2006), MARCO-Cre (Lamorte et al. 2025) were used in this study.

Wild animals

No wild animals were used for this study.

Reporting on sex

Only female mice were used in experiments performed with the murine breast cancer (EO771) mouse model since tumor normally don't develop in male mice. Only male mice were used in experiments performed with the murine melanoma (B16-F10) cancer model.

Field-collected samples

This study did not involve field-collected samples.

Ethics oversight

All animal experimental procedure were approved by the Stockholm's Animal Experimental Ethical Committee (Dnr 4240-2021).

Note that full information on the approval of the study protocol must also be provided in the manuscript.

Plants

Seed stocks

Report on the source of all seed stocks or other plant material used. If applicable, state the seed stock centre and catalogue number. If plant specimens were collected from the field, describe the collection location, date and sampling procedures.

Novel plant genotypes

Describe the methods by which all novel plant genotypes were produced. This includes those generated by transgenic approaches, gene editing, chemical/radiation-based mutagenesis and hybridization. For transgenic lines, describe the transformation method, the number of independent lines analyzed and the generation upon which experiments were performed. For gene-edited lines, describe the editor used, the endogenous sequence targeted for editing, the targeting guide RNA sequence (if applicable) and how the editor was applied.

Authentication

Describe any authentication procedures for each seed stock used or novel genotype generated. Describe any experiments used to assess the effect of a mutation and, where applicable, how potential secondary effects (e.g. second site T-DNA insertions, mosiacism, off-target gene editing) were examined.

Flow Cytometry

Plots

Confirm that:

- [x] The axis labels state the marker and fluorochrome used (e.g. CD4-FITC).
- [x] The axis scales are clearly visible. Include numbers along axes only for bottom left plot of group (a 'group' is an analysis of identical markers).
- [x] All plots are contour plots with outliers or pseudocolor plots.
- [x] A numerical value for number of cells or percentage (with statistics) is provided.

Methodology

Sample preparation

Tumors were isolated, cut into pieces and enzymatically dissociated using 100µg/ml DNAse (Roche), 150µg/ml Liberase TL (Roche) for 30 min at 37°C water baths. After digestion, cells were passed through 100µm filter strainer and washed thoroughly to obtain a single-cell suspension. For positive selection by magnetic cell isolation, cells were labeled with mouse CD45 MicroBeads (Miltenyi Biotec, Cat#130-052-301) followed with the kit protocol. Purified CD45-positive cells were resuspended and ready for further staining.

Spleen was dissected out, mushed through a 70 µm strainer, and followed by lysis of RBC (Gibco) before staining.

Non-specific labeling was blocked with anti-CD16/32 (FC Block, BD Biosciences, Cat#553142, 2.4G2, 1:500, RRID: AB_394656) together with viability assessment using LIVE/DEAD Fixable Aqua (Invitrogen, REF:L34957) for 15 min at room temperature (RT) before specific labeling. Subsequently cells were stained with fluorescently labelled antibodies for 30 min at 4°C.

Instrument

Flow cytometry data collection have been done on BD LSR Fortessa X-20 analyzer or BD FACS Aria Fusion sorter.

Software	Flow cytometry data was collected with BD FACS DIVA software.<br>Flow cytometry data analyzed with the FlowJo software (v10.8.1)
Cell population abundance	For CD45+ CD11b+ F4/80+ Gr-1- TAMs sorting, we performed a test-sort. The purity of the test-sort fraction had to exceed >90% for us to continue sorting. For murine splenic T cells isolation via negative selection (Mouse pan T cell isolation kit, Miltenyi, Cat#130-095-130), we performed a test-selection and the isolation T cells had a final cell purity exceeded 95%.
Gating strategy	Our gating strategy to define murine immune cells by flow cytometry started by excluding doublets, where the forward scatter height (FSC-H) to forward scatter area (FSC-A) was compared. Next, to exclude dead cells, we used a viability dye (LIVE/DEAD Fixable Aqua) and only considered the live cells. Within these cells CD45 identifies immune cells.<br><br>We defined the following immune cell subpopulations in the CD45 gate from tumor samples, as listed below:<br>For lymphoid cells gating, we gated NK1.1 positive NK cells, CD3e positive T cells and CD19 positive B cells. The T cells were further subdivided in CD4 Positive and CD8a positive subsets.<br><br>For myeloid cells gating, we first gated out CD11b and Ly6G double positive neutrophils, and CD11b and Ly6C double positive monocytes. Next, we gated the rest cells by using CD11b and F4/80 double positive macrophages, and CD11c and MHC II double positive, F4/80 negative dendritic cells. Macrophages subsets were further analyzed for activation/polarization markers CD206, MHC II and PD-L1.<br><br>We defined the following immune cells subpopulation from spleen samples as following way: we first gated CD19 and B220 double positive B cells, then compared CD23 to CD1d, or, CD23 to CD21 as marginal zone B cells.<br><br>We defined the in vitro purification macrophages like BMDMs and PMs by directly gating the CD11b and F4/80 double positive cells as macrophages. The macrophages were further gated for MARCO or TLR9 expression, or for checking activation markers like CD206, MHC II and CD86.<br><br>Please refer to Extended Data Figure 9.

☒ Tick this box to confirm that a figure exemplifying the gating strategy is provided in the Supplementary Information.
