# Supplementary material for: Cancer immunotherapy targeting murine myeloid cells requires endosomal pattern recognition
Source: Nat Commun. 2026 Jul 27;17:7410. doi: 10.1038/s41467-026-75543-2 (PMC13408345; doi:10.1038/s41467-026-75543-2)
Supplement: Supplementary file 3 — Transparent Peer Review file [file 41467_2026_75543_MOESM3_ESM.pdf]

nature portfolio

Peer Review File

Cancer immunotherapy targeting murine myeloid cells requires endosomal pattern recognition

Corresponding Author: Professor Mikael Karlsson

This file contains all reviewer reports in order by version, followed by all author rebuttals in order by version.

Version 0:

Reviewer comments:

Reviewer #1

(Remarks to the Author)
In the present manuscript, Pan and colleagues demonstrate that the intracellular TLR signaling machinery, particularly involving TLR9, plays a critical role in mediating the antitumor efficacy of a therapeutic antibody targeting the scavenger receptor MARCO, which is expressed on macrophages. The study is well-conceived, and the data presented convincingly support the authors' conclusions. However, several points need further clarification and elaboration.

General Comments
- A recent study has reported a pro-tumoral role for MARCO+ macrophages in hepatocellular carcinoma. This work demonstrated that MARCO expression in macrophages suppresses type I interferon secretion via a STING-dependent mechanism. Notably, this impaired secretion was rescued through treatment with an anti-MARCO antibody (https://doi.org/10.1016/j.canlet.2023.216568). Moreover, UNC93B1 knockout has been shown to enhance type I interferon release, suggesting a potential regulatory role for UNC93B1 in modulating STING signaling (https://doi.org/10.1002/jmv.27860.). In light of this, have the authors ruled out the possibility that altered STING signaling due to UNC93B1 mutation underlies the observed effects? Assessing STING pathway activity would strengthen the interpretation of their results.
- In a previous publication from this same research group, it was shown that the therapeutic efficacy of the anti-MARCO antibody is dependent on its interaction with the FcgRIIb receptor (http://dx.doi.org/10.1016/j.celrep.2016.04.084). This important finding is not referenced in the current study. At least in B cells, TLR9 signaling influences FcgRIIb expression (https://doi.org/10.1186/1471-2172-9-39). The authors should investigate whether FcgRIIb expression is altered in Unc93b13d/3d macrophages and in the other experimental models used. This would help determine whether the diminished efficacy of anti-MARCO therapy might be due to changes in FcgRIIb expression as a consequence of impaired endosomal TLR signaling.
- TLR9 is classically known to recognize unmethylated CpG motifs in bacterial DNA. However, emerging evidence indicates that it can also sense DAMPs. Since conditioned medium from tumor cells is sufficient to activate macrophages in a TLR9-dependent manner, the authors should clarify what ligand(s) they hypothesize to be responsible for this effect. A more detailed discussion on this point would be valuable.
- Synthetic TLR9 agonists have been explored as adjuvants in cancer immunotherapy. Could the authors consider evaluating a combinatorial approach using anti-MARCO antibody and a TLR9 agonist in the models presented?

Specific Comments

Figure 2
- The data presented are derived from a murine breast cancer model. The authors should confirm these findings in the melanoma model for broader relevance.
- TAMs derived from Unc93b13d/3d mice exhibit a more pro-tumoral phenotype compared to their WT counterparts, as indicated by lower expression of Ccl5, Il12b, and Cxcl9. However, tumor growth curves in WT and Unc93b13d/3d mice (Fig. 1c–d) appear nearly identical, suggesting no functional difference in tumor control. How do the authors explain these findings?
- WT TAMs treated with anti-MARCO show increased expression of Ccl5, Il12b, and Cxcl9 compared to Unc93b13d/3d TAMs, closely resembling the untreated WT vs. Unc93b13d/3d comparison. It would be informative to compare anti-MARCO-treated WT macrophages to untreated WT controls to assess direct gene modulation. Could the authors reanalyze

the data including all four experimental groups simultaneously?

Figure 3
- The gene pathway analysis revealed that Unc93b1 deficiency impacts PD-L1 expression. While it is reasonable that the authors explored the impact of this on anti–PD-L1 therapy, this part of the study disrupts the narrative coherence. The findings are observational, lacking mechanistic insights into why PD-L1 is downregulated in Unc93b1-deficient macrophages. Is this due to reduced activation resulting from impaired endosomal TLR signaling? This point is not adequately addressed in the Discussion. The authors may consider grouping all PD-L1–related data into a single dedicated section.

Figure 4
- What would be the impact of anti-MARCO treatment on T cell proliferation and IFN-γ production when macrophages are polarized with LPS and IFN-γ? This experimental condition should be included.

Figure 5
- Although subsequent data support the proposed role of TLR9 in modulating anti-MARCO immunotherapy efficacy, the initial rationale is weak. The cited study (https://doi.org:10.1084/jem.20070600) refers only to MARCO, with no mention of TLR9. Given prior findings of TLR7 involvement in melanoma and TLR7 activation leading to MARCO upregulation, the authors should rule out a potential role for TLR7 in modulating anti-MARCO efficacy.

Extended Data Fig. 6n–p
- Why was anti–PD-L1 treatment efficacy evaluated only in the melanoma model using MarcoCre+/- Tlr9flox/flox mice, and not in the breast cancer model? An explanation should be provided.

Figure 6
- Why were macrophages from MarcoCre+/- mice not included in the experimental design as a control?
- As with Figure 4, what would be the effect of anti-MARCO on T cell proliferation and IFN-γ production in macrophages polarized with LPS+IFN-γ? This group should be also considered.

Figure 7
- To comprehensively evaluate inflammasome activation, additional markers such as cleaved caspase-1, IL-18, and gasdermin D (GSDMD) should be assessed.

Reviewer #2

(Remarks to the Author)
Pan et al unveil an interesting mechanism through which anti-MARCO antibodies stimulate antitumor immunity, employing several novel mouse strains. TLR9 signalling appears to be necessary to precondition the macrophages, in particular by upregulating the expression of inflammasome-related genes. Upon MARCO triggering, ATP is released, which presumably activates the inflammasome in the TLR9 preconditioned macrophages, releasing IL-1b and stimulated T-cell activation.
Several remarks remain:
1) Fig 1g-i: Upon anti-MARCO treatment, macrophages in the TME adopt an increased anti-tumor phenotype. Is this a general shift in the TAM phenotype, or is there a change in the presence of particular TAM subsets?
2) Fig 2d-e. The authors state: “These findings indicate that endosomal TLR signaling in macrophages modulates their polarization state.” At that point, the mice used are deficient for endosomal TLRs in all cell types. Hence, the conclusion that "endosomal TLR signaling in macrophages modulates their polarization state" is at that point not justified and should be reformulated, as it cannot be excluded that endosomal TLR signaling is needed in other cell types (eg DCs) with an indirect effect on macrophages.
3) The authors first claim that TAMs polarize towards a pro-tumoral state in the absence of endosomal TLR signalling, but subsequently go on to claim that PDL1 expression is lower in cells without endosomal TLR signalling. These are contradictory statements, as PDL1 is a prominent T-cell inactivating molecule.
The interaction between WT/Unc93b1-KO TAMs and T cells requires dedicated experiments to resolve whether this interaction is T-cell stimulatory or suppressive.
4) Moreover, if TAMs are more pro-inflammatory in Unc93b1-deficient mice, why is there no difference in tumor growth? A thorough analysis of the tumor immune compartment is needed.
5) In line with these remarks, the T-cell suppression assay in Fig 4c should be done with TAMs
6) Fig 4c-e: Given the differences in gene expression between WT and Unc93b1-deficient macrophages: do they suppress T cells via the same mechanism?
7) Extended data 6f-j. Whether TLR9 is specifically knocked out in MARCO+ macrophages is not shown. Extended data Fig 6g (left) shows a reduction in TLR9+ F4/80+ cells (while the histogram plot on the right mentions % of MARCO+ PMs, so there is a mistake somewhere). In any case, it is important to show a specific reduction in TLR9 expression in MARCO+ F4/80+ macrophages, but not in MARCO- F4/80+ macrophages.
8) Extended data 6m. So, PD-L1 upregulation in macrophages by endosomal TLRs does not depend on TLR9. Could the responsible endosomal TLR be identified via a treatment of the macrophages with specific ligands?
9) The authors stimulate macrophages with TLR9 agonists and tumor-conditioned medium to induce the expression of inflammasome-related genes. Can this be done with TLR7-8 agonists as well? This would show that the other endosomal TLRs could intrinsically contribute as well, but that B16 tumors only release TLR9 ligands. Can the authors demonstrate that

only TLR9 is triggered by tumor-conditioned medium?
10) The authors speculate that the inflammasome is activated through the TLR9-ATP axis, but this is only shown in an indirect way by the release of IL-1b. Mature IL-1b can also be produced in an inflammasome-independent way, especially in the TME. The authors should directly demonstrate an enhanced NLRP3 inflammasome activation upon anti-MARCO treatment of control versus TLR9 cKO macrophages.

Reviewer #3

(Remarks to the Author)
The study by Pan Y et al provides strong evidence that activity of endosomal TLR signalling is crucial for an efficient anti-tumor response to aMARCO and aPD-L1 treatment in pre-clinical melanoma and breast cancer models. The data suggested that this is linked to an impaired phenotypic switch from an immunosuppressive to a pro-inflammatory state, a transition generally induced in TAMs by aMARCO and aPD-L1 treatment. Transcriptional analysis provides evidence of a “pre-polarized” state of TAMs in wt animals that is based on endosomal TLR activity and a prerequisite for achieving this phenotypic switch by aMARCO and aPD-L1 treatment. Further investigations on the mechanism of action of aMARCO suggested that treatment induced ATP release appears to drive the pro-inflammatory phenotype within this pre-polarized macrophages TAMs but is not active in TLR-9 deficient cells.

The study provides valuable observations but remains largely correlative and provides limited mechanistic insights. Addressing the following aspects in greater detail could help to clarify the underlying mechanism and would substantially strengthen the manuscript.

• While the data generated with mice lacking functional TLR 3,7 and 9 nicely showed that both aPD-L1 and aMARCO treatment require endosomal TLR signalling to be efficient, it lacks clarity on the cell type primarily responsible for the observed effect and the contribution of other cell types remains largely unaddressed.
In addition, TLR deficiency seem to reduce already the abundance of macrophages in the tumor microenvironment, suggesting a generally altered composition of the TME. This aspect should be discussed in more detail

• The performed bulk RNA sequencing gives a first indication on the altered polarization state of TAMs linked to the lack of endosomal TLR activity. However, a more detailed characterization of this “pre-polarized” state applying higher resolution techniques such as single cell RNA seq would be recommended to obtain more granular information on the specific subsets and their respective polarization state.

• A more comprehensive analysis on the factors that drive this “pre-polarized state” (e.g. localization within the TME, tumor-derived factors,….) would support mechanistic understanding of drug efficacy.

• To better understand the link between the aMARCO/aPD-L1 treatment and TLR activity, investigations that explore this “synergy” on a molecular level should be performed.

• While the in vitro data indicate that the presence of aMARCO treated TAMs promotes T cell proliferation it would be interesting to define the molecular mechanism that drives this effect.

• To strengthen the implication of these findings in a more translational context, therapeutic evidence that a synergistic activation of TLR9 and aMARCO treatment lead to enhanced effect should also be addressed.

• The addition of critical controls such as FMO staining controls and isotype controls in the in vitro assays would facilitate a more accurate interpretation of the data.

Version 1:

Reviewer comments:

Reviewer #1

(Remarks to the Author)
The authors have addressed the comments raised during the previous round of review in a thorough and satisfactory manner. The additional data and revisions provided further strengthen the manuscript, clarify relevant aspects of the study, and improve the overall robustness of the work. In light of these changes, I consider the manuscript suitable for publication.

Reviewer #2

(Remarks to the Author)
The authors sufficiently addressed my concerns

Reviewer #3

(Remarks to the Author)

The inclusion of a comprehensive single-cell dataset of immune cell infiltrates in aMARCO treated WT mice and mice lacking functional endosomal Toll-like receptors (TLRs)
provided granular insights into immunotherapy-induced macrophage polarization and subset-specific shifts, including altered signalling pathways and the distinct role of endosomal TLRs in this context. Further, the addition of experiments dissecting the synergism between immunotherapy treatment and endosomal TLR signalling and the inclusion of in vivo data using a combinatorial treatment approach confirming this synergism, further strengthen the manuscript and supports the proposed mechanism.

A more detailed analysis of the newly generated single-cell dataset, particularly with regard to phenotypic changes in other immune cell populations other than monocytes and macrophages, could offer the opportunity to gain even deeper biological insights and address remaining mechanistic questions. While the current analysis supports the author’s conclusions and aligns with the scope of the manuscript, a brief perspective in the discussion could be considered emphasizing this aspect, to enhance the broader impact of the current findings.

Overall, the revised manuscript is notably strengthened and effectively addresses several key concerns raised during revision.

Open Access This Peer Review File is licensed under a Creative Commons Attribution 4.0 International License, which permits use, sharing, adaptation, distribution and reproduction in any medium or format, as long as you give appropriate credit to the original author(s) and the source, provide a link to the Creative Commons license, and indicate if changes were made.
In cases where reviewers are anonymous, credit should be given to 'Anonymous Referee' and the source.
The images or other third party material in this Peer Review File are included in the article’s Creative Commons license, unless indicated otherwise in a credit line to the material. If material is not included in the article’s Creative Commons license and your intended use is not permitted by statutory regulation or exceeds the permitted use, you will need to obtain permission directly from the copyright holder.
To view a copy of this license, visit https://creativecommons.org/licenses/by/4.0/

RESPONSE TO REVIEWERS' COMMENTS

Reviewer #1.
In the present manuscript, Pan and colleagues demonstrate that the intracellular TLR signaling machinery, particularly involving TLR9, plays a critical role in mediating the antitumor efficacy of a therapeutic antibody targeting the scavenger receptor MARCO, which is expressed on macrophages. The study is well-conceived, and the data presented convincingly supports the authors' conclusions.

1. A recent study has reported a pro-tumoral role for MARCO+ macrophages in hepatocellular carcinoma. This work demonstrated that MARCO expression in macrophages suppresses type I interferon secretion via a STING-dependent mechanism. Notably, this impaired secretion was rescued through treatment with an anti-MARCO antibody (https://doi.org/10.1016/j.canlet.2023.216568).

- We thank the reviewer for bringing up this study. The authors show that phagocytosis of dying tumor cells through MARCO suppress type I IFN via the cGAMP-ATP-STING pathway. Using anti-MARCO antibodies, they can block efferocytosis and restore IFN production. In contrast, when used for immunotherapy, anti-MARCO antibodies act through a distinct mechanism, as they are quickly internalized, leading to macrophage activation and repolarization. This includes release of extracellular ATP. Not all anti-MARCO antibodies have this repolarizing capacity, and we have made it clear to state that this antibody works as an agonist throughout the manuscript.

1.2 Moreover, UNC93B1 knockout has been shown to enhance type I interferon release, suggesting a potential regulatory role for UNC93B1 in modulating STING signaling (https://doi.org/10.1002/jmv.27860.). Considering this, have the authors ruled out the possibility that altered STING signaling due to UNC93B1 mutation underlies the observed effects? Assessing STING pathway activity would strengthen the interpretation of their results.

- This study identifies UNC93B1 as a negative regulator of the STING pathway. It achieves this by directly binding to STING and inhibiting the activation of the cGAS-STING axis. A key finding is that UNC93B1 deficiency impairs STING trafficking. They use a knockout of *Unc93b1* in this study which is different from the *Unc93b1*$^{3d/3d}$ (H412R) mutant strain we use. This paper also reports that UNC93B1 deficiency model disrupts STING but that the UNC93B1 H412R mutation specifically does not affect STING signaling. Thus, we believe that there is no difference in involvement of STING in the different groups.

2. In a previous publication from this same research group, it was shown that the therapeutic efficacy of the anti-MARCO antibody is dependent on its interaction with the FcγRIIb receptor (http://dx.doi.org/10.1016/j.celrep.2016.04.084). This important finding is not referenced in the current study. At least in B cells, TLR9 signaling influences FcγRIIb expression

(https://doi.org/10.1186/1471-2172-9-39). The authors should investigate whether FcγRIIb expression is altered in Unc93b13d/3d macrophages and in the other experimental models used. This would help determine whether the diminished efficacy of anti-MARCO therapy might be due to changes in FcγRIIb expression as a consequence of impaired endosomal TLR signaling.

- This is an important point as we found that anti-MARCO treatment is at least partly dependent on FcγRIIb. Thus, we investigated the expression levels of FcγRIIb both in macrophages from peritoneum and from the tumor. The data showed that there was no detectable difference in expression levels, and this data has been added to the manuscript (**Extended Data Fig. 1e**).

3. TLR9 is classically known to recognize unmethylated CpG motifs in bacterial DNA. However, emerging evidence indicates that it can also sense DAMPs. Since conditioned medium from tumor cells is sufficient to activate macrophages in a TLR9-dependent manner, the authors should clarify what ligand(s) they hypothesize to be responsible for this effect. A more detailed discussion on this point would be valuable.

- We thank the reviewer for this suggestion. It is known that necrotic and apoptotic cells release significant amounts of genomic and mitochondrial DNA during cell stress or death that can activate TLR9 signaling (PMID: 29760532; PMID: 22535248). TLR9 can also act as a sensor for tumor-released DNA after chemotherapy (PMID: 31619293). Furthermore, we believe that extracellular DNA exhibits significantly greater stability in the TME compared to RNA, which is rapidly degraded by ubiquitous RNases. Thus, we hypothesize that DNA released from dying tumor cells serves as the primary ligand for TLR9 in our studies. To experimentally validate this hypothesis and distinguish between endosomal TLR signals, we utilized specific antagonists for TLR7 and TLR9. Macrophages were pre-treated with these antagonists prior to stimulation with tumor-conditioned media followed with ATP. Our results demonstrated that TLR9 inhibition significantly reduced IL-1β secretion, while TLR7 inhibition had no observable effect on IL-1β levels. These data shows that TLR9-mediated DNA sensing—rather than TLR7-mediated RNA sensing—is responsible for macrophage activation in our model (**Fig. 8l**). We have also added to the discussion on this point.

4. Synthetic TLR9 agonists have been explored as adjuvants in cancer immunotherapy. Could the authors consider evaluating a combinatorial approach using anti-MARCO antibody and a TLR9 agonist in the models presented?

- This is a good suggestion and logical addition to our studies. Thus, we have evaluated the therapeutic potential of a combinatorial approach using anti-MARCO antibody and a TLR9 agonist (CpG ODN) in both the B16-F10 melanoma and EO771 breast cancer models. Our data demonstrates that combination therapy provides superior anti-tumor efficacy compared to monotherapy. Specifically, we observed a significant reduction in tumor burden with a robust increase in CD8+ T cells infiltration from the combo treatment group, suggesting a synergistic interaction between MARCO and TLR9-mediated macrophage activation (**Fig. 6g,h and Extended Data Fig. 6r**).

5. Figure 2 - The data presented are derived from a murine breast cancer model. The authors should confirm these findings in the melanoma model for broader relevance.

- This is a valid point, and we have now performed additional experiments. To address this, we sorted tumor-associated macrophages (TAMs) from B16-F10 melanoma tumors and performed qPCR to evaluate the expression of the key differentially expressed genes initially identified in the breast cancer model, including *Cxcl9*, *Ccl5*, *Cd274*, *Il12*, *Mrc1* and *Spp1*. We found that that the gene expression profiles in the melanoma-derived macrophages are consistent with those observed in the murine breast cancer model (**Data for reviewer**). Still, we realize that the resolution for our bulk RNA-seq experiment left some unanswered questions and have now performed a single cell RNA-seq experiment and added this to the

manuscript (**Fig. 2**). As this provides a more detailed evaluation of the changes in the TME we have excluded the immunotherapy data from the bulk RNAseq experiments.

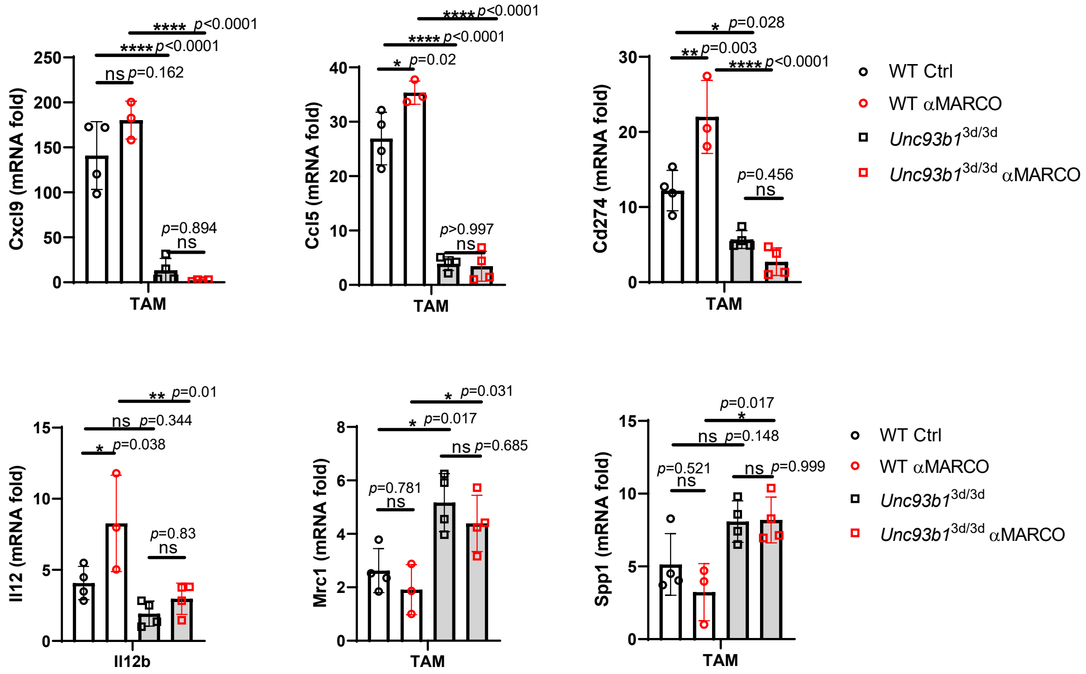

6. TAMs derived from Unc93b13d/3d mice exhibit a more pro-tumoral phenotype compared to their WT counterparts, as indicated by lower expression of Ccl5, Il12b, and Cxcl9. However, tumor growth curves in WT and Unc93b13d/3d mice (Fig. 1c–d) appear nearly identical, suggesting no functional difference in tumor control. How do the authors explain these findings?

- We thank the reviewer for brining this up and we believe that there are several reasons behind this: First, TAMs represent a highly heterogeneous population comprising both pro- and anti-tumoral subsets. Supporting this, our pathway analysis revealed an enrichment of PD-L1–related signaling in WT TAMs, indicating the coexistence of immunosuppressive programs alongside pro-inflammatory features. Thus, despite differences in specific gene expression, we believed that the overall suppression imposed by the TAMs is similar even though *Unc93b1* is mutated. Importantly, the differences become evident when applying immunotherapy to target the myeloid cells. Second, the suppressive forces within the TME are not always linear and we find that when cytotoxic lymphocytes get past the threshold of activation the effect is often cascading. Still, to further examine the TAMs with *Unc93b1* mutations with and without treatment, we also performed single-cell RNA sequencing (scRNA-seq) experiments, which provide higher-resolution insights into their functional states, and these are presented in **Fig. 2**. In addition, we have changed the text as well as added to the discussion on this point.

7. WT TAMs treated with anti-MARCO show increased expression of Ccl5, Il12b, and Cxcl9 compared to Unc93b13d/3d TAMs, closely resembling the untreated WT vs. Unc93b13d/3d comparison. It would be informative to compare anti-MARCO-treated WT macrophages to untreated WT controls to assess direct gene modulation. Could the authors reanalyze the data including all four experimental groups simultaneously?

- While a direct comparison using our bulk RNA-seq dataset is precluded by technical batch effects (the two sample sets were processed and sequenced at different times), we decided to address this question using a higher-resolution approach. We performed scRNA-seq with all four experimental groups processed simultaneously, eliminating batch effect concerns. Because scRNA-seq provides greater cellular resolution, it allows us to more accurately

assess the macrophage responses. Because of these new data we also decided to exclude the immunotherapy data from the bulk RNAseq experiments.

8. Figure 3: The gene pathway analysis revealed that Unc93b1 deficiency impacts PD-L1 expression. While it is reasonable that the authors explored the impact of this on anti–PD-L1 therapy, this part of the study disrupts the narrative coherence. The findings are observational, lacking mechanistic insights into why PD-L1 is downregulated in Unc93b1-deficient macrophages. Is this due to reduced activation resulting from impaired endosomal TLR signaling? This point is not adequately addressed in the Discussion. The authors may consider grouping all PD-L1–related data into a single dedicated section.

- This was a surprising finding, and we thought since anti-PD-L1 antibodies bind not only TAMs but also other PD-L1 expressing populations, separate from anti-MARCO antibodies, it would be informative to compare them. To add to this, we have now done *in vitro* experiments and find that expression of PD-L1 is dependent on TLR3 and TLR7 but not TLR9 (**Extended data Fig. 5d**). We appreciate the suggestion to put the anti-PDL1 data in a separate section, but we found it hard with the narrative as we use the treatment throughout the manuscript using the conditionally targeted mice. We have gone through the manuscript to make it more coherent and added to the discussion on this point.

9. Figure 4: What would be the impact of anti-MARCO treatment on T cell proliferation and IFN-γ production when macrophages are polarized with LPS and IFN-γ? This experimental condition should be included.

- We agree and have polarized macrophages with LPS and IFN-γ to induce a pro-inflammatory phenotype, followed by anti-MARCO treatment and co-culture with T cells. We found that under these conditions, anti-MARCO treatment had no significant effect on T cell proliferation or IFN-γ release compared to untreated controls. (**Extended Data Fig. 4c,d**).

10. Figure 5: Although subsequent data supports the proposed role of TLR9 in modulating anti-MARCO immunotherapy efficacy, the initial rationale is weak. The cited study (https://doi.org:10.1084/jem.20070600) refers only to MARCO, with no mention of TLR9. Given prior findings of TLR7 involvement in melanoma and TLR7 activation leading to MARCO upregulation, the authors should rule out a potential role for TLR7 in modulating anti-MARCO efficacy.

- It is known that dying cells in tumors will release DNA that is often hypomethylated (PMID: 31619293, 38767879, 24154870). Thus, we hypothesized that DNA released from spontaneous necrosis/apoptosis or extracellular vesicles serves as the primary ligand for TLR9 in the TME. We now performed additional experiments and first, to distinguish between endosomal TLR signals, we used TLR7 agonist and found it can also induce IL-1β secretion (**Fig. 8k**). Second, we used specific antagonists for TLR7 and TLR9. Macrophages were pre-treated with these antagonists prior to stimulation with tumor-conditioned media followed with ATP. Our results demonstrated that TLR9 inhibition significantly reduced IL-1β secretion, while TLR7 inhibition had no effect on IL-1β levels. These data show that TLR9-mediated DNA sensing—rather than TLR7-mediated RNA sensing—is dominant to induce macrophage activation in our model (**Fig. 8l**). We have added this data and included a discussion on this point.

11. Extended Data Fig. 6n–p: Why was anti–PD-L1 treatment efficacy evaluated only in the melanoma model using MarcoCre+/- Tlr9flox/flox mice, and not in the breast cancer model? An explanation should be provided.

- This is a valid point and we now added data from anti–PD-L1 treatment of the breast cancer model in $Marco^{Cre+/-}$ $Tlr9^{flox/flox}$ mice. Consistent with the melanoma model, anti-PD-L1

treatment also reduces tumor burden both in the litter mate control and *Marco*$^{Cre+/-}$ *Tlr9*$^{flox/flox}$ mice in the breast cancer model (**Extended Data Fig. 6o-q**).

12. Figure 6: Why were macrophages from MarcoCre+/- mice not included in the experimental design as a control?

- This is an important control and we generated *Marco*$^{Cre+/-}$ mice to be used as littermate control and performed the co-culture experiment. It shows the same phenotype as *Tlr9*$^{flox/flox}$ mice (**Extended Data Fig. 7a,b**).

13. As with Figure 4, what would be the effect of anti-MARCO on T cell proliferation and IFN-γ production in macrophages polarized with LPS+IFN-γ? This group should be also considered.

- As shown in the new data presented for Figure 4, anti-MARCO treatment did not alter T cell proliferation or IFN-γ release when co-cultured with LPS/IFN-γ polarized macrophages from both WT and *Unc93b1*$^{3d/3d}$ mice. We repeated this experiment using LPS/IFN-γ polarized macrophages derived from littermate controls or *Marco*$^{Cre+/-}$ *Tlr9*$^{flox/flox}$ mice. Consistent with WT controls, anti-MARCO treatment yielded no differences in T cell proliferation or IFN-γ release in both littermate controls and *Marco*$^{Cre+/-}$ *Tlr9*$^{flox/flox}$ co-cultures system (**Extended Data Fig. 7a,b**).

14. Figure 7: To comprehensively evaluate inflammasome activation, additional markers such as cleaved caspase-1, IL-18, and gasdermin D (GSDMD) should be assessed.

- We thank the reviewer for this suggestion, and we have now added additional data to strengthen this point. We stimulated macrophages with TLR9 agonist or tumor-conditioned media followed with ATP and performed a series of new assays to assess the requested markers alongside core inflammasome components. First, to directly evaluate caspase-1 activation, we utilized a fluorescent labeled inhibitor of caspases (FLICA) assay measured using flow cytometry. We observed a significant reduction in active caspase-1 (FLICA+) cells in the *Marco*$^{Cre+/-}$ *Tlr9*$^{flox/flox}$ macrophages compared to littermate controls (**Fig. 8h**). Second, we assessed GSDMD and upstream inflammasome components via Western blot. While baseline GSDMD expression levels were comparable between littermate controls and *Marco*$^{Cre+/-}$ *Tlr9*$^{flox/flox}$ macrophages, we found that the expression of critical upstream inflammasome components, including pro-IL-1β, NLRP3 and ASC, was lower in the *Marco*$^{Cre+/-}$ *Tlr9*$^{flox/flox}$ group. IL-18 production was not detectable in this model (**Fig. 8g**).

Reviewer #2

Pan et al unveils an interesting mechanism through which anti-MARCO antibodies stimulate antitumor immunity, employing several novel mouse strains. TLR9 signalling appears to be necessary to precondition the macrophages, in particular by upregulating the expression of inflammasome-related genes. Upon MARCO triggering, ATP is released, which presumably activates the inflammasome in the TLR9 preconditioned macrophages, releasing IL-1b and stimulated T-cell activation. Several remarks remain:

1) Fig 1gi: Upon anti-MARCO treatment, macrophages in the TME adopt an increased anti-tumor phenotype. Is this a general shift in the TAM phenotype, or is there a change in the presence of particular TAM subsets?

- To answer this question, we performed scRNA-seq on anti-MARCO treated mice and controls in both WT and *Unc93b1* mutated mice (**Fig 2**). Unsupervised clustering identified five transcriptionally distinct TAM clusters, including *H2-Ab1*$^{+}$, *Spp1*$^{+}$, *Isg15*$^{+}$, *Cd80/Cd86*$^{+}$, *Mki67*$^{+}$, and one additional cluster of *Ly6c2*$^{+}$ monocytes. Of these, the most pronounced shift in cell population was found in the *Spp1*$^{+}$TAMs. We subsequently investigated changes in macrophage signaling pathways as described in Schubert et al. (PMID: 29295995). Using

this framework, we found that anti-tumoral transcriptional repolarization was present across all the TAM clusters. This was characterized by broad elevation of TNFα and NF-κB pathway activity and decreased PI3K pathway activity across the different subclusters of TAMs (PMID: 41759521). To further quantify polarization, we calculated macrophage polarization scores using the framework described by Cui et al (PMID: 38057668**)**. Among all cluster, especially *H2-Ab1*$^{+}$, *Spp1*$^{+}$ and *Mki67*$^{+}$, anti-MARCO treatment significantly increased macrophage scores correlating with pro-inflammatory phenotype (PMID: 39999031) We also found that all these transcriptional and polarization changes were largely absent in TAMs from *Unc93b1*$^{3d/3d}$ animals, showing that the shift toward an anti-tumor phenotype depends on intact UNC93B1.

2) Fig 2d-e. The authors state: “These findings indicate that endosomal TLR signaling in macrophages modulates their polarization state.” At that point, the mice used are deficient for endosomal TLRs in all cell types. Hence, the conclusion that "endosomal TLR signaling in macrophages modulates their polarization state" is at that point not justified and should be reformulated, as it cannot be excluded that endosomal TLR signaling is needed in other cell types (eg DCs) with an indirect effect on macrophages.

-This is correct and we have now changed the text and replaced Figure 2 and moved the previously presented data to supplemental material.

3) The authors first claim that TAMs polarize towards a pro-tumoral state in the absence of endosomal TLR signalling, but subsequently go on to claim that PDL1 expression is lower in cells without endosomal TLR signalling. These are contradictory statements, as PDL1 is a prominent T-cell inactivating molecule.

- We agree with the reviewer that this was not clear. We have rephrased the text and also added to the discussion on this point. TAMs represent a highly heterogeneous population with a mixture of both pro- and anti-tumoral states. Consistent with this, our scRNA-seq data show that the TAMs composed of both pro- and anti-tumor polarization states but retain their overall suppressive phenotype in absence of endosomal TLRs when applying myeloid targeted treatment. Importantly, the finding here suggests that the “base line” expression of PD-L1 is lower in *Unc93b1* mutated TAMs, rendering them less targetable by anti–PD-L1 therapies. We have clarified this in the text.

The interaction between WT/Unc93b1-KO TAMs and T cells requires dedicated experiments to resolve whether this interaction is T-cell stimulatory or suppressive.

- To address this, we stimulated WT and *Unc93b1*$^{3d/3d}$ BMDMs with tumor-conditioned media and performed co-culture experiments with T cells. Compared to the pro-inflammatory stimuli (IFN-γ and LPS), the tumor-conditioned media treated macrophages reduced IFN-γ release and proliferation to the same extent as co-cultures using anti-inflammatory macrophages (polarized with IL4/IL10) in both WT and *Unc93b1*$^{3d/3d}$ groups. (**Extended Data Fig. 4c-d**).

4) Moreover, if TAMs are more pro-inflammatory in Unc93b1-deficient mice, why is there no difference in tumor growth? A thorough analysis of the tumor immune compartment is needed.

- We agree that there would be several possible reasons behind this: First, TAMs represent a highly heterogeneous population comprising both pro- and anti-tumoral subsets. Thus, despite differences in specific gene expression, we believe that TAMs from both WT and *Unc93b1* mutation retain the suppressive phenotype and that the difference is only revealed when applying myeloid targeted treatment. Second, the suppressive forces within the TME are not always linear and we find that when cytotoxic lymphocytes get past the threshold of activation the effect is often cascading. To further examine the TAMs with *Unc93b1* mutations with and without treatment, we performed scRNA-seq experiments, which provide higher-

resolution insights into their functional states, and these are presented in **Fig. 2**. We have changed the text in results and discussion to clarify this point.

5) In line with these remarks, the T-cell suppression assay in Fig 4c should be done with TAMs

- To address this, we isolated TAMs from tumors from both WT and *Unc93b1*$^{3d/3d}$ mice with or without anti-MARCO treatment and cocultured these with T cells. The data showed that only macrophage from WT treated with anti-MARCO can significantly induce T cells proliferation and IFN-γ release. On the other hand, the macrophages from *Unc93b1*$^{3d/3d}$ could not restore T cell proliferation and IFN-γ release (**Fig. 4f**).

6) Fig 4c-e: Given the differences in gene expression between WT and Unc93b1-deficient macrophages: do they suppress T cells via the same mechanism?

- Anti-Inflammatory TAMs have several suppressive mechanisms at their disposal. Thus, it is likely that some of these overlap between WT and Unc93b1 mutated mice while some may play a more significant role in either setting. In the scRNA-seq experiment, we analyzed the ligand-receptor pairs of both MHC I and MHC II from the six TAM/monocyte clusters to the T cells and NK cells by utilizing CellChat analysis and found that the overall interaction of TAM and lymphocytes was similar between WT and Unc93b1 mutated mice (**Fig 2g,h and Extended Data Fig. 2j,k**).

7) Extended data 6f-j. Whether TLR9 is specifically knocked out in MARCO+ macrophages is not shown. Extended data Fig 6g (left) shows a reduction in TLR9+ F4/80+ cells (while the histogram plot on the right mentions % of MARCO+ PMs, so there is a mistake somewhere). In any case, it is important to show a specific reduction in TLR9 expression in MARCO+ F4/80+ macrophages, but not in MARCO- F4/80+ macrophages.

- This is an important point. We show that the MARCOcre is functional by both the expression of a floxed reporter (**Extended Data Fig. 6c**) as well as that TLR9 is greatly reduced in MARCO expressing macrophages specifically and the overall MARCO expression is not reduced by the introduction of cre (**Extended data Fig 6e-g**). Here we compared the percentage of TLR9+ cells out of total MARCO+ PMs or other non-macrophages. Also, we have verified that TLR9 agonist can induce MARCO expression in BMDMs (**Fig. 5e**). Then we used the TLR9 agonist to stimulate BMDMs from littermate controls or TLR9 specifically knockout BMDMs and found the MARCO expression is specifically inhibited in the TLR9 conditioned target BMDMs (**Extended data Fig 6h**). We have changed the text to make it clear in figure legend.

8) Extended data 6m. So, PD-L1 upregulation in macrophages by endosomal TLRs does not depend on TLR9. Could the responsible endosomal TLR be identified via a treatment of the macrophages with specific ligands?

- To investigate this, we used TLR3, TLR7 and TLR9 agonist to stimulate BMDMs, and found that TLR3 and TLR7 agonist can upregulate PD-L1 expression, while TLR9 agonist does not and this upregulation was also lost in the Unc93b1 mutated macrophages (**Extended Data Fig. 5d**).

9) The authors stimulate macrophages with TLR9 agonists and tumor-conditioned medium to induce the expression of inflammasome-related genes. Can this be done with TLR7-8 agonists as well? This would show that the other endosomal TLRs could intrinsically contribute as well, but that B16 tumors only release TLR9 ligands. Can the authors demonstrate that only TLR9 is triggered by tumor-conditioned medium?

- To address this, we first used a TLR7 agonist and found it can also induce IL-1β secretion (**Fig. 8k**). However, when we used antagonists for TLR7 (M5049) or TLR9 (ODN INH-18) by pre-treating the macrophage and following tumor media with ATP stimulation, only the TLR9

antagonists reduced the IL-1β secretion, showing that TLR9 activation is responsible for mediating the macrophage activation (**Fig. 8l**).

10) The authors speculate that the inflammasome is activated through the TLR9-ATP axis, but this is only shown in an indirect way by the release of IL-1b. Mature IL-1b can also be produced in an inflammasome-independent way, especially in the TME. The authors should directly demonstrate an enhanced NLRP3 inflammasome activation upon anti-MARCO treatment of control versus TLR9 cKO macrophages.

-We agree that this is an important point. Thus, we stimulated macrophages with TLR9 agonist and tumor-conditioned media followed with ATP or anti-MARCO antibody and assessed inflammasome components using Western blot for all conditions. We found the anti-MARCO antibody can induce inflammasome activation, including pro-IL-1β, NLRP3 and ASC, to the same extent as ATP induced induction (**Fig. 8g**). We also verified this *in vivo* that anti-MARCO treatment significantly increased IL-1β secretion in control tumors but not from TLR9 conditional knockout tumors (**Fig. 8n**).

Reviewer #3

The study by Pan Y et al provides strong evidence that activity of endosomal TLR signalling is crucial for an efficient anti-tumor response to aMARCO and aPD-L1 treatment in pre-clinical melanoma and breast cancer models. The data suggested that this is linked to an impaired phenotypic switch from an immunosuppressive to a pro-inflammatory state, a transition generally induced in TAMs by aMARCO and aPD-L1 treatment. Transcriptional analysis provides evidence of a “pre-polarized” state of TAMs in wt animals that is based on endosomal TLR activity and a prerequisite for achieving this phenotypic switch by aMARCO and aPD-L1 treatment. Further investigations on the mechanism of action of aMARCO suggested that treatment induced ATP release appears to drive the pro-inflammatory phenotype within this pre-polarized macrophages TAMs but is not active in TLR-9 deficient cells.The study provides valuable observations but remains largely correlative and provides limited mechanistic insights. Addressing the following aspects in greater detail could help to clarify the underlying mechanism and would substantially strengthen the manuscript.

1. While the data generated with mice lacking functional TLR 3,7 and 9 nicely showed that both aPD-L1 and aMARCO treatment require endosomal TLR signalling to be efficient, it lacks clarity on the cell type primarily responsible for the observed effect and the contribution of other cell types remains largely unaddressed.

-To address this, we have now added data where we use scRNA-seq to investigate the cellular response to anti-MARCO. The data is now presented in **Figure 2**. In addition, the expression of TLR3, TLR7, and especially TLR9 are mostly specific to monocyte/ macrophage populations, as previously reported (PMID: 40215981, PMID: 36658237). We find that several of the TAM populations respond to anti-MARCO treatment and repolarizes towards a pro-inflammatory phenotype. Also, we analyzed the ligand-receptor pairs of both MHC I and MHC II from the six TAM/monocyte clusters to the T cells and NK cells and found that the overall interaction was primarily directed towards exhausted T cells and regulatory T cells, but after anti-MARCO treatment, these interactions were reduced and redirected towards naïve and proliferating T cells.

2. In addition, TLR deficiency seems to reduce already the abundance of macrophages in the tumor microenvironment, suggesting a generally altered composition of the TME. This aspect should be discussed in more detail

- It is true that in some experiments we found fewer TAMs regarding the cell numbers. However, the percentage of total macrophages is similar between WT and Unc93b1 mutated mice. The scRNA-seq data also showed the similar macrophage composition between WT and Unc93b1 mutated mice. Our data suggests that the overall suppressive capacity of the

TAMs relates to their polarization states rather than their numbers. We have now added this point to the discussion and results section.

3. The performed bulk RNA sequencing gives a first indication on the altered polarization state of TAMs linked to the lack of endosomal TLR activity. However, a more detailed characterization of this "pre-polarized" state applying higher resolution techniques such as single cell RNA seq would be recommended to obtain more granular information on the specific subsets and their respective polarization state.

- To address this, we performed scRNA-seq on anti-MARCO treated mice and controls in both WT and *Unc93b1* mutated mice (**Fig. 2**). Unsupervised clustering identified five transcriptionally distinct TAM clusters, including *H2-Ab1*$^{+}$, *Spp1*$^{+}$, *Isg15*$^{+}$, *Cd80/Cd86*$^{+}$, *Mki67*$^{+}$, and one additional cluster of *Ly6c2*$^{+}$ monocytes. Of these the most pronounced shift in cell population was found in *Spp1*+TAMs. We subsequently investigated changes in macrophage signaling pathways as described in Schubert et al. (PMID: 29295995). Using this framework, we found that anti-tumoral transcriptional repolarization was present across all the TAM clusters. This was characterized by broad elevation of TNFα and NF-κB pathway activity and decreased PI3K pathway activity across the different subclusters of TAMs (PMID: 41759521). To further quantify polarization, we calculated macrophage polarization scores using the framework described by Cui et al (PMID: 38057668**)**. Among all clusters, especially *H2-Ab1*$^{+}$, *Spp1*$^{+}$ and *Mki67*$^{+}$, anti-MARCO treatment significantly increased macrophage scores correlating with pro-inflammatory phenotype (PMID: 3999903). We also found that all these transcriptional and polarization changes were largely absent in TAMs from *Unc93b1*$^{3d/3d}$ animals, showing that the shift toward an anti-tumor phenotype depends on intact UNC93B1.

4. A more comprehensive analysis on the factors that drive this "pre-polarized state" (e.g. localization within the TME, tumor-derived factors) would support mechanistic understanding of drug efficacy.

- To address this, we performed some blocking experiments and found that inflammasome activation is dependent on TLR9 ligands present in the tumor supernatant (**Fig 8k,l**). It is also known that necrotic and apoptotic cells release significant amounts of genomic and mitochondrial DNA during cell stress or death that can activate TLR9 signaling (PMID: 29760532; PMID: 22535248). TLR9 can also act as a sensor for tumor-released DNA after chemotherapy (PMID: 31619293). Furthermore, we believe that extracellular DNA exhibits significantly greater stability in the TME compared to RNA, which is rapidly degraded by ubiquitous RNases. We have added this point in the discussion as well as a reference to the localization of the MARCO+ TAMs in the tumor (PMID: 33229588)

5. To better understand the link between the aMARCO/aPD-L1 treatment and TLR activity, investigations that explore this "synergy" on a molecular level should be performed.

- We agree and to do this we used TLR3, TLR7 and TLR9 agonist stimulate BMDMs, and found only TLR3 and TLR7 agonist can upregulate PD-L1 expression, while TLR9 agonist did not (**Extended Data Fig. 5d**). As for the MARCO expression, we found only TLR7 and TLR9 can upregulate MARCO expression, while TLR3 stimulation had no effect. We have added this point in the discussion.

6. While the in vitro data indicates that the presence of aMARCO treated TAMs promotes T cell proliferation it would be interesting to define the molecular mechanism that drives this effect.

- We have added data that addresses this in two ways. First the scRNA-seq experiment shows the dynamics of the T cell response after anti-MARCO treatment with reduced Tregs and shift away from an exhausted phenotype (**Fig. 2**). In addition, we have performed co-culture experiments where we isolated TAMs from tumors from mice with or without anti-

MARCO treatment and cocultured these with T cells. The data showed that only macrophage from WT treated with anti-MARCO can significantly induce T cells proliferation and IFN-γ release. On the other hand, the macrophages from *Unc93b1*$^{3d/3d}$ could not restore T cell proliferation and IFN-γ release (**Fig. 4f**). We also report that TLR9 was needed to prepare TAMs to respond to anti-MARCO by regulating the expression of inflammasome components and readying the macrophages to respond to treatment.

7. To strengthen the implication of these findings in a more translational context, therapeutic evidence that a synergistic activation of TLR9 and aMARCO treatment lead to enhanced effect should also be addressed.

- We thank the reviewer for this suggestion. We have now evaluated the therapeutic potential of a combinatorial approach using anti-MARCO antibody and a TLR9 agonist in both the B16-F10 melanoma and EO771 breast cancer models. The data shows that combination therapy provides better anti-tumor efficacy compared to monotherapy. Specifically, we observed a significant reduction in tumor burden with an increase in CD8+ T cells infiltration from the combo treatment group, suggesting a synergistic interaction between MARCO and TLR9-mediated macrophage activation (**Fig. 6g,h and Extended Data Fig. 6r**).

8. The addition of critical controls such as FMO staining controls and isotype controls in the *in vitro* assays would facilitate a more accurate interpretation of the data.

-This has now been added to the manuscript (**Extended Fig 9**).

RESPONSE TO REVIEWERS' COMMENTS

Reviewer #1:

The authors have addressed the comments raised during the previous round of review in a thorough and satisfactory manner. The additional data and revisions provided further strengthen the manuscript, clarify relevant aspects of the study, and improve the overall robustness of the work. In light of these changes, I consider the manuscript suitable for publication.

Reviewer #2:

The authors sufficiently addressed my concerns

Reviewer #3:

The inclusion of a comprehensive single-cell dataset of immune cell infiltrates in aMARCO treated WT mice and mice lacking functional endosomal Toll-like receptors (TLRs)

provided granular insights into immunotherapy-induced macrophage polarization and subset-specific shifts, including altered signalling pathways and the distinct role of endosomal TLRs in this context. Further, the addition of experiments dissecting the synergism between immunotherapy treatment and endosomal TLR signalling and the inclusion of in vivo data using a combinatorial treatment approach confirming this synergism, further strengthen the manuscript and supports the proposed mechanism.

A more detailed analysis of the newly generated single-cell dataset, particularly with regard to phenotypic changes in other immune cell populations other than monocytes and macrophages, could offer the opportunity to gain even deeper biological insights and address remaining mechanistic questions. While the current analysis supports the author's conclusions and aligns with the scope of the manuscript, a brief perspective in the discussion could be considered emphasizing this aspect, to enhance the broader impact of the current findings. Overall, the revised manuscript is notably strengthened and effectively addresses several key concerns raised during revision.

- We thank the reviewer for this comment and suggestion. We agree that there is more interesting information to be gained from the novel scRNA data set that we generated. This will be available online and we hope that others besides us will use it to further investigate the effects of myeloid targeted treatment also beyond phagocytes and T cells. We have now, as suggested, added a note in the discussion to emphasize this (page 17).
